# Modulation of Caecal Microbiota and Metabolome Profile in *Salmonella*-Infected Broilers by Phage Therapy

**DOI:** 10.3390/ijms242015201

**Published:** 2023-10-15

**Authors:** Laura Lorenzo-Rebenaque, Cristina Casto-Rebollo, Gianfranco Diretto, Sarah Frusciante, Juan Carlos Rodríguez, María-Paz Ventero, Carmen Molina-Pardines, Santiago Vega, Clara Marin, Francisco Marco-Jiménez

**Affiliations:** 1 Department of Animal Production and Health, Veterinary Public Health and Food Science and Technology, Biomedical Research Institute, Faculty of Veterinary Medicine, Cardenal Herrera-CEU University, CEU Universities, Calle Santiago Ramón y Cajal 20, Alfara del Patriarca, 45115 Valencia, Spain; laura.lorenzorebenaque@uchceu.es (L.L.-R.); svega@uchceu.es (S.V.); clara.marin@uchceu.es (C.M.); 2 Institute for Animal Science and Technology, Universitat Politècnica de València, 46022 Valencia, Spain; cricasre@posgrado.upv.es; 3 Italian Agency for New Technologies, Energy and Sustainable Development (ENEA), Biotechnology Laboratory, Centro Ricerche Casaccia, Via Anguillarese, 301, Santa Maria di Galeria, 00123 Rome, Italy; gianfranco.diretto@enea.it (G.D.); sarah.frusciante@enea.it (S.F.); 4 Microbiology Department, Dr. Balmis University General Hospital, Microbiology Division, Miguel Hernández University, ISABIAL, 03010 Alicante, Spain; rodriguez_juadia@gva.es; 5 Microbiology Department, Dr. Balmis University General Hospital, ISABIAL, 03010 Alicante, Spain; maripazvm@gmail.com (M.-P.V.); carmenmolinapardines@gmail.com (C.M.-P.)

**Keywords:** bacteriophages, poultry, *Salmonella*, omic sciences, high-throughput sequencing, microbiome

## Abstract

Bacteriophage therapy is considered one of the most promising tools to control zoonotic bacteria, such as *Salmonella*, in broiler production. Phages exhibit high specificity for their targeted bacterial hosts, causing minimal disruption to the niche microbiota. However, data on the gut environment’s response to phage therapy in poultry are limited. This study investigated the influence of *Salmonella* phage on host physiology through caecal microbiota and metabolome modulation using high-throughput 16S rRNA gene sequencing and an untargeted metabolomics approach. We employed 24 caecum content samples and 24 blood serum samples from 4-, 5- and 6-week-old broilers from a previous study where *Salmonella* phages were administered via feed in *Salmonella*-infected broilers, which were individually weighed weekly. Phage therapy did not affect the alpha or beta diversity of the microbiota. Specifically, we observed changes in the relative abundance of 14 out of the 110 genera using the PLS-DA and Bayes approaches. On the other hand, we noted changes in the caecal metabolites (63 *up-accumulated* and 37 *down-accumulated* out of the 1113 caecal metabolites). Nevertheless, the minimal changes in blood serum suggest a non-significant physiological response. The application of *Salmonella* phages under production conditions modulates the caecal microbiome and metabolome profiles in broilers without impacting the host physiology in terms of growth performance.

## 1. Introduction

*Salmonella* is one of the most frequently isolated foodborne pathogens worldwide. Currently, this bacterium has been linked with 3% of bacterial foodborne diseases, accounting for 80 million infections and 155,000 deaths globally [1,2]. In Europe, despite the efforts to control this pathogen in poultry production, poultry products continue to be the primary source of infection [3]. Indeed, broilers can acquire the bacteria and not exhibit any clinical illness, as it is a silent source of infection [2]. Carrier animals are a silent infection source not only for other broilers in co-housing facilities but also for the processing facilities, with the human health hazards that this entails [2,4]. Despite the biosecurity practices implemented on farms, *Salmonella* control remains a major challenge worldwide, and new alternatives are still necessary [2,5,6]. In this context, bacteriophage therapy has been postulated as one of the most promising tools to control zoonotic bacteria in broilers [7,8,9,10,11]. Indeed, several commercial phages against *Salmonella* in the poultry industry are available (Bafasal^®^, Biotector^®^S, SalmoFreshTM, SalmoPro^®^, SalmonelexTM (PhageGuard), PhageGuard STM, BacWashTM and SalmoFREE^®^).

Bacteriophages (or phages) are viruses that selectively infect and replicate in their target bacterial host. Phage therapy has been considered a promising tool in eliminating bacterial infections in poultry [7]. Compared to antibiotics, phages are highly specific and usually attack only their targeted bacterial hosts, indicating minimal disruption to the niche microbiota [12,13]. The application of phages as a control tool emerges as a promising approach not only as an animal treatment alternative to antibiotics, which has been studied in an extensive body of work [14,15,16,17,18,19,20,21]. In contrast, an indiscriminately broad spectrum of antibiotics runs a high risk of exerting an “imbalance” in the gut commensal microbial community (dysbiosis) [22]. In this context, there are few data on the effects of the clearance of pathogenic bacteria on the gut environment after phage therapy in poultry [11,23]. The gut microbiome is a complex ecosystem that comprises an extremely large number of different indigenous bacteria, archaea, bacteriophages, eukaryotes, viruses and fungi, and it acts as a key intermediate between environmental inputs and host metabolism [24,25]. Cumulative evidence shows that the microbiome plays a crucial role in important metabolic functions, with a great influence on host biological functions, health states and disease progression and performance [24,25]. Indeed, the host and gut microbiota influence each other through a metabolic axis via small molecule metabolites and co-metabolites [24]. In this context, the study of circulating metabolites through metabolomics facilitates an understanding of the mechanisms of the biological and biochemical processes in complex systems that could impinge on the well-being and production of livestock [26,27]. Moreover, the gut microbiota unquestionably plays a critical role in the successful colonisation and infection development caused by enteric pathogens, such as *Salmonella* [28]. Indeed, *Salmonella* strongly interacts with the chicken gut microbiome, altering the microbiota’s composition and richness [11,28]. Therefore, the integrative analysis of caecal microbiota and metabolite profiles of caeca and serum can help to understand the changes in the host’s physiological condition under a *Salmonella* infection and the impact of the phage therapy [11]. To this end, the present study investigated the influence of *Salmonella* phage on host physiology through the modulation of the microbiota and the caecal metabolome in late-stage broiler rearing.

## 2. Results

### 2.1. Effects of Phage on Body Weight

**Figure 1:**
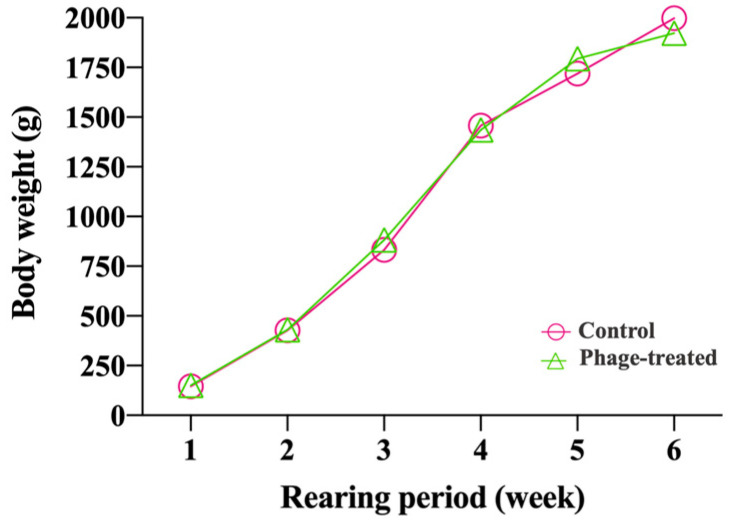
Weekly body weight of broilers (g/animal) in the phage-treated (green) and control (pink) groups. Phage-treated group (green) received 0.1% *Salmonella* phage (10^8^ PFU/g). The control (pink) group did not receive phages.

The samples were obtained from a prior experiment that involved the application of phages for *Salmonella* control during the broiler rearing period [18], where a total of 100 one-day-old *Salmonella*-free male Ross broilers were randomly divided into two treatment groups of 50 animals each (phage-treated and non-phage (control)). There were no significant differences in the body weight of the birds among treatments during the rearing period (*p* value > 0.05) (Figure 1).

### 2.2. Effects of Phage on Caecal Microbiota

High-throughput sequencing obtained 3,258,381 raw sequencing reads (average 161,928.5 reads/sample), with an average read length of 403.8 ± 13.18 pb. After denoising, removing chimeras and filtering low-quality sequences, a total of 2,201,366 sequences and 1049 ASVs were generated. After filtering, a total of 681 ASVs remained for taxonomic assignment.

**Figure 2:**
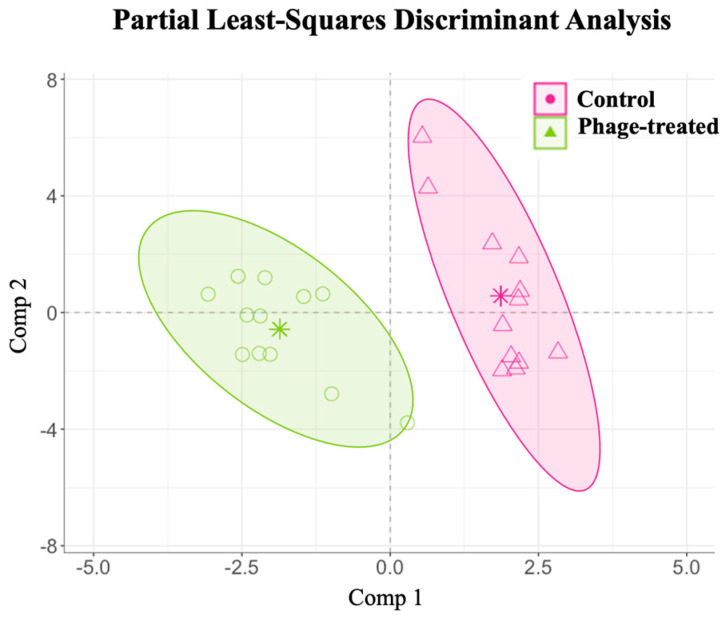
Examining the effects of *Salmonella* phage on the caecal microbiota in *Salmonella*-infected broilers. Caecal microbiota composition dissimilarity through the representation of the first (Comp 1) and second components (Comp 2) of the final partial least-squares discriminant analysis (PLS-DA) models from the phage-treated (green) and control (pink) groups. Phage-treated group (green) received 0.1% *Salmonella* phage (10^8^ PFU/g) via feed. The control group (pink) did not receive phages.

The relative abundance of taxonomically assigned sequences at the genus level has been described in Supplementary Table S1. A partial least-squares discriminant analysis (PLS-DA) with additive log-ratio (ALR)-transformed variables was used to elucidate the influence of phage administration on caecal microbial variations in *Salmonella*-infected broilers at the genera level. The analysis identified 17 relevant variables (at the genus level) in the final model. The classification performance of this model was 98.76% for phage-treated and 99.85% for control animals (Figure 2). The results show that these 17 genera enabled a great classification and prediction performance between the *Salmonella*-infected broilers and the control animals.

**Figure 3:**
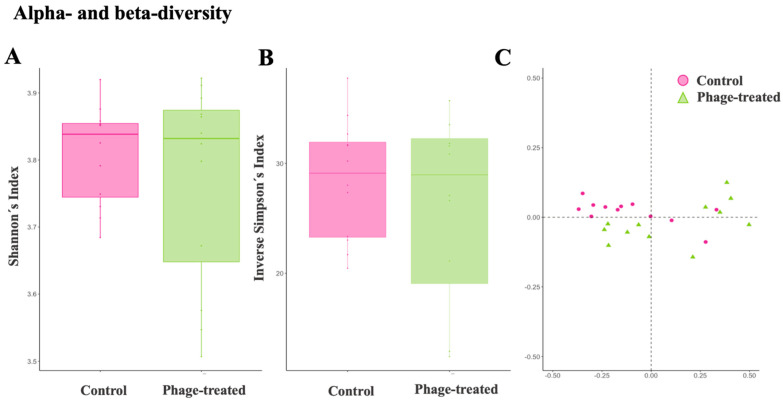
Examining the effects of *Salmonella* phage on the caecal microbiota in *Salmonella*-infected broilers. Caecal microbiota composition dissimilarity through the representation of the alpha and beta diversity scores from control and phage-treated groups. The alpha and beta diversity scores were calculated with the ALR of each species’ abundance according to a reference genus (*Family_XIII_AD3011_group*). Alpha diversity was computed using the (**A**) Shannon diversity index and (**B**) inverse Simpson index. Beta diversity was computed by calculating (**C**) the Bray–Curtis dissimilarity matrix. Differences among populations were established as having a *p* value lower than 0.05. Phage-treated group (green) received 0.1% *Salmonella* phage (10^8^ PFU/g) via feed. The control group (pink) did not receive phages.

The Shannon diversity index, which is more sensitive to species richness [29], and the inverse Simpson index, which is more sensitive to species evenness [29], showed that there were no significant differences between the phage-treated and control groups in terms of alpha diversity (Kruskal–Wallis test, Shannon diversity index: *p* value = 0.20, inverse Simpson index: *p* value = 0.22; Figure 3A,B). Moreover, in pairwise PERMANOVA comparisons between groups using Bray–Curtis, there were no significant differences between groups in terms of microbiota composition (*p* value = 0.09; Figure 3C). These results showed that, in general, both populations have a similar microbiota composition.

To better understand the effect of phage application on the *Salmonella*-infected caecal microbiota, a Bayesian statistical analysis was performed from the initial relevant genera identified using PLS-DA. As seen in Table 1, the Bayesian results showed that 14 of the 17 genera included in the final PLS-DA model display relevant differences in mean abundance between groups (Supplementary Table S2), supporting their selection by the PLS-DA model as classifier and predictive variables. Among them, *Streptococcus*, *Paludicola*, *Romboutsia*, *Hydrogenoanaerobacterium*, *UCG005*, *Weissella*, *Frisingicoccus*, *Marvinbryantia*, *Turicibacter* and *Family_XIII_UCG001* from the Firmicutes phylum and *Bacteroides* from the Bacteroidota phylum were more predominant in the phage-treated group. Meanwhile, *Faecalibacterium*, *Monoglobus* and *Erysipelatoclostridium* from the Firmicutes phylum were less predominant in the phage-treated group.

### 2.3. Effects of Phage on Gut Metabolome

An untargeted LC–MS-based metabolomics platform was used to analyse the metabolic regulation in phage-treated *Salmonella*-infected broilers, and a total of 1112 metabolites were retained.

**Figure 4:**
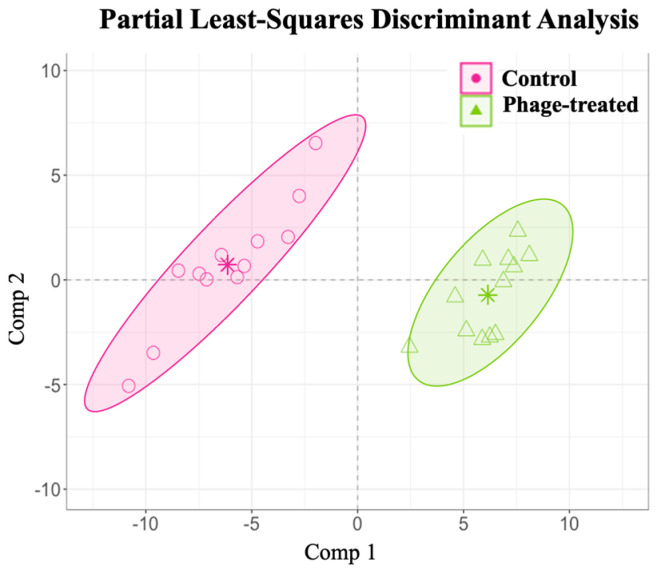
Examining the effects of *Salmonella* phage on the caecal metabolome in *Salmonella*-infected broilers. Caecal metabolome composition dissimilarity through the representation of the first (Comp 1) and second components (Comp 2) of the final partial least-squares discriminant analysis (PLS-DA) models from the phage-treated (green) and control (pink) groups. Phage-treated group (green) received 0.1% *Salmonella* phage (10^8^ PFU/g) via feed. The control group (pink) did not receive phages.

The relative abundance of the identified caecal metabolites has been described in Supplementary Table S3. A PLS-DA with ALR-transformed variables was used to elucidate the phage administration’s influence on the caecal metabolome variations in the *Salmonella*-infected broilers. Overall, the analysis identified 118 relevant variables (metabolites) in the final model for caecal samples following phage administration with a classification performance of 100% for the control and 99.92% for the phage-treated group (Figure 4).

We further verified the relevant metabolites identified with the final PLS-DA model using Bayesian statistical analysis. The Bayesian statistical analysis showed that 100 caecal metabolites from the initial 118 showed relevant mean differences between the groups (Supplementary Table S4). From these 100 relevant metabolites, 63 were *up-accumulated* and 37 were *down-accumulated* compared to the control group. From all these 100 relevant metabolites, 21 could be tentatively identified. The structures of the identified metabolites included lipids and lipid-like molecules (12), organic acids and derivates (2), organic oxygen compounds (2), phenylpropanoids and polyketides (2), organoheterocyclic compounds (1), benzenoids (1) and organic nitrogen compounds (1) (Table 2). From all these 100 relevant metabolites, the non-identified metabolites have been described in Supplementary Table S5.

**Figure 5:**
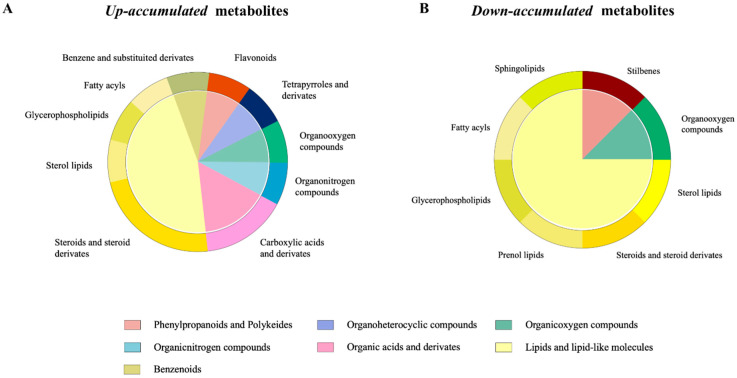
Examining the effects of *Salmonella* phage on the caecal metabolome in *Salmonella*-infected broilers. Class (inside of the cycle) and subclass (outside of the cycle) of significant (**A**) *up-accumulated* and (**B**) *down-accumulated* metabolites in phage-treated group identified with partial least-squares discriminant analysis (PLS-DA) for discriminating between groups with relevant differences in mean abundance based on Bayesian statistical analysis in phage-treated broilers compared with the control group, computed as phage-treated vs. control. Phage-treated group received 0.1% *Salmonella* phage (10^8^ PFU/g) via feed. The control group did not receive phages.

Regarding the 63 metabolites that were probably *up-accumulated* in the phage-treated group, the structure of the identified metabolites is shown in Figure 5A and corresponds mainly to steroids and steroid derivates (lipids and lipid-like molecules) and carboxylic acids (organic acids and derivates). Regarding the 37 metabolites that were *down-accumulated* in the phage-treated group, the structure of the identified metabolites is shown in Figure 5B and corresponds to lipids and lipid-like molecules, organic oxygen compounds and phenylpropanoids and polyketides.

### 2.4. Effects of Phage on Serum Metabolome

In the case of serum metabolomics changes, a total of 612 metabolites were detected.

**Figure 6:**
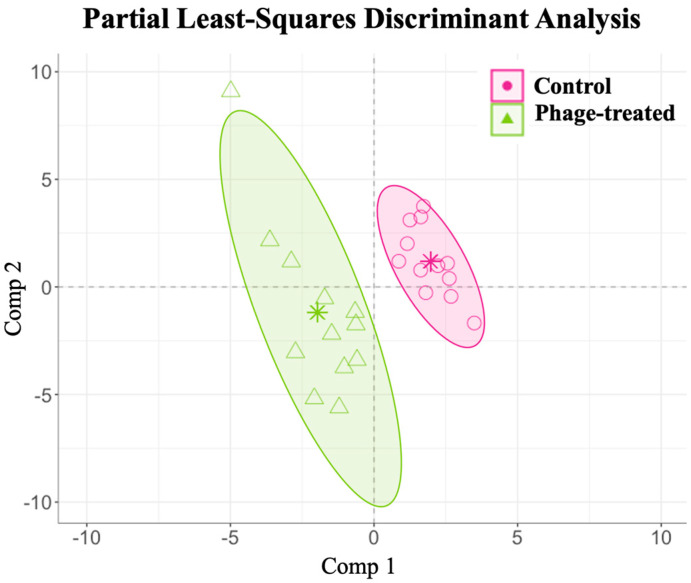
Examining the effects of *Salmonella* phage on the serum metabolome in *Salmonella*-infected broilers. Serum metabolome composition dissimilarity through the representation of the first (Comp 1) and second components (Comp 2) of the final partial least-squares discriminant analysis (PLS-DA) models from the phage-treated (green) and control (pink) groups. Phage-treated group (green) received 0.1% *Salmonella* phage (10^8^ PFU/g). The control group (pink) did not receive phages.

The relative abundance of the identified serum metabolites has been described in Supplementary Table S6. Similar to the caecal samples, a PLS-DA with ALR-transformed variables was also used to elucidate the phage administration’s influence on the serum metabolome variations in the *Salmonella*-infected broilers. The analysis identified 45 relevant variables (metabolites) in the final model for the serum samples following phage administration with a classification performance of 99.66% for the control and 99.26% for the phage-treated group (Figure 6).

We further verified the relevant metabolites identified with the final PLS-DA model using Bayesian statistical analysis. The Bayesian statistical analysis showed that 16 serum metabolites from the initial 45 showed relevant mean differences between the groups (Supplementary Table S7). For these 16 significant metabolites, 4 were *up-accumulated* and 12 were *down-accumulated* compared to the control group. Of these, 8 could be tentatively identified. The structures of the identified metabolites included organoheterocyclic compounds (3), lipid and lipid-like molecules (2), organic acids and derivates (1), organic oxygen compounds (1) and phenylpropanoids and polyketides (1) (Table 3).

**Figure 7:**
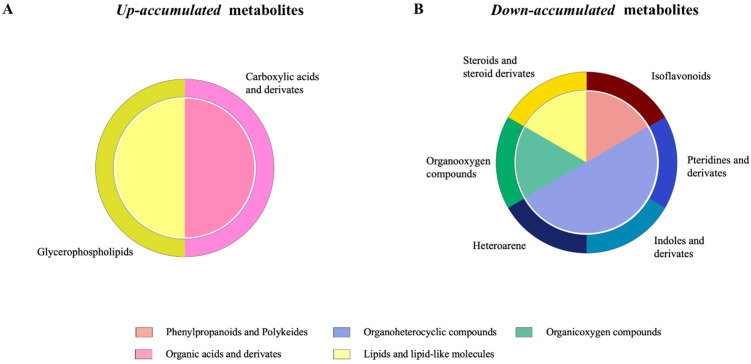
Examining the effects of *Salmonella* phage on the serum metabolome in *Salmonella*-infected broilers. Class (inside of the cycle) and subclass (outside of the cycle) of significant (**A**) *up-accumulated* and (**B**) *down-accumulated* metabolites in phage-treated group identified with partial least-squares discriminant analysis (PLS-DA) for discriminating between groups with relevant differences in mean abundance based on Bayesian statistical analysis in phage-treated broilers compared with the control group, computed as phage-treated vs. control. Phage-treated group received 0.1% *Salmonella* phage (10^8^ PFU/g) via feed. The control group did not receive phages.

For the four metabolites that were probably *up-accumulated* in the phage-treated group according to Bayesian statistical significance, the structure of the identified metabolites is shown in Figure 7A and corresponds to glycerophospholipids such as glycerophosphoethanolamines (lipids and lipid-like molecules) and carboxylic acids and derivates such as amino acids, peptides and analogues (organic acids and derivate compounds). For the 12 metabolites that were *down-accumulated* in the phage-treated group according to Bayesian statistical significance, the structure of the identified metabolites is shown in Figure 7B and corresponds mainly to pteridines and derivates, indoles and derivates such as tryptamines and heteroarene derivates such as polycyclic heteroarene (organoheterocyclic compounds).

## 3. Discussion

The therapeutic potential of bacteriophages to support *Salmonella* control in poultry flocks has been demonstrated over the last years [30,31,32,33,34,35]. Nevertheless, it has been increasingly established that phages influence host physiology through microbiota modulation by depleting bacterial species important for homeostasis [36]. However, studies to detect changes in the gut microbiome in infected (bacterial) and treated (phage) or uninfected and treated animals have yielded contradictory results on the non-targeted bacteria [11,37,38,39,40,41]. Our study provides evidence that *Salmonella* phage modulates the caecal microbiota and metabolome but has no effect on the body weight and minimal influence on the blood serum metabolome, suggesting that phage treatment may indeed have no biological significance in broilers.

Although phage therapy seems to be safe and well-tolerated in mammals, a complete understanding of phage–host interactions is lacking [42]. The main theoretical advantage of phages over antibiotics is that they do not affect the gut microbial community [11]. Nevertheless, several studies have demonstrated that phages induce changes in the microbiome, although these do not appear to be of biological significance [11,37,43,44,45,46]. In this study, the phage group had altered microbiome and metabolome profiles compared to the non-treated group. Still, the treatment for the phage group did not affect the number of species found (alpha diversity) or the number of unique species (beta diversity), in agreement with previous studies [10]. Specifically, we found changes in the relative abundance of a few genera using the PLS-DA and Bayesian approaches. However, our findings suggest that subtle changes at the genus level are accompanied by substantial changes in caecal metabolites. These changes in metabolic profile based on gut microbiota concur with previous research comparing non-treated and treated *Salmonella*-infected broilers with different antimicrobials [47]. Caecal alterations presented a context-specific singularity that entails changes in the interaction between microbes (probably with altered abundance) and host epithelial/immune cells, leading to alterations in the shedding of microbial-associated molecular patterns in the gut and in the availability of the metabolites produced by the gut microbiota [48]. Still, we do not know whether these metabolic changes reflect a direct involvement of phages in the central microbiome or are the result of altering the relative abundance of the identified genera. Further studies are needed to elucidate this issue.

We found that most of the altered genera in the phage-treated group presented an increase in bacterial abundance. For instance, the abundance of *Bacteroides* was significantly enhanced. In previous studies, this beneficial bacterial genus has been found in *Salmonella*-infected chickens treated with probiotics [49,50]. This genus has been related to acetic acid production and its influence on lipid metabolism [51]. Lipids regulate biological processes such as immunity and inflammation [52]. In addition, *Bacteroides* has been associated with the metabolism of bile acids, proteins, fats and carbohydrates [51]. These observations may be consistent with the alteration of the caecal metabolites observed in this study. It is noteworthy to mention that the level of bile acids in the gut can affect microbial community abundance [52]. Moreover, bile acids are related to the regulation of hepatic metabolic pathways [52], which have a protective effect against sepsis via different mechanisms such as bacterial clearance and adaptation to inflammation [52,53]. After treatment, the *Romboutsia* genus has been identified in *Salmonella*-infected laying hens [50]. This genus has been described as part of the commensal bacteria involved in carbohydrate utilisation, simple amino acid fermentation and anaerobic respiration [54]. Additionally, the *Weissella* genus from the *Leuconostocaceae* family and the *Turicibacter* genus from the *Erysipelotrichaceae* family have previously been described in the chicken and mammalian gut [50,55]. We also identified an increase in the abundance of the *Weissella* genus after *Salmonella* treatment, similar to previous studies in layers [50]. The *Weissella* genus has been described as a *Salmonella* antimicrobial [56]. *Weissella* are heterofermentative lactic acid bacteria that are part of the autochthonous microbiota that helps in host health-status maintenance and gut homeostasis [54,57]. Meanwhile, *Turicibacter* has been related to subclinical infections in the mammalian gut and colitis but has also been considered a healthy genus [58,59]. As for the genus *Hydrogenoanaerobacterium* from the *Oscillospirales* family, a sugar-fermenting and hydrogen-producer, it has been positively correlated with body weight [60,61]. Low levels of *Oscillospirales* in patients have been described as associated with dysbiosis [62]. Finally, *Family_XIII_UCG001* from the *Anaerovoracaceae* family also presented higher levels in the phage group. This family function in the gut was hitherto unknown; however, it belongs to the class of *Clostridia*, which is involved in the fermentation of plant polysaccharides [63]. It is worth mentioning that a highly relative abundance of *Lachnospiraceae* (*Frisingicoccus* and *Marvinbryantia* genus) and *Ruminococcaceae* (*Paludicola* genus) families has been observed in the caecal microbiota composition in *Salmonella*-infected chickens [64]. Moreover, members of these families are considered producers of butyric acid and short-chain fatty acids through carbohydrate fermentation which presents a potential protective role in *Salmonella* colonisation resistance in the gut [64]. Note that fatty acids have been related to reducing *Salmonella* virulence through the restriction of host invasion, the maintenance of the gut barrier integrity and intestinal immunity activation [48,65]. Admittedly, most of the significantly different fatty acids identified in our study were *up-accumulated* in the phage group.

On the other hand, a decrease in *Ruminococcaceae* has been associated with an increase in *Salmonella* colonisation susceptibility [66]. Genera *Faecalibacterium*, *Monoglobus* and *Erysipelatoclostridium* were decreased in the phage group, with the first two considered commensals in the chicken caecum whose role has been linked to pectin degradation [67,68,69]. Moreover, *Faecalibacterium* was identified as a butyrate-producing genus with anti-inflammatory properties due to its regulation of inflammatory gene expressions and apoptosis in host-cell degradation [67,68,69]. Regarding the genus *Erysipelatoclostridium*, it has been suggested that it interacts positively to displace *Salmonella* in the poultry gut microbiota [50].

The underlying question derived from our results is whether perturbations of the caecal microbiome and metabolome confer phenotypic alterations. Although it is known that the gut microbiota plays an essential role in health, and this fact has been receiving increasing attention in recent years, the specific role of bacteria is currently unknown, partly because of the complexity of bacterial interactions [36]. Admittedly, the gut microbiota and its metabolic activities have essential effects on chickens’ health status and performance [70,71,72,73]. Likewise, it has been reported that phages can modulate bacterial communities, but phages also influence the gut ecosystem by interacting directly with the immune cells, thereby modulating host immune activity [74,75,76]. Moreover, the phage can cross the epithelial barrier through a process known as transcytosis [77] and interact directly with the immune cells. Because blood serum profiles reflect changes in the host’s metabolism rather than in the gut microbial activity [78], our comparison between the blood serum metabolome of the phage-treated and untreated groups showed a high similarity in the metabolic profile. For example, the phage group exhibited high glycerophospholipid levels. These metabolites have been considered antimicrobial and immunomodulatory in broilers [52], which would be expected after the phage treatment. In addition, lower levels of tryptamines and derivates (5-Methoxytryptophan) were noted in the phage group. Note that high levels of tryptamine derivatives have been described in inflammatory gut diseases [79]. Previous studies have already shown alterations in polyketides in broilers after treatment with prebiotics [80]. Polyketides are considered secondary metabolites characterized by different sources, including microorganisms [81]. Polyketides are synthetized by polyketide synthases. This enzyme can be divided into three types, and Types I and II occur in bacteria [81]. Thus, if changes in the microbiota are occurring, even if they are subtle as in the case of phage application, it is possible to observe changes in polyketides. Overall, the magnitude of the changes in the blood serum of the phage-treated group appears not to have caused a significant physiological response. As such, these results are confirmed by the observation that the administration of phages did not influence the chicks’ performance from the early to later growth stages (with or without bacterial target challenge), which is consistent with several previous studies [32,41,82,83,84]. The translational value of these findings to other production systems, such as laying hens or other species, could potentially be a source of bias due to the short period of rearing in broilers (6 weeks old). Therefore, further long-term studies are required to assess the sustained effects and investigate the role of phages in the immune response.

## 4. Materials and Methods

### 4.1. Caecal and Serum Content Origin

**Figure 8:**
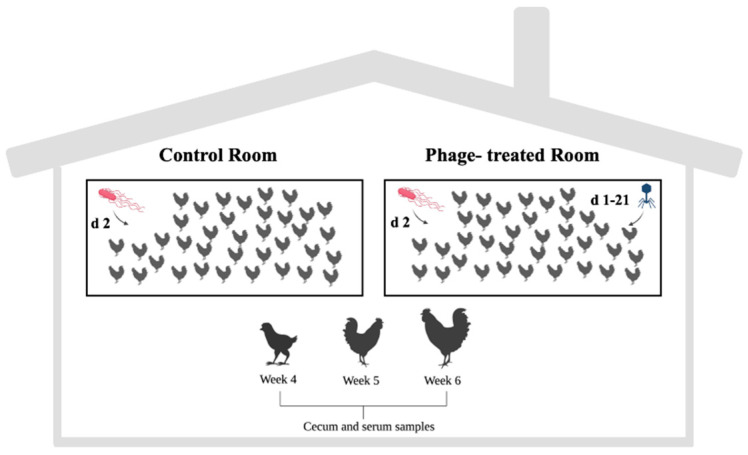
Experimental design of the study. Half of the animals in both groups were challenged with *Salmonella* Enteritidis on the second day of the rearing period. Phage-treated group received 0.1% encapsulated *Salmonella* phage (10^8^ PFU/g) with feed (days 1 to 21 of the rearing period). The control group did not receive phages.

Twelve caecal and serum samples stored at −80 °C were thawed to carry out this study. The samples were obtained from a prior experiment that involved the application of phages for *Salmonella* control during the broiler rearing period [18]. Briefly, a total of 100 one-day-old *Salmonella*-free male Ross broilers were randomly divided into two treatment groups of 50 animals each (phage-treated and non-phage (control)). Twenty-four hours after arriving, 50% of the chicks from each experimental group were orally infected with *Salmonella enterica* ser. Enteritidis (10^5^ CFU/bird). For the first 21 days, the birds from the phage-treated group were fed with 0.1% encapsulated *Salmonella* phages (10^8^ PFU/g). The *Salmonella* phage used in this study was isolated by Sevilla-Navarro et al. [85] and characterized by Lorenzo-Rebenaque et al. [86]. The phages were micro-encapsulated with the polymer Eudragit^®^ L100 (L100) using the spray-dry technique [86,87]. The birds in the control group were fed without phages throughout the rearing period (Figure 8). Blood samples and caecal content samples were collected from 4 chickens at weekly intervals during the rearing period. In addition, the body weight data of the animals were collected.

Approximately 2 mL of blood was centrifuged at 4000× *g* for 15 min, and the serum was introduced in liquid nitrogen and preserved at −80 °C for metabolome analysis. The caecal content was collected and aliquoted into two parts and immediately snap-freezed with liquid nitrogen and kept frozen at −80 °C for DNA and metabolome extraction. For analysis of the microbiota and metabolome, samples corresponding to weeks 4 to 6 were chosen according to the microbiota maturation and stabilisation [11,41]. This behaviour is consistent with previous reports where the chicken microbiome shows a rapid increase in microbial diversity until approximately the third week when it stabilizes [88].

### 4.2. Microbiota Analysis

#### 4.2.1. DNA Extraction, 16S rRNA Gene Amplification and MiSeq Sequencing

The DNA was extracted from 250 mg of each homogenised caecal content following the manufacturer’s instructions (QIAamp Power Fecal Pro DNA kit, Werfen, Barcelona, Spain). The DNA quality was determined using Nanodrop ND-1000 spectrophotometer (Thermo Scientific, Wilmington, DE, USA), and DNA quantity was determined using Qubit fluorometer (Life Technologies, Paisley, UK). The DNA was frozen at −20 °C for shipment to the *Instituto de Investigación Sanitaria y Biomédica de Alicante—ISABIAL* (Alicante, Spain). The 16S rRNA gene amplicon libraries were prepared using the 16S Metagenomic Sequencing Library Preparation, Preparing 16S Ribosomal RNA Gene Amplicons for the Illumina MiSeq System (Illumina^®^, San Diego, CA, USA) protocol. Primer sequences cover the V3–V4 regions of the 16S rRNA gene [89]. The following primers also include the Illumina adapters: 16S Amplicon PCR Forward Primer = 5′ TCGTCGGCAGCGTCAGATGTGTATAAGAGACAGCCTACGGGNGGCWGCAG; and 16S Amplicon PCR Reverse Primer = 5′ GTCTCGTGGGCTCGGAGATGTGTATAAGAGACAGGACTACHVGGGTATCTAATC. The MiSeq (Illumina) system in 2 × 300 bp format sequencing was performed to sequence the Illumina libraries. FastQC software (v. 1.0.0) was used to evaluate the quality of the raw, unprocessed reads [90].

#### 4.2.2. Bioinformatic Analysis

The 16S rRNA gene amplicon sequencing results are available at NCBI (BioProject PRJNA880003). Raw sequencing data were processed using QIIME2 v2021.4. The DADA2 pipeline incorporated into QIIME2 was used to complete the denoising, filtering and chimera removal of the sequences, and reads were clustered in Amplicon Sequence Variants (ASVs). Depending on the sequence quality (quality-score acceptance rate of 20 or better), forward and reverse reads were trimmed to 285 and 216 bp, respectively. The primer sequences were removed from all reads. Taxonomy was assigned to the obtained ASVs to the genus level, and the ASV abundance estimates were determined using SILVA v138 database taxonomic training data formatted for DADA2 (99% 16S full-length) [91,92]. Reads not assigned to any taxa or classified as Eukaryote or Archaea or found in less than 20% of the samples in both groups were removed from the analysis. Sequencing statistical analyses were performed using QIIME2 v2021.4.

### 4.3. Metabolomics Analysis

#### 4.3.1. Sample Preparations

The caecal metabolites were extracted from 10 mg of each homogenised caecal content following a published method with a little modification [93]. Briefly, samples were dissolved in cold aqueous methanol (75 μL, 75%) and formic acid (0.1%), spiked with 10 μg/mL formononetin as internal standard. Then, the mix was shaken for 40′ at 20 Hz using a Mixer Mill 300 (Qiagen, Barcelona, Spain). After centrifugation at 20,000× *g* for 15 min at 4 °C, 600 μL of the supernatant was taken and transferred to a new 2 mL conical tube. The supernatants were transferred to HPLC filter tubes (0.22 µm pore size, Whatmann^TM^), and an aliquot of 3 μL of each sample was injected for the analysis. For LC–ESI–HRMS analysis, LTQ-Orbitrap Discovery was used as mass spectrometry system (Thermo Fisher Scientific) as previously described [41,94].

The serum metabolites were extracted from 100 μL of each serum sample following a published method with slight modification [95]. Briefly, samples were dissolved in cold aqueous methanol (200 μL, 75%) and acetonitrile (200 μL, 75%), spiked with 10 μg/mL formononetin as internal standard. After centrifugation at 20,000× *g* for 15 min at 4 °C, 200 μL of the supernatants were taken and dried under low-temperature vacuum (Thermo Scientific, USA). The samples were redissolved, resuspended with 100 μL of methanol (10%) and transferred to HPLC tubes, and an aliquot of 3 μL was injected for the analysis.

#### 4.3.2. LC–ESI–HRMS Analysis

Untargeted LC–ESI–HRMS analyses of the caecum and serum samples were conducted as reported before [94] at the *Agenzia nazionale per le nuove tecnologie, l’energia e lo sviluppo economico sostenibile* (ENEA, Roma, Italy). The data were further processed with Compound Discoverer software (v. 3.0, Thermofisher Scientific, Barcelona, Spain). After detection of the features (the m/z and rt for each peak) and chromatogram alignment, the data generated were normalised with respect to the internal standard. After chromatogram alignment and retrieval of all the detected frames (e.g., ions), the data generated were normalised with respect to the internal standard. These data are available at the NIH Common Fund’s National Metabolomics Data Repository (NMDR) website, the Metabolomics Workbench, https://www.metabolomicsworkbench.org (accessed 11 October 2023), where it has been assigned Study ID ST002311 for caeca metabolites and Study ID ST002312 for serum metabolites. The data can be accessed directly via their project DOI: 10.21228/M8598K.

For metabolite identification, a manual curation using the Metlin database was performed (https://metlin.scripps.edu/) (accessed 10 September 2022). Tentative identifications were validated by comparing chromatographic and spectral properties with authentic standards (when available) and reference spectra, in-house database and literature data and based on the m/z accurate masses as reported in the PubChem database (http://pubchem.ncbi.nlm.nih.gov/) (accessed 10 September 2022) for monoisotopic mass identification, subsequently confirmed with MS/MS fragmentation.

### 4.4. Statistical Analysis

#### 4.4.1. Body Weight Statistical Analysis

A Generalised Linear Model (GLM) was used to evaluate the differences due to phage treatment on the body weight. The experimental group (phage-treated vs. control group) was included as a fixed effect. Statistical differences were based on a *p* value level < 0.05. Statistical analyses were performed using SPSS 27.0 software package (SPSS Inc., Chicago, IL, USA).

#### 4.4.2. Caecal Microbiome and Caecal and Serum Metabolome Statistical Analysis

Statistical analysis of caecal microbiome and caecal and serum metabolome composition was performed following the same methodology. No outlier samples were identified using a principal component analysis with the dataset without zeros, so all samples remained in the datasets. Genera and metabolites with almost 20% zeros within each treatment were removed [96]. The remaining zeros were replaced by one for microbiome data and by half of the minimum value detected for each metabolite. A total of 110 genera, 1112 caecal metabolites and 612 serum metabolites from 24 samples remained in the datasets. Datasets were transformed using the additive log-ratio (ALR) transformation as follows:

(1) $$ALR\left(j|ref\right)=log\left(\frac{x_{j}}{x_{ref}}\right)=\log\left(x_{j}\right)-\log\left(x_{ref}\right),$$

where j is the total number of variables in the dataset, $x_{j}$ is the value for the genera or metabolite j and $x_{ref}$ is the reference variable used to transform the data. The reference variable for metabolome data was a standard chemical (formononetin) injected into the platform and run at a fixed concentration. For microbiome data, the reference variable was the one with the lowest coefficient of variation ($x_{ref}$; *Family_XIII_AD3011_group*). The lack of isometry was checked using Procrustes correlation [97]. ALRs were autoscaled with mean of 0 and standard deviation of 1.

A partial least-squares discriminant analysis (PLS-DA) was used to identify the genera and metabolites that allowed us to classify or discriminate among the treatments. PLS-DA models were computed with the mixOmics package in R [98] using the treatments as the categorical vector *y* and the ALR dataset for genera or metabolites as the matrix *X*. The balance error rate (BER) for the Mahalanobis distance, computed using a 4-fold cross-validation repeated 100 times, was used to select the optimal number of components of the model in each iteration process. In each iteration, variables with a variable importance prediction (VIP) lower than 1 were removed from the X matrix because they were not informative for the classification among the treatments [99]. After variable selection, a new PLS-DA model was computed. Variable selection and PLS-DA model computation were conducted until the lowest BER was achieved. The prediction performance of the final PLS-DA model was checked with the construction of a confusion matrix and a permuted-confusion matrix using a 4-fold cross-validation repeated 10,000 times. The former allows us to determine the ability of the model to predict each treatment according to the variables selected by the PLS-DA. The latter determines whether the performance achieved is due to a spurious selection of variables throughout the PLS-DA iterations. The prediction performance was considered spurious when the percentage of true positives for each treatment was far from their random probabilities (33% for three categories and 50% for two categories).

Bayesian statistics were used in addition to PLS-DA to measure the relevance of the differences in the genera and metabolite abundance between the control and treatment groups. A model with a single effect of “treatment” and flat priors was fitted. Estimation of the marginal posterior distribution of the unknowns was conducted with MCMC using four chains of 50,000 iterations, with a burn-in of 1000 and a lag of 10. The mean of the marginal posterior distribution of the differences between the control and each of the two types of phage administration was used to estimate the posterior mean of the differences in genera or metabolites between the control and the treatment groups. These estimates were reported as the unit of standard deviation (SD) of each variable. The differences in the mean abundance of the genera and metabolites between the control and the treatment groups were considered relevant when these differences were higher than 0.5 units of SD and when the probability of the differences [100] being greater (if the difference is positive) or lower (if negative) than 0 (P0) was higher than 0.9.

The alpha and beta diversity were computed using the ALR at the species level to measure the differences in microbiome composition among groups. The alpha diversity was measured using the Shannon diversity and inverse Simpson indexes to analyse the species diversity and evenness. Differences in the distribution of alpha diversity among groups were considered when the *p* value of a Mann–Whitney U test was lower than 0.05. Beta diversity was measured using the Bray–Curtis dissimilarity matrix, and a nonmetric multidimensional scaling (NMDS) was carried out to retrieve the loadings of the first two dimensions. Differences in microbial genera composition were tested using a permutational multivariate analysis of variance (PERMANOVA; *p* value  <  0.05) on the loadings of the two first MDS dimensions.

## 5. Conclusions

In summary, the current study demonstrates that the application of *Salmonella* phages under production conditions alters the caecal microbiome and metabolome profiles of broilers. However, the lack of significant changes in blood serum metabolites or growth performance indicates that this modulation by phages may not have biological significance. Future studies are needed to determine whether the observed shift in the microbiota composition, which drives the change in the metabolic profile, is merely a result of the *Salmonella* phages altering the microbiota or whether the phages themselves actively contributed to the metabolite change.

## Figures and Tables

**Table 1:** Examining the effects of *Salmonella* phage on the caecal microbiota in *Salmonella*-infected broilers. Key genera identified using partial least-squares discriminant analysis (PLS-DA) for discriminating between groups with relevant differences in mean abundance based on Bayesian statistical analysis in phage-treated broilers compared with the control group, computed as phage-treated vs. control. Phage-treated group received a 0.1% *Salmonella* phage (10^8^ PFU/g) via feed. The control group did not receive phages.

Phylum	Family	Genus	HPD95	P0	D
Firmicutes	*Streptococcaceae*	*Streptococcus*	[−0.03, 1.59]	96.71	0.76
	*Staphylococcaceae*	*Faecalibacterium*	[−1.51, 0.15]	94.89	−0.68
	*Ruminococcaceae*	*Paludicola*	[−0.2, 1.47]	92.69	0.61
	*Peptostreptococcaceae*	*Romboutsia*	[0.11, 1.68]	98.62	0.90
	*Oscillospirales*	*Hydrogenoanaerobacterium*	[0.04, 1.65]	97.99	0.84
	*Oscillospiraceae*	*UCG005*	[0.66, 1.97]	99.98	1.31
	*Monoglobaceae*	*Monoglobus*	[−1.49, 0.16]	94.64	−0.67
	*Leuconostocaceae*	*Weissella*	[−0.18, 1.5]	92.95	0.62
	*Lachnospiraceae*	*Frisingicoccus*	[0.63, 1.95]	99.97	1.29
		*Marvinbryantia*	[0.55, 1.94]	99.89	1.22
	*Erysipelotrichaceae*	*Turicibacter*	[0.77, 2.01]	99.99	1.40
	*Erysipelatoclostridiaceae*	*Erysipelatoclostridium*	[−1.65, −0.09]	98.64	−0.89
	*Anaerovoracaceae*	*Family_XIII_UCG001*	[−0.25, 1.43]	92.26	0.60
Bacteroidota	*Bacteroidaceae*	*Bacteroides*	[0.7, 1.98]	99.99	1.33

HPD95 = The highest posterior density region at 95% probability. P0 = Probability of the difference (Dphage-control) being greater than 0 when Dphage-control > 0 or lower than 0 when Dphage-control < 0. Dphage-control = Mean of the difference − phage-treated control (median of the marginal posterior distribution of the difference between the control group and phage-treated group). Statistical differences were assumed if |Dphage-control| surpassed R value and its P0 > 0.90.

**Table 2:** Examining the effects of *Salmonella* phage on the caecal metabolome in *Salmonella*-infected broilers. Key metabolites identified using partial least-squares discriminant analysis (PLS-DA) for discriminating between groups with relevant differences in mean abundance based on Bayesian statistical analysis in phage-treated broilers compared with the control group, computed as phage-treated vs. control. Phage-treated group received a 0.1% *Salmonella* phage (10^8^ PFU/g) via feed. The control group did not receive phages.

Superclass	Class	Subclass	Metabolite	HPD95 _Phage-Control_	P0 _Phage-Control_	D _Phage-Control_
Benzenoids	Benzene and substituted derivates	Phenyl methyl carbamates	2-(Ethylsulfonylmethyl)phenyl methyl carbamate-like	[0.3, 1.8]	99.47	1.03
Lipids and lipid-like molecules	Fatty acyls	Eicosanoids	9-deoxy-9-methylene-PGE2	[−1.43, 0.27]	92.19	−0.60
		Fatty alcohols	Persenone A-like	[0.54, 1.93]	99.88	1.21
		Stigmasterols and C24-ethyl derivatives	5alpha,8alpha-epidioxy-stigmasta-6,9(11),22E-trien-3beta-ol-like	[−1.73, −0.18]	98.92	−0.93
		Cholesterol and derivates	9,11alpha-epoxy-6alpha-acetoxy-cholest-7-en-3beta,5alpha,19-triol	[0.37, 1.82]	99.80	1.11
	Steroids and steroid derivates	Steroid ester	Estra-1,3,5(10)-triene-3,6beta,17beta-triol triacetate	[0.53, 1.91]	99.93	1.22
		Sulfate steroids	Pregnanolone sulfate	[−1.82, −0.35]	99.70	−1.08
		Bile acids, alcohols and derivates	Perulactone	[1.05, 2.11]	100.00	1.57
		Stigmastane and derivates	7-Oxostigmasterol-like	[0.82, 2.03]	100.00	1.42
	Sphingolipids	Phosphosphingolipids	SM(d18:1/0:0)	[−1.82, −0.35]	99.69	−1.08
	Prenol lipids	Isoprenoids	(+)-3beta-Hydroxy-ursan-28-oic acid-like	[−1.71, −0.14]	98.85	−0.92
	Glycerophospholipids	Glycerophosphoethanolamines	PE(14:0/0:0)	[0.37, 1.81]	99.80	1.09
			PC(18:2(2E,4E)/0:0)	[−1.62, −0.03]	97.83	−0.83
Organic acids and derivates	Carboxylic acids and derivates	Amino acids, peptides and analogues	Yersiniabactin	[1.73, 2.12]	100.00	1.91
		Monocarboxylic acid	1-(3,4-Dihydroxyphenyl)-1-decene-3,5-dione	[−1.11, 0.6]	72.98	−0.26
		Carboxylic acid derivates	(S,E)-Lyratol propanoate	[0.68, 1.98]	99.98	1.33
Organic nitrogen compounds	Organonitrogen compounds	Organic nitrous compounds	3-[(3-Methylbutyl)nitrosamine]-2-butanone	[0.82, 2.03]	99.99	1.43
Organic oxygen compounds	Organooxygen compounds	Carbohydrates and carbohydrate conjugates	D-Glucosamine 1-phosphate	[0.59, 1.94]	99.97	1.28
		Carbohydrates and carbohydrate conjugates	Glucosyl (E)-2,6-Dimethyl-2,5-heptadienoate	[−1.88, −0.46]	99.90	−1.18
Organoheterocyclic compounds	Tetrapyrroles and derivates	Bilirubins	Mesobilirubinogen	[0.18, 1.71]	99.22	0.95
Phenylpropanoids and polyketides	Stilbenes	Stilbenes	Batatasin III-like	[−1.84, −0.37]	99.73	−1.09
	Flavonoids	Flavans	Kaempferol 7,4′-dimethyl ether 3-(6″-(E)-p-coumarylglucoside)-like	[1.27, 2.15]	100.00	1.71

HPD95 phage-control = The highest posterior density region at 95% probability. P0 = Probability of the difference (D phage-control) being greater than 0 when D phage-control > 0 or lower than 0 when D phage-control < 0. D phage-control = Mean of the difference − phage-treated control (median of the marginal posterior distribution of the difference between the control group and phage-treated group). Statistical differences were assumed if |D phage-control| surpassed R value and its P0 > 0.90.

**Table 3:** Examining the effects of *Salmonella* phage on the serum metabolome in *Salmonella*-infected broilers. Key metabolites identified with partial least-squares discriminant analysis (PLS-DA) for discriminating between groups with relevant differences in mean abundance based on Bayesian statistical analysis in phage-treated broilers compared with the control group, computed as phage-treated vs. control. Phage-treated group received 0.1% *Salmonella* phage (10^8^ PFU/g) via feed. The control group did not receive phages.

Superclass	Class	Subclass	Metabolite	HPD95 _Phage-Control_	P0 _Phage-Control_	D _Phage-Control_
Organoheterocyclic compounds	Indoles and derivates	Tryptamines and derivates	5-Methoxytryptophan	[−1.8, −0.3]	99.63	−1.05
	Pteridines and derivates	Pterins and derivates	6-Lactoyltetrahydropterin	[−1.63, −0.03]	97.69	−0.82
	Heteroarene	Polycyclic heteroarene	Indolylmethylthiohydroximate	[−1.88, −0.48]	99.92	−1.2
Organic oxygen compounds	Organooxygen compounds	Carbohydrates and carbohydrate conjugates	D-Mannitol	[−1.7, −0.14]	98.8	−0.92
Organic acids and derivates	Carboxylic acids and derivates	Amino acids, peptides, and analogues	L-Ornithuric acid	[0.35, 1.8]	99.69	1.08
			Prolyl-Tyrosine	[−0.61, 1.13]	69.97	0.22
Lipids and lipid-like molecules	Steroids and steroid derivates	Bile acids, alcohols and derivates	Murocholic acid-like	[−1.41, 0.27]	91.6	−0.58
	Glycerophospholipids	Glycerophosphoserines	PE(17:0/0:0)	[−0.26, 1.42]	92.76	0.61
Phenylpropanoids and polykeides	Isoflavonoids	O-methylated isoflavonoids	Homoferreirin	[−1.43, 0.27]	91.27	−0.57
Non-identified metabolite 50				[−1.7, −0.16]	98.85	−0.93
Non-identified metabolite 322				[−1.81, −0.32]	99.63	−1.06
Non-identified metabolite 323				[−1.87, −0.42]	99.81	−1.12
Non-identified metabolite 346				[−1.78, −0.29]	99.62	−1.06
Non-identified metabolite 395				[−1.81, −0.34]	99.65	−1.05
Non-identified metabolite 458				[−1.81, −0.32]	99.59	−1.05
Non-identified metabolite 461				[0.27, 1.76]	99.51	1.02
Non-identified metabolite 490				[−0.3, 1.39]	90.14	0.54

HPD95phage-control = The highest posterior density region at 95% probability. P0 = Probability of the difference (Dphage-control) being greater than 0 when Dphage-control > 0 or lower than 0 when Dphage-control < 0. Dphage-control = Mean of the difference − phage-treated control (median of the marginal posterior distribution of the difference between the control group and phage-treated group). Statistical differences were assumed if |Dphage-control| surpassed R value and its P0 > 0.90.

## Data Availability

The datasets generated for this study can be found in the NCBI’s BioProject PRJNA880003 and in the NIH Common Fund’s National Metabolomics Data Repository (NMDR) website, the Metabolomics Workbench, https://www.metabolomicsworkbench.org (accessed 11 October 2023), where it has been assigned Study ID ST002311 and ST002312.

## Supplementary Materials

The supporting information can be downloaded at: https://www.mdpi.com/article/10.3390/ijms242015201/s1.
