# Supplementary material for: Modulation of Caecal Microbiota and Metabolome Profile in Salmonella-Infected Broilers by Phage Therapy
Source: Int J Mol Sci. 2023 Oct 15;24(20):15201. doi: 10.3390/ijms242015201 (PMC10607084; doi:10.3390/ijms242015201)
Supplement: Supplementary file 1 [file ijms-24-15201-s001.zip › ijms-2649518-supplementary.pdf]

International Journal of *Molecular Sciences*

MDPI

Supplementary Material

Caecal microbiota and metabolome analysis reveals a profile modulation in a Salmonella phage therapy in *Salmonella*-infected broilers

Laura Lorenzo-Rebenaque[1], Cristina Casto-Rebollo[2], Gianfranco Diretto[3], Sarah Frusciante[3], Juan Carlos Rodríguez[4], María-Paz Ventero[5], Carmen Molina-Pardines[5], Santiago Vega[1], Clara Marin[1], Francisco Marco-Jiménez[2]

1 Department of Animal Production and Health, Veterinary Public Health and Food Science and Technology, Biomedical Research Institute, Faculty of Veterinary Medicine, Cardenal Herrera-CEU University, CEU *Universities*, Calle Santiago Ramón y Cajal 20, 45115 Alfara del Patriarca (Valencia), Spain. laura.lorenzorebenaque@uchceu.es ; svega@uchceu.es ; clara.marin@uchceu.es

2 Institute for Animal Science and Technology, Universitat Politècnica de València, 46022 Valencia, Spain. cricasre@posgrado.upv.es ; fmarco@dca.upv.es

3 Italian Agency for New Technologies, Energy and Sustainable Development (ENEA), Biotechnology Laboratory, Centro Ricerche Casaccia, Via Anguillarese, 301, 00123 Santa Maria di Galeria RM, Italy. gianfranco.diretto@enea.it ; sarah.frusciante@enea.it

4 Microbiology Department, Dr. Balmis General University Hospital; Microbiology division, Miguel Hernández University; ISABIAL, Alicante, Spain. rodriguez_juadia@gva.es

5 Microbiology Department, Dr. Balmis General University Hospital, ISABIAL, Alicante, Spain. maripazvm@gmail.com ; carmenmolinapardines@gmail.com

* Correspondence: fmarco@dca.upv.es

International Journal of *Molecular Sciences*

MDPI

Supplementary Table S1. Examining the effects of *Salmonella* phage on the caecal microbiota in *Salmonella*-infected broilers. Relative abundance (%) of taxonomically assigned sequences at genus level.

Phylum	Class	Order	Family	Genera	Control	Phage-treated
Firmicutes	*Bacilli*	__	__	__	5.56	2.20
Firmicutes	*Clostridia*	*Oscillospirales*	__	__	0.12	0.04
Firmicutes	*Clostridia*	__	__	__	0.02	0.02
Firmicutes	*Clostridia*	*Oscillospirales*	*[Clostridium]_methylpentosum_group*	*[Clostridium]_methylpentosum_group*	0.00	0.00
Firmicutes	*Clostridia*	*Oscillospirales*	*[Eubacterium]_coprostanoligenes_group*	*[Eubacterium]_coprostanoligenes_group*	1.16	0.97
Firmicutes	*Bacilli*	*Lactobacillales*	*Aerococcaceae*	*Aerococcus*	0.00	0.01
Firmicutes	*Bacilli*	*Lactobacillales*	*Aerococcaceae*	*Facklamia*	0.00	0.01
Firmicutes	*Clostridia*	*Eubacteriales*	*Anaerofustaceae*	*Anaerofustis*	0.02	0.01
Firmicutes	*Clostridia*	*Peptostreptococcales-Tissierellales*	*Anaerovoracaceae*	*Family_XIII_UCG-001*	0.03	0.07
Firmicutes	*Clostridia*	*Peptostreptococcales-Tissierellales*	*Anaerovoracaceae*	*[Eubacterium]_brachy_group*	0.02	0.03
Firmicutes	*Clostridia*	*Peptostreptococcales-Tissierellales*	*Anaerovoracaceae*	*[Eubacterium]_nodatum_group*	0.01	0.03
Firmicutes	*Clostridia*	*Peptostreptococcales-Tissierellales*	*Anaerovoracaceae*	*Family_XIII_AD3011_group*	0.01	0.01
Firmicutes	*Clostridia*	*Peptostreptococcales-Tissierellales*	*Anaerovoracaceae*	__	0.00	0.01
Firmicutes	*Bacilli*	*Bacillales*	*Bacillaceae*	*Bacillus*	1.35	0.99
Firmicutes	*Bacilli*	*Bacillales*	*Bacillaceae*	*Oceanobacillus*	0.00	0.00
Bacteroidota	*Bacteroidia*	*Bacteroidales*	*Bacteroidaceae*	*Bacteroides*	3.26	9.24
Actinobacteriota	*Actinobacteria*	*Micrococcales*	*Brevibacteriaceae*	*Brevibacterium*	0.00	0.00
Firmicutes	*Clostridia*	*Oscillospirales*	*Butyricicoccaceae*	*Butyricicoccus*	0.81	1.33
Firmicutes	*Clostridia*	*Oscillospirales*	*Butyricicoccaceae*	*UCG-009*	0.15	0.22
Firmicutes	*Clostridia*	*Christensenellales*	*Christensenellaceae*	*Christensenellaceae_R-7_group*	0.37	0.40
Firmicutes	*Clostridia*	*Christensenellales*	*Christensenellaceae*	__	0.01	0.01
Firmicutes	*Clostridia*	*Christensenellales*	*Christensenellaceae*	*uncultured*	0.01	0.01

Firmicutes	*Clostridia*	*Clostridia_UCG-014*	*Clostridia_UCG-014*	*Clostridia_UCG-014*	1.58	1.08
Firmicutes	*Clostridia*	*Clostridia_vadinBB60_group*	*Clostridia_vadinBB60_group*	*Clostridia_vadinBB60_group*	12.17	7.78
Firmicutes	*Clostridia*	*Clostridiales*	*Clostridiaceae*	*Candidatus_Arthromitus*	0.03	0.05
Proteobacteria	*Gammaproteobacteria*	*Burkholderiales*	*Comamonadaceae*	*Comamonas*	0.00	0.18
Actinobacteriota	*Actinobacteria*	*Corynebacteriales*	*Corynebacteriaceae*	*Corynebacterium*	0.04	0.07
Firmicutes	*Clostridia*	*Lachnospirales*	*Defluviitaleaceae*	*Defluviitaleaceae_UCG-011*	0.09	0.06
Actinobacteriota	*Actinobacteria*	*Micrococcales*	*Dermabacteraceae*	*Brachybacterium*	0.01	0.03
Firmicutes	*Incertae_Sedis*	*DTU014*	*DTU014*	*DTU014*	0.00	0.02
Actinobacteriota	*Coriobacteriia*	*Coriobacteriales*	*Eggerthellaceae*	*Gordonibacter*	0.01	0.01
Actinobacteriota	*Coriobacteriia*	*Coriobacteriales*	*Eggerthellaceae*	*CHKCI002*	0.00	0.00
Proteobacteria	*Gammaproteobacteria*	*Enterobacterales*	*Enterobacteriaceae*	*Escherichia-Shigella*	1.06	0.86
Firmicutes	*Bacilli*	*Lactobacillales*	*Enterococcaceae*	*Enterococcus*	0.24	0.71
Firmicutes	*Bacilli*	*Erysipelotrichales*	*Erysipelatoclostridiaceae*	*Erysipelatoclostridium*	1.15	0.70
Firmicutes	*Bacilli*	*Erysipelotrichales*	*Erysipelotrichaceae*	*Turicibacter*	1.21	6.04
Firmicutes	*Bacilli*	*Erysipelotrichales*	*Erysipelotrichaceae*	*uncultured*	0.09	0.04
Firmicutes	*Bacilli*	*Erysipelotrichales*	*Erysipelotrichaceae*	*Merdibacter*	0.04	0.04
Firmicutes	*Bacilli*	*Erysipelotrichales*	*Erysipelotrichaceae*	*Erysipelotrichaceae*	0.02	0.06
Firmicutes	*Bacilli*	*Erysipelotrichales*	*Erysipelotrichaceae*	*[Clostridium]_innocuum_group*	0.02	0.00
Firmicutes	*Clostridia*	*Oscillospirales*	*Ethanoligenenaceae*	*Acetanaerobacterium*	0.00	0.00
Firmicutes	*Clostridia*	*Lachnospirales*	*Lachnospiraceae*	__	17.10	14.64
Firmicutes	*Clostridia*	*Lachnospirales*	*Lachnospiraceae*	*[Eubacterium]_hallii_group*	2.86	2.42
Firmicutes	*Clostridia*	*Lachnospirales*	*Lachnospiraceae*	*[Ruminococcus]_torques_group*	4.31	3.11
Firmicutes	*Clostridia*	*Lachnospirales*	*Lachnospiraceae*	*Sellimonas*	0.99	0.71
Firmicutes	*Clostridia*	*Lachnospirales*	*Lachnospiraceae*	*Blautia*	2.56	2.71
Firmicutes	*Clostridia*	*Lachnospirales*	*Lachnospiraceae*	*Anaerostipes*	1.20	1.20
Firmicutes	*Clostridia*	*Lachnospirales*	*Lachnospiraceae*	*[Eubacterium]_ventriosum_group*	0.24	0.63
Firmicutes	*Clostridia*	*Lachnospirales*	*Lachnospiraceae*	*GCA-900066575*	0.49	0.32
Firmicutes	*Clostridia*	*Lachnospirales*	*Lachnospiraceae*	*Lachnoclostridium*	0.50	0.44
Firmicutes	*Clostridia*	*Lachnospirales*	*Lachnospiraceae*	*[Ruminococcus]_gauvreauii_group*	0.29	0.20
Firmicutes	*Clostridia*	*Lachnospirales*	*Lachnospiraceae*	*Shuttleworthia*	0.24	0.10

Firmicutes	*Clostridia*	*Lachnospirales*	*Lachnospiraceae*	*Tuzzerella*	0.05	0.36
Firmicutes	*Clostridia*	*Lachnospirales*	*Lachnospiraceae*	*Tyzzerella*	0.13	0.12
Firmicutes	*Clostridia*	*Lachnospirales*	*Lachnospiraceae*	*Frisingicoccus*	0.01	0.34
Firmicutes	*Clostridia*	*Lachnospirales*	*Lachnospiraceae*	*ASF356*	0.13	0.12
Firmicutes	*Clostridia*	*Lachnospirales*	*Lachnospiraceae*	*Marvinbryantia*	0.05	0.26
Firmicutes	*Clostridia*	*Lachnospirales*	*Lachnospiraceae*	*CHKCI001*	0.10	0.14
Firmicutes	*Clostridia*	*Lachnospirales*	*Lachnospiraceae*	*UC5-1-2E3*	0.06	0.07
Firmicutes	*Clostridia*	*Lachnospirales*	*Lachnospiraceae*	*Lachnospiraceae_UCG-010*	0.03	0.03
Firmicutes	*Clostridia*	*Lachnospirales*	*Lachnospiraceae*	*Lachnospiraceae_FCS020_group*	0.01	0.02
Firmicutes	*Clostridia*	*Lachnospirales*	*Lachnospiraceae*	*Roseburia*	0.00	0.01
Firmicutes	*Clostridia*	*Lachnospirales*	*Lachnospiraceae*	*Catenibacillus*	0.00	0.00
Firmicutes	*Bacilli*	*Lactobacillales*	*Lactobacillaceae*	*Lactobacillus*	10.36	9.24
Firmicutes	*Bacilli*	*Lactobacillales*	*Lactobacillaceae*	*Pediococcus*	0.00	0.03
Firmicutes	*Bacilli*	*Lactobacillales*	*Leuconostocaceae*	*Weissella*	0.00	0.04
Actinobacteriota	*Actinobacteria*	*Micrococcales*	*Micrococcaceae*	*Enteractinococcus*	0.00	0.00
Firmicutes	*Clostridia*	*Monoglobales*	*Monoglobaceae*	*Monoglobus*	0.58	0.27
Firmicutes	*Clostridia*	*Oscillospirales*	*Oscillospiraceae*	*UCG-005*	1.25	2.97
Firmicutes	*Clostridia*	*Oscillospirales*	*Oscillospiraceae*	__	2.17	3.40
Firmicutes	*Clostridia*	*Oscillospirales*	*Oscillospiraceae*	*Oscillibacter*	0.32	0.24
Firmicutes	*Clostridia*	*Oscillospirales*	*Oscillospiraceae*	*Flavonifractor*	0.26	0.08
Firmicutes	*Clostridia*	*Oscillospirales*	*Oscillospiraceae*	*uncultured*	0.09	0.11
Firmicutes	*Clostridia*	*Oscillospirales*	*Oscillospiraceae*	*NK4A214_group*	0.10	0.06
Firmicutes	*Clostridia*	*Oscillospirales*	*Oscillospiraceae*	*Intestinimonas*	0.04	0.08
Firmicutes	*Clostridia*	*Oscillospirales*	*Oscillospiraceae*	*Colidextribacter*	0.07	0.05
Firmicutes	*Clostridia*	*Oscillospirales*	*Oscillospiraceae*	*Oscillospira*	0.00	0.02
Firmicutes	*Clostridia*	*Oscillospirales*	*Oscillospiraceae*	*Papillibacter*	0.00	0.00
Firmicutes	*Clostridia*	*Oscillospirales*	*Oscillospiraceae*	*Pseudoflavonifractor*	0.00	0.00
Firmicutes	*Clostridia*	*Oscillospirales*	*Oscillospirales*	*Hydrogenoanaerobacterium*	0.33	0.52
Firmicutes	*Clostridia*	*Peptococcales*	*Peptococcaceae*	*uncultured*	0.08	0.23
Firmicutes	*Clostridia*	*Peptostreptococcales-Tissierellales*	*Peptostreptococcaceae*	*Romboutsia*	1.79	2.76

Firmicutes	*Clostridia*	*Peptostreptococcales-Tissierellales*	*Peptostreptococcaceae*	__	0.34	0.03
Verrucomicrobiota	*Verrucomicrobiae*	*Opitutales*	*Puniceicoccaceae*	*uncultured*	0.00	0.03
Firmicutes	*Bacilli*	*RF39*	*RF39*	*RF39*	0.00	0.00
Bacteroidota	*Bacteroidia*	*Bacteroidales*	*Rikenellaceae*	*Alistipes*	0.00	0.06
Firmicutes	*Clostridia*	*Oscillospirales*	*Ruminococcaceae*	*Faecalibacterium*	11.48	5.60
Firmicutes	*Clostridia*	*Oscillospirales*	*Ruminococcaceae*	*Incertae_Sedis*	2.43	2.55
Firmicutes	*Clostridia*	*Oscillospirales*	*Ruminococcaceae*	*Subdoligranulum*	1.02	0.87
Firmicutes	*Clostridia*	*Oscillospirales*	*Ruminococcaceae*	*Anaerotruncus*	0.42	0.50
Firmicutes	*Clostridia*	*Oscillospirales*	*Ruminococcaceae*	*uncultured*	1.11	1.32
Firmicutes	*Clostridia*	*Oscillospirales*	*Ruminococcaceae*	*Negativibacillus*	0.47	0.41
Firmicutes	*Clostridia*	*Oscillospirales*	*Ruminococcaceae*	__	0.93	0.90
Firmicutes	*Clostridia*	*Oscillospirales*	*Ruminococcaceae*	*CAG-352*	0.17	0.27
Firmicutes	*Clostridia*	*Oscillospirales*	*Ruminococcaceae*	*Ruminococcus*	0.04	0.32
Firmicutes	*Clostridia*	*Oscillospirales*	*Ruminococcaceae*	*DTU089*	0.22	0.23
Firmicutes	*Clostridia*	*Oscillospirales*	*Ruminococcaceae*	*Caproiciproducens*	0.10	0.06
Firmicutes	*Clostridia*	*Oscillospirales*	*Ruminococcaceae*	*Paludicola*	0.03	0.08
Firmicutes	*Clostridia*	*Oscillospirales*	*Ruminococcaceae*	*[Eubacterium]_siraeum_group*	0.00	0.04
Firmicutes	*Clostridia*	*Oscillospirales*	*Ruminococcaceae*	*Harryflintia*	0.01	0.02
Firmicutes	*Clostridia*	*Oscillospirales*	*Ruminococcaceae*	*Fournierella*	0.00	0.02
Firmicutes	*Clostridia*	*Oscillospirales*	*Ruminococcaceae*	*Candidatus_Soleaferrea*	0.00	0.01
Firmicutes	*Clostridia*	*Oscillospirales*	*Ruminococcaceae*	*UBA1819*	0.00	0.00
Firmicutes	*Bacilli*	*Staphylococcales*	*Staphylococcaceae*	*Staphylococcus*	0.09	0.17
Firmicutes	*Bacilli*	*Staphylococcales*	*Staphylococcaceae*	*Jeotgalicoccus*	0.03	0.01
Firmicutes	*Bacilli*	*Lactobacillales*	*Streptococcaceae*	*Streptococcus*	0.99	1.96
Firmicutes	*Clostridia*	*Oscillospirales*	*UCG-010*	*UCG-010*	0.67	0.67
Proteobacteria	*Alphaproteobacteria*	*Rhodospirillales*	*uncultured*	*uncultured*	0.01	2.43
Firmicutes	*Clostridia*	*Oscillospirales*	*uncultured*	*uncultured*	0.01	0.01
Firmicutes	*Clostridia*	*uncultured*	*uncultured*	*uncultured*	0.00	0.01

International Journal of *Molecular Sciences*

MDPI

Supplementary Table S2. Examining the effects of *Salmonella* phage on the caecal microbiota in *Salmonella*-infected broilers. Bayesian statistical analysis of the relevant genera identified by partial least square-discriminant analysis (PLS-DA) in phage-treated chickens compared with the control group, computed as phage-treated *vs* control. Phage-treated group received 0.1 % *Salmonella* phage (10^8 PFU/g) *via* feed. The control group did not receive a phage.

Phylum	Family	Genus	HPD95 phage-control	P0 phage-control	D phage-control
Firmicutes	*Streptococcaceae*	*Streptococcus*	[-0.03,1.59]	96.71	0.76
	Staphylococcaceae	*Faecalibacterium*	[-1.51,0.15]	94.89	-0.68
		Jeotgalicoccus	[-0.71,1.05]	64.42	0.16
	Ruminococcaceae	*Paludicola*	[-0.2,1.47]	92.69	0.61
	Peptostreptococcaceae	*Romboutsia*	[0.11,1.68]	98.62	0.90
	Oscillospirales	*Hydrogenoanaerobacterium*	[0.04,1.65]	97.99	0.84
	Oscillospiraceae	*UCG005*	[0.66,1.97]	99.98	1.31
		Flavonifractor	[-1.37,0.33]	88.70	-0.51
	Monoglobaceae	*Monoglobus*	[-1.49,0.16]	94.64	-0.67
	Leuconostocaceae	*Weissella*	[-0.18,1.5]	92.95	0.62
	Lachnospiraceae	*Frisingicoccus*	[0.63,1.95]	99.97	1.29
		Marvinbryantia	[0.55,1.94]	99.89	1.22
	Erysipelotrichaceae	*Turicibacter*	[0.77,2.01]	99.99	1.40
	Erysipelatoclostridiaceae	*Erysipelatoclostridium*	[-1.65,-0.09]	98.64	-0.89
	Clostridia_vadinBB60_group	*Clostridia_vadinBB60_group*	[-1.25,0.49]	81.10	-0.37
	Anaerovoracaceae	*Family_XIII_UCG001*	[-0.25,1.43]	92.26	0.60
Bacteroidota	*Bacteroidaceae*	*Bacteroides*	[0.7,1.98]	99.99	1.33

HPD95phage-control = The highest posterior density region at 95% of probability. P0= Probability of the difference (Dphage-control) being greater than 0 when Dphage-control > 0 or lower than 0 when Dphage-control < 0. Dphage-control = Mean of the difference - phage-treated-control (median of the marginal posterior distribution of the difference between the control group phage-treated group). Statistical differences were assumed if ∣ Dphage-control ∣ surpass R value and its P0>0.90.

Supplementary Table S3. Examining the effects of *Salmonella* phage on the caecal metabolome in *Salmonella*-infected broilers. Relative abundance (%) of identified caecal metabolites.

Met	m/z	RT [min]	Reference Ion	control	phage-treated
MET 1	304.90793	0.878	[M-H]-1	0.0075	0.0073
MET 2	296.96456	0.897	[M+H]+1	0.0164	0.0166
MET 3	688.86828	0.98	[M-2H]-2	0.0047	0.0050
MET 4	500.9197	0.982	[M-H]-1	0.0475	0.0478
MET 5	586.90512	0.983	[M-H]-1	0.0080	0.0076
MET 6	472.92451	0.984	[M-H]-1	0.0208	0.0212
MET 7	602.88287	0.985	[M-H]-1	0.0199	0.0222
MET 8	744.858	0.986	[M-2H]-2	0.0078	0.0093
MET 9	488.9023	0.988	[M-H]-1	0.0120	0.0137
MET 10	646.8551	0.989	[M-2H]-2	0.0145	0.0164
MET 11	760.83562	0.989	[M-2H]-2	0.0102	0.0126
MET 12	516.89697	0.993	[M-H]-1	0.1228	0.1371
MET 13	678.81029	1	[M-H]-1	0.0076	0.0084
MET 14	548.8523	1	[M-H]-1	0.0468	0.0521
MET 15	402.91668	1.003	[M-H]-1	0.3673	0.4167
MET 16	166.0644	1.005	[M+H]+1	0.0999	0.1109
MET 17	418.89439	1.006	[M-H]-1	0.1690	0.1937
MET 18	164.06675	1.006	[M+H-H2O]+1	0.6822	0.7448
MET 19	171.99157	1.007	[M+H-H2O]+1	0.0502	0.0557
MET 20	434.87181	1.007	[M-H]-1	0.0310	0.0361
MET 21	164.06681	1.009	[M+H]+1	0.6822	0.7448
MET 22	171.00552	1.01	[M+H-H2O]+1	0.0820	0.0897
MET 23	129.02263	1.01	[M+H]+1	0.0205	0.0221
MET 24	123.04052	1.01	[M+H-H2O]+1	43.081	46.886
MET 25	125.03801	1.01	[M+H-H2O]+1	0.6329	0.6932
MET 26	112.00649	1.011	[M+H]+1	0.1418	0.1547
MET 27	152.036	1.012	[M+H-H2O]+1	0.8155	0.8891
MET 28	153.03264	1.012	[M+H-H2O]+1	10.476	11.307
MET 29	139.01787	1.012	[M+H-H2O]+1	0.8138	0.9070
MET 30	151.0352	1.013	[M+H-H2O]+1	71.704	77.642
MET 31	128.01934	1.013	[M+2H]+2	0.6601	0.7209
MET 32	167.0127	1.013	[M+H-H2O]+1	35.791	39.897
MET 33	265.01536	1.015	[M+H-H2O]+1	16.727	17.884
MET 34	125.98631	1.015	[M+H-H2O]+1	18.219	20.461
MET 35	110.00891	1.015	[M+H-H2O]+1	10.160	11.125
MET 36	129.97918	1.015	[M+H-H2O]+1	0.0390	0.0441
MET 37	208.03924	1.015	[M+H]+1	0.1383	0.1557
MET 38	112.00645	1.015	[M+H-H2O]+1	0.1418	0.1547
MET 39	241.99939	1.016	[M+H]+1	0.6044	0.6348
MET 40	243.95932	1.017	[M+H-H2O]+1	0.0204	0.0228
MET 41	439.93221	1.018	[M+2H]+2	0.0530	0.0580
MET 42	147.516	1.019	[M+2H]+2	0.1045	0.1211
MET 43	290.93381	1.019	[M-H]-1	0.0820	0.0941
MET 44	304.91398	1.021	[M-H]-1	0.1207	0.1434
MET 45	288.93639	1.022	[M-H]-1	0.5903	0.6819
MET 46	241.96402	1.023	[M+H-H2O]+1	0.1342	0.1486
MET 47	239.9664	1.023	[M+H-H2O]+1	0.9603	10.689
MET 48	282.99037	1.024	[M+H]+1	0.1185	0.1340
MET 49	198.9393	1.024	[M+H-H2O]+1	0.0585	0.0689
MET 50	129.13869	1.027	[M+H]+1	0.1944	0.2040
MET 51	353.94663	1.028	[M+H-H2O]+1	0.0895	0.0947
MET 52	214.91706	1.028	[M+H-H2O]+1	0.0307	0.0357
MET 53	325.95157	1.029	[M+H]+1	0.0275	0.0296
MET 54	255.94396	1.029	[M+H-H2O]+1	0.2500	0.2802

MET 55	275.95698	1.032	[M-H]-1	0.0452	0.0497
MET 56	385.90189	1.032	[M+H-H2O]+1	0.0279	0.0312
MET 57	272.95894	1.034	[M-H]-1	12.382	13.705
MET 58	387.93493	1.035	[M+H]+1	0.0174	0.0197
MET 59	296.97067	1.035	[M+H]+1	0.0928	0.1048
MET 60	442.87801	1.035	[M+H]+1	0.0253	0.0294
MET 61	483.90477	1.038	[M+H]+1	0.0225	0.0255
MET 62	147.1128	1.068	[M+H]+1	0.2723	0.2834
MET 63	616.83697	1.069	[M-H]-1	0.0133	0.0150
MET 64	188.17568	1.073	[M+H]+1	0.3839	0.3194
MET 65	486.87878	1.074	[M-H]-1	0.0363	0.0417
MET 66	684.82183	1.084	[M-H]-1	0.0067	0.0081
MET 67	372.89786	1.089	[M-H]-1	0.0415	0.0437
MET 68	554.8637	1.09	[M-H]-1	0.0156	0.0160
MET 69	570.84075	1.09	[M-H]-1	0.0209	0.0223
MET 70	175.11888	1.096	[M+H]+1	0.2869	0.2790
MET 71	586.81784	1.096	[M-H]-1	0.0116	0.0111
MET 72	424.90814	1.098	[M-H]-1	0.0166	0.0208
MET 73	540.82152	1.1	[M-H]-1	0.0113	0.0092
MET 74	440.88215	1.102	[M-H]-1	0.0479	0.0411
MET 75	524.84457	1.104	[M-H]-1	0.0151	0.0110
MET 76	294.95326	1.104	[M-H]-1	0.0050	0.0086
MET 77	226.96546	1.107	[M-H]-1	0.0504	0.0674
MET 78	307.94989	1.107	[M+H]+1	0.0094	0.0082
MET 79	282.90091	1.107	[M+H-H2O]+1	0.0100	0.0091
MET 80	342.87875	1.108	[M-H]-1	0.0161	0.0124
MET 81	242.9393	1.109	[M-H]-1	0.0744	0.0632
MET 82	310.92733	1.112	[M-H]-1	0.0216	0.0218
MET 83	326.90126	1.112	[M-H]-1	0.0426	0.0276
MET 84	410.86316	1.114	[M-H]-1	0.0252	0.0133
MET 85	394.88885	1.114	[M-H]-1	0.0194	0.0158
MET 86	236.90459	1.116	[M+H]+1	0.0263	0.0172
MET 87	535.23811	1.139	[M-H]-1	0.0443	0.0330
MET 88	148.13323	1.14	[M+H]+1	0.1241	0.0753
MET 89	381.94398	1.154	[M-H]-1	0.0057	0.0044
MET 90	411.96285	1.154	[M-H]-1	0.0042	0.0042
MET 91	491.247	1.157	[M+H]+1	0.0200	0.0209
MET 92	297.98186	1.164	[M-H]-1	0.0133	0.0114
MET 93	161.09206	1.164	[M+H]+1	0.0604	0.0494
MET 94	343.97864	1.165	[M-H]-1	0.0070	0.0087
MET 95	328.0012	1.168	[M-H]-1	0.0075	0.0086
MET 96	405.91079	1.169	[M-H]-1	0.0045	0.0045
MET 97	176.10298	1.17	[M+NH4]+1	0.0400	0.0442
MET 98	230.00685	1.173	[M-H]-1	0.0176	0.0136
MET 99	229.99789	1.176	[M-H-H2O]-1	0.0196	0.0209
MET 100	321.94904	1.176	[M-H]-1	0.0085	0.0110
MET 101	367.94558	1.176	[M-H]-1	0.0043	0.0090
MET 102	318.97567	1.177	[M+H]+1	0.0104	0.0163
MET 103	214.02016	1.177	[M-H-H2O]-1	0.0367	0.0377
MET 104	351.96821	1.178	[M-H]-1	0.0038	0.0082
MET 105	302.99819	1.178	[M+H-H2O]+1	0.0079	0.0130
MET 106	134.04589	1.181	[M-H-H2O]-1	0.0230	0.0127
MET 107	231.0109	1.181	[M+H]+1	0.0062	0.0065
MET 108	227.03389	1.182	[M+H-H2O]+1	0.0214	0.0220
MET 109	211.05636	1.182	[M+H-H2O]+1	0.0088	0.0086
MET 110	353.02462	1.183	[M-H]-1	0.0047	0.0035
MET 111	216.03541	1.183	[M+H]+1	0.0300	0.0279
MET 112	202.01975	1.185	[M+H]+1	0.0034	0.0037
MET 113	172.51602	1.185	[M+2H]+2	0.0048	0.0071

MET 114	237.98741	1.187	[M-H]-1	0.0237	0.0386
MET 115	225.06116	1.19	[M-H]-1	0.0175	0.0235
MET 116	246.02195	1.19	[M+H]+1	0.0288	0.0403
MET 117	204.99543	1.191	[M+H-H2O]+1	0.0241	0.0341
MET 118	315.06864	1.191	[M-H]-1	0.0099	0.0082
MET 119	207.02849	1.193	[M+H]+1	0.0133	0.0186
MET 120	230.04444	1.195	[M+H]+1	0.0436	0.0616
MET 121	387.11475	1.195	[M-H]-1	0.0113	0.0132
MET 122	132.52005	1.195	[M+2H]+2	0.0265	0.0363
MET 123	226.03864	1.196	[M-H]-1	0.0109	0.0118
MET 124	124.53137	1.197	[M+2H]+2	0.0543	0.0778
MET 125	163.9778	1.198	[M+H]+1	0.0614	0.0568
MET 126	293.03009	1.199	[M-H]-1	0.0132	0.0166
MET 127	286.97802	1.2	[M-H]-1	0.0312	0.0369
MET 128	309.01655	1.201	[M-H]-1	0.0219	0.0176
MET 129	549.16759	1.202	[M-H]-1	0.0067	0.0067
MET 130	124.00606	1.202	[M-H]-1	0.1004	0.1356
MET 131	146.04461	1.202	[M-H]-1	0.2312	0.2061
MET 132	238.09291	1.204	[M-H]-1	0.0073	0.0037
MET 133	148.06046	1.212	[M+H]+1	24.554	22.336
MET 134	160.13315	1.213	[M+H]+1	0.9907	0.7870
MET 135	175.10784	1.215	[M+H]+1	0.0410	0.0356
MET 136	249.02189	1.218	[M-H]-1	0.0324	0.0450
MET 137	116.07092	1.228	[M+H]+1	0.1307	0.1364
MET 138	188.02265	1.255	[M+H-H2O]+1	0.1664	0.1932
MET 139	148.94854	1.257	[M-H]-1	0.0044	0.0047
MET 140	256.05934	1.258	[M-H]-1	0.0115	0.0132
MET 141	215.03227	1.259	[M-H]-1	0.0215	0.0238
MET 142	468.15144	1.26	[M-H]-1	0.0138	0.0108
MET 143	160.02767	1.262	[M+H-H2O]+1	0.0592	0.0722
MET 144	130.08636	1.266	[M+H]+1	0.2953	0.3346
MET 145	162.97342	1.266	[M+H-H2O]+1	0.0779	0.1000
MET 146	227.95854	1.27	[M-H]-1	0.0057	0.0083
MET 147	118.08652	1.273	[M+H]+1	0.1983	0.1760
MET 148	131.03254	1.274	[M+2H]+2	0.0486	0.0596
MET 149	377.08586	1.275	[M-H]-1	0.0109	0.0115
MET 150	119.5247	1.276	[M+2H]+2	0.1657	0.2034
MET 151	239.04377	1.278	[M-H-H2O]-1	0.0083	0.0058
MET 152	139.02113	1.278	[M+2H]+2	0.0518	0.0637
MET 153	181.96507	1.279	[M-H]-1	0.0067	0.0067
MET 154	204.12302	1.28	[M+H]+1	0.0327	0.0366
MET 155	127.51341	1.28	[M+2H]+2	0.1627	0.2010
MET 156	197.93912	1.28	[M-H]-1	0.0145	0.0108
MET 157	313.08097	1.286	[M-H]-1	0.0196	0.0205
MET 158	539.1389	1.288	[M-H]-1	0.0080	0.0075
MET 159	217.92961	1.288	[M-H]-1	0.0056	0.0081
MET 160	164.83497	1.29	[M-H]-1	0.0089	0.0108
MET 161	138.91967	1.291	[M-H]-1	0.0031	0.0041
MET 162	160.84078	1.293	[M-H]-1	0.0289	0.0348
MET 163	162.076	1.294	[M+NH4]+1	0.1977	0.2045
MET 164	227.00688	1.298	[M+H]+1	0.1293	0.1471
MET 165	124.99007	1.306	[M-H]-1	0.0104	0.0133
MET 166	240.98736	1.307	[M+H]+1	0.4946	0.5995
MET 167	256.00752	1.308	[M+H]+1	0.0291	0.0446
MET 168	197.80719	1.308	[M-H]-1	0.4462	0.5852
MET 169	121.06495	1.309	[M+H]+1	0.0117	0.0143
MET 170	257.11422	1.311	[M-H-H2O]-1	0.0826	0.0755
MET 171	326.05738	1.314	[M+H]+1	0.0193	0.0169
MET 172	319.99635	1.315	[M+H]+1	0.0432	0.0594

MET 173	304.01844	1.315	[M+H]+1	0.0851	0.1244
MET 174	182.08108	1.319	[M+H]+1	0.1040	0.1024
MET 175	292.99487	1.32	[M+H]+1	0.4759	0.6577
MET 176	319.00116	1.32	[M+H]+1	0.1870	0.2287
MET 177	303.02362	1.328	[M+H]+1	0.2235	0.2434
MET 178	116.97732	1.331	[M+H]+1	0.0546	0.0431
MET 179	149.52608	1.333	[M+2H]+2	0.1702	0.1974
MET 180	308.97232	1.336	[M+H]+1	0.0856	0.1173
MET 181	176.00849	1.346	[M+2H]+2	0.5272	0.5873
MET 182	146.0069	1.35	[M+2H]+2	0.5549	0.6322
MET 183	168.01973	1.351	[M+2H]+2	0.9090	10.066
MET 184	147.00474	1.351	[M+2H]+2	0.0794	0.0905
MET 185	221.97435	1.354	[M-H]-1	0.0153	0.0169
MET 186	129.01287	1.361	[M+2H]+2	0.2707	0.3075
MET 187	138.01813	1.362	[M+2H]+2	0.7546	0.8424
MET 188	167.99612	1.362	[M-H]-1	0.0050	0.0045
MET 189	174.03979	1.37	[M-H]-1	0.0416	0.0344
MET 190	158.0345	1.375	[M+H]+1	0.1873	0.1958
MET 191	157.03509	1.382	[M+H]+1	117.360	122.817
MET 192	256.95962	1.383	[M+H]+1	0.0989	0.0892
MET 193	253.97447	1.455	[M-H]-1	0.0021	0.0041
MET 194	300.04889	1.461	[M-H]-1	0.0317	0.0250
MET 195	276.05674	1.461	[M+H]+1	0.0174	0.0323
MET 196	210.04349	1.465	[M-H]-1	0.0223	0.0549
MET 197	293.99616	1.472	[M-H]-1	0.0159	0.0303
MET 198	279.03893	1.671	[M-H]-1	0.0089	0.0073
MET 199	124.03944	1.671	[M+H]+1	0.0649	0.1223
MET 200	355.08184	1.671	[M-H]-1	0.0200	0.0159
MET 201	244.20175	1.673	[M+H]+1	0.0113	0.0235
MET 202	468.15112	1.676	[M-H]-1	0.0049	0.0047
MET 203	174.03978	1.676	[M-H]-1	0.0153	0.0138
MET 204	257.11425	1.678	[M-H]-1	0.0588	0.0572
MET 205	268.1037	1.681	[M+H]+1	0.1476	0.1706
MET 206	122.09659	1.718	[M+H]+1	0.0213	0.0252
MET 207	190.07088	1.728	[M+H]+1	0.1491	0.1226
MET 208	303.05023	1.734	[M-H]-1	0.0069	0.0064
MET 209	267.0735	1.743	[M-H]-1	0.0277	0.0279
MET 210	273.1806	1.807	[M+H]+1	0.0166	0.0056
MET 211	277.86083	1.816	[M-H]-1	0.0095	0.0063
MET 212	335.86656	1.82	[M-H]-1	0.0024	0.0027
MET 213	261.00736	1.864	[M-H]-1	0.4786	0.5513
MET 214	275.19638	1.952	[M+H]+1	0.0239	0.0052
MET 215	238.09341	1.997	[M+H]+1	0.0086	0.0094
MET 216	355.18623	2.011	[M+H]+1	0.0162	0.0038
MET 217	169.01653	2.072	[M-H]-1	0.0622	0.1737
MET 218	233.14951	2.136	[M+H]+1	0.0287	0.0207
MET 219	245.01229	2.163	[M-H]-1	0.1548	0.1323
MET 220	238.03877	2.213	[M-H]-1	0.0489	0.1418
MET 221	179.03738	2.332	[M-H]-1	0.0061	0.0082
MET 222	231.96637	2.347	[M+H]+1	0.0106	0.0101
MET 223	272.99283	2.349	[M+H]+1	0.0219	0.0211
MET 224	227.99524	2.351	[M+H]+1	0.0745	0.0723
MET 225	285.05922	2.352	[M-H]-1	0.0082	0.0080
MET 226	229.99552	2.354	[M+H]+1	0.0058	0.0059
MET 227	166.08623	2.359	[M+NH4]+1	0.3727	0.3283
MET 228	120.08098	2.359	[M+H]+1	0.0547	0.0473
MET 229	131.03364	2.367	[M-H]-1	0.0197	0.0206
MET 230	254.07028	2.432	[M-H]-1	0.0223	0.0250
MET 231	371.06607	2.443	[M+H]+1	0.0056	0.0070

MET 232	130.06526	2.444	[M+H]+1	0.0098	0.0111
MET 233	364.22292	2.46	[M+H]+1	0.0087	0.0071
MET 234	277.05961	2.465	[M-H]-1	0.0024	0.0031
MET 235	253.12935	2.473	[M+H]+1	0.0061	0.0065
MET 236	383.10235	2.483	[M+H]+1	0.0081	0.0111
MET 237	424.12893	2.485	[M+H]+1	0.0024	0.0032
MET 238	326.05576	2.49	[M+H]+1	0.0110	0.0143
MET 239	383.10236	2.49	[M+H-H2O]+1	0.0081	0.0111
MET 240	298.06091	2.49	[M+H]+1	0.0132	0.0173
MET 241	257.03433	2.491	[M+H-H2O]+1	0.0135	0.0172
MET 242	370.0725	2.492	[M-H]-1	0.0064	0.0073
MET 243	281.05816	2.494	[M+H]+1	0.0057	0.0073
MET 244	285.02923	2.495	[M+H]+1	0.0247	0.0315
MET 245	333.99939	2.496	[M-H]-1	0.0033	0.0048
MET 246	224.0553	2.501	[M+H]+1	0.0424	0.0362
MET 247	162.05491	2.503	[M+H]+1	0.0464	0.0402
MET 248	295.16507	2.508	[M+H]+1	0.0195	0.0115
MET 249	271.99946	2.523	[M-H]-1	0.0126	0.0158
MET 250	509.27587	2.527	[M+H]+1	0.0127	0.0083
MET 251	232.15424	2.579	[M+H]+1	0.0182	0.0199
MET 252	247.07468	2.726	[M+H]+1	0.0028	0.0024
MET 253	186.99252	2.772	[M+H-H2O]+1	0.0158	0.0241
MET 254	214.98745	2.773	[M+H]+1	0.0178	0.0271
MET 255	146.11754	2.957	[M+H]+1	0.0163	0.0153
MET 256	194.04842	3.005	[M-H]-1	0.0712	0.0760
MET 257	259.2014	3.022	[M+H]+1	0.0034	0.0045
MET 258	220.11787	3.038	[M+H]+1	0.0230	0.0313
MET 259	269.02171	3.058	[M+H]+1	0.0263	0.0265
MET 260	285.05918	3.06	[M-H]-1	0.0066	0.0062
MET 261	131.03363	3.065	[M-H]-1	0.0208	0.0202
MET 262	158.08113	3.068	[M+H]+1	0.1085	0.1364
MET 263	156.06539	3.074	[M-H]-1	0.0178	0.0278
MET 264	258.09821	3.163	[M-H]-1	0.0207	0.0268
MET 265	493.28083	3.197	[M+H]+1	0.0114	0.0099
MET 266	331.19016	3.282	[M+H]+1	0.0080	0.0007
MET 267	383.20765	3.282	[M-H]-1	0.1175	0.0055
MET 268	237.12322	3.289	[M+H]+1	0.0149	0.0163
MET 269	248.11399	3.594	[M+H]+1	0.0322	0.0282
MET 270	208.06035	3.607	[M+H]+1	0.0344	0.0335
MET 271	314.20708	3.64	[M+H]+1	0.0271	0.0256
MET 272	138.05496	3.733	[M+H]+1	0.0419	0.0607
MET 273	231.17017	3.77	[M+H]+1	0.0098	0.0072
MET 274	214.01727	3.809	[M-H]-1	0.0528	0.0764
MET 275	153.01806	3.861	[M-H]-1	0.0260	0.0257
MET 276	154.04982	3.864	[M+H]+1	0.0141	0.0170
MET 277	167.04504	3.869	[M+H]+1	0.0080	0.0084
MET 278	187.14393	3.87	[M+H]+1	0.0017	0.0621
MET 279	192.0688	3.893	[M+H]+1	0.0068	0.0093
MET 280	188.07047	3.93	[M+H]+1	0.0598	0.0469
MET 281	144.08073	4.043	[M+H]+1	0.0086	0.0070
MET 282	298.09655	4.051	[M+H]+1	0.1239	0.0963
MET 283	342.0877	4.055	[M-H]-1	0.0081	0.0059
MET 284	242.0108	4.101	[M+H]+1	0.0108	0.0107
MET 285	331.22238	4.108	[M+H]+1	0.0108	0.0093
MET 286	466.16097	4.15	[M+H]+1	0.0381	0.0317
MET 287	379.13996	4.287	[M-H]-1	0.0148	0.0150
MET 288	319.22252	4.352	[M+H]+1	0.0026	0.0025
MET 289	187.10762	4.607	[M+H]+1	0.0079	0.0077
MET 290	328.72038	4.761	[M+2H]+2	0.0055	0.0084

MET 291	153.02147	4.993	[M-H]-1	0.0230	0.0484
MET 292	114.09156	5.102	[M+H]+1	0.3113	0.4674
MET 293	192.06882	5.154	[M+H]+1	0.0038	0.0043
MET 294	326.03732	5.172	[M+H]+1	0.0091	0.0070
MET 295	210.11238	5.228	[M+H]+1	0.0069	0.0063
MET 296	241.11809	5.238	[M+H]+1	0.0341	0.0335
MET 297	197.1284	5.239	[M+H]+1	0.0316	0.0298
MET 298	191.11779	5.335	[M+H]+1	0.0049	0.0035
MET 299	356.95983	5.344	[M+H-H2O]+1	0.0102	0.0076
MET 300	357.9676	5.345	[M+H]+1	0.0331	0.0238
MET 301	261.00725	5.355	[M-H]-1	34.138	24.620
MET 302	360.93229	5.359	[M-H]-1	0.0362	0.0293
MET 303	397.98639	5.361	[M+H]+1	0.0352	0.0249
MET 304	137.02672	5.379	[M+H]+1	0.0018	0.0020
MET 305	306.0969	5.448	[M+H]+1	0.0062	0.0042
MET 306	553.30183	5.499	[M+H]+1	0.0055	0.0047
MET 307	184.02796	5.537	[M+2H]+2	0.0094	0.0059
MET 308	499.32766	5.542	[M+H]+1	0.0059	0.0044
MET 309	341.17038	5.543	[M+H]+1	0.0117	0.0082
MET 310	190.04977	5.55	[M+H]+1	0.2505	0.1562
MET 311	331.00082	5.556	[M+H]+1	0.0084	0.0048
MET 312	256.02256	5.559	[M-H]-1	0.0114	0.0067
MET 313	247.10749	5.564	[M+H]+1	0.0184	0.0172
MET 314	210.11233	5.593	[M+H]+1	0.0113	0.0130
MET 315	295.13976	5.599	[M+NH4]+1	0.0100	0.0106
MET 316	322.10437	5.601	[M-H]-1	0.0129	0.0127
MET 317	292.15267	5.621	[M+2H]+2	0.0074	0.0074
MET 318	229.003	5.656	[M+H]+1	0.0465	0.0425
MET 319	381.18389	5.657	[M+H]+1	0.0085	0.0071
MET 320	270.02947	5.657	[M+H]+1	0.0147	0.0137
MET 321	201.0081	5.659	[M+H-H2O]+1	0.0355	0.0324
MET 322	271.04982	5.662	[M+H-H2O]+1	0.0263	0.0221
MET 323	242.03459	5.663	[M+H-H2O]+1	0.0233	0.0213
MET 324	312.07634	5.664	[M+H]+1	0.0042	0.0035
MET 325	270.02945	5.664	[M+H-H2O]+1	0.0147	0.0137
MET 326	225.03194	5.671	[M+H]+1	0.0125	0.0103
MET 327	321.02909	5.685	[M+H]+1	0.0056	0.0058
MET 328	275.01107	5.688	[M+H]+1	0.0090	0.0101
MET 329	233.98438	5.69	[M+H-H2O]+1	0.0013	0.0014
MET 330	335.72818	5.706	[M+2H]+2	0.0071	0.0105
MET 331	379.13985	5.708	[M-H]-1	0.0165	0.0159
MET 332	285.10784	5.721	[M+H]+1	0.0355	0.0326
MET 333	411.15477	5.747	[M+H]+1	0.0085	0.0076
MET 334	217.09704	5.79	[M+H]+1	0.0407	0.0427
MET 335	266.17485	5.798	[M+H]+1	0.0025	0.0022
MET 336	208.06418	5.825	[M-H]-1	0.0210	0.0305
MET 337	153.01806	5.857	[M-H]-1	0.0415	0.0343
MET 338	160.03918	5.87	[M-H]-1	0.0462	0.0400
MET 339	162.05481	5.904	[M-H]-1	0.2924	0.2564
MET 340	360.07258	5.961	[M-H]-1	0.0256	0.0216
MET 341	265.08155	5.994	[M+H]+1	0.0090	0.0057
MET 342	173.53897	6.016	[M+2H]+2	0.0205	0.0229
MET 343	435.08071	6.021	[M-H]-1	0.0560	0.0603
MET 344	307.98473	6.022	[M+H]+1	0.0362	0.0380
MET 345	675.0769	6.022	[M+H]+1	0.0159	0.0183
MET 346	413.09899	6.023	[M-H]-1	0.7209	0.8538
MET 347	162.05476	6.023	[M+H]+1	0.3698	0.4189
MET 348	235.01972	6.023	[M+2H]+2	0.0164	0.0178
MET 349	469.0322	6.023	[M+2H]+2	0.0582	0.0662

MET 350	466.01005	6.023	[M-H]-1	0.0221	0.0261
MET 351	348.00349	6.024	[M+H]+1	0.0236	0.0315
MET 352	206.04505	6.024	[M-H]-1	29.087	33.170
MET 353	880.11715	6.024	[M-H]-1	0.0075	0.0093
MET 354	171.52221	6.024	[M+2H]+2	0.0118	0.0126
MET 355	303.00581	6.026	[M+H]+1	0.0532	0.0570
MET 356	134.06001	6.026	[M+H]+1	0.0068	0.0078
MET 357	642.13465	6.026	[M-H]-1	0.0246	0.0298
MET 358	164.53371	6.029	[M+2H]+2	0.0185	0.0204
MET 359	305.97072	6.029	[M-H-H2O]-1	0.0873	0.0939
MET 360	242.02212	6.029	[M-H]-1	0.0540	0.0590
MET 361	171.52221	6.03	[M+H]+1	0.0122	0.0125
MET 362	552.15124	6.039	[M-H]-1	0.0091	0.0106
MET 363	274.03325	6.039	[M-H]-1	0.0712	0.0716
MET 364	269.04142	6.044	[M-H]-1	0.1450	0.1601
MET 365	321.06484	6.07	[M-H]-1	0.0488	0.0399
MET 366	273.18048	6.098	[M+H]+1	0.0097	0.0094
MET 367	231.11266	6.206	[M+H]+1	0.0177	0.0159
MET 368	231.11264	6.209	[M+NH4]+1	0.0177	0.0159
MET 369	377.12436	6.25	[M-H-H2O]-1	0.0033	0.0053
MET 370	204.02954	6.252	[M-H]-1	0.0249	0.0247
MET 371	219.11268	6.291	[M+H]+1	0.0050	0.0049
MET 372	359.07749	6.299	[M-H]-1	0.0176	0.0149
MET 373	473.15669	6.303	[M-H]-1	0.0066	0.0058
MET 374	442.26982	6.353	[M+H]+1	0.0043	0.0042
MET 375	213.07326	6.364	[M+Na]+1	0.0353	0.0123
MET 376	256.97609	6.369	[M-H]-1	0.0286	0.0217
MET 377	256.02649	6.396	[M+H]+1	0.0052	0.0067
MET 378	159.06511	6.396	[M-H]-1	0.0304	0.0375
MET 379	195.06896	6.421	[M-H]-1	0.0072	0.0056
MET 380	291.16307	6.421	[M+2H]+2	0.0306	0.0265
MET 381	276.15786	6.435	[M+2H]+2	0.0352	0.0314
MET 382	399.09965	6.487	[M-H]-1	0.0593	0.0365
MET 383	308.11744	6.487	[M-H]-1	0.0071	0.0110
MET 384	272.05992	6.492	[M-H]-1	0.0546	0.0049
MET 385	458.26473	6.499	[M+H]+1	0.0024	0.0042
MET 386	286.00278	6.505	[M-H]-1	0.0179	0.1007
MET 387	386.0884	6.53	[M-H]-1	0.0136	0.0180
MET 388	247.14393	6.538	[M+2H]+2	0.0431	0.0414
MET 389	269.14928	6.572	[M+H]+1	0.0079	0.0096
MET 390	224.05597	6.584	[M-H]-1	0.0187	0.0196
MET 391	306.16833	6.626	[M+2H]+2	0.0050	0.0045
MET 392	231.11267	6.657	[M+H]+1	0.0077	0.0067
MET 393	267.09089	6.688	[M-H]-1	0.0269	0.0213
MET 394	292.17082	6.693	[M+2H]+2	0.0169	0.0065
MET 395	258.20617	6.764	[M+H]+1	0.0343	0.0275
MET 396	262.11839	6.778	[M+H]+1	0.0115	0.0107
MET 397	244.09666	6.796	[M+H]+1	0.0095	0.0104
MET 398	526.11416	6.812	[M-H]-1	0.0004	0.0043
MET 399	381.19199	6.813	[M-H]-1	0.0018	0.1540
MET 400	449.17942	6.813	[M-H]-1	0.0004	0.0075
MET 401	523.14984	6.816	[M+H]+1	0.0003	0.0041
MET 402	481.11716	6.816	[M-H]-1	0.0004	0.0051
MET 403	137.05948	6.83	[M-H]-1	0.0032	0.0042
MET 404	289.02674	6.847	[M+H]+1	0.0015	0.0019
MET 405	307.17615	6.873	[M+2H]+2	0.0088	0.0046
MET 406	162.05489	6.892	[M-H]-1	0.0074	0.0045
MET 407	333.13299	6.92	[M+H]+1	0.0123	0.0088
MET 408	449.05462	6.923	[M-H]-1	0.0019	0.0013

MET 409	354.14455	6.963	[M+H]+1	0.0071	0.0069
MET 410	269.14929	7.109	[M+H]+1	0.0117	0.0162
MET 411	224.09155	7.112	[M+H]+1	0.1260	0.1672
MET 412	290.06465	7.113	[M-H]-1	0.0032	0.0047
MET 413	258.05371	7.115	[M-H]-1	0.0026	0.0028
MET 414	469.15791	7.115	[M+Na]+1	0.0012	0.0008
MET 415	285.07282	7.117	[M-H]-1	0.0085	0.0075
MET 416	268.0827	7.12	[M-H]-1	0.0124	0.0114
MET 417	442.26965	7.124	[M+H]+1	0.0145	0.0335
MET 418	157.04941	7.125	[M-H]-1	0.0236	0.0262
MET 419	372.14384	7.138	[M+H]+1	0.0026	0.0029
MET 420	574.312	7.151	[M+H]+1	0.0031	0.0037
MET 421	377.14515	7.157	[M+H]+1	0.0031	0.0028
MET 422	269.17443	7.159	[M+H]+1	0.0524	0.0284
MET 423	382.13083	7.162	[M+H]+1	0.0064	0.0032
MET 424	285.17072	7.162	[M-H]-1	0.1494	0.0784
MET 425	353.15821	7.164	[M-H]-1	0.0062	0.0033
MET 426	430.09312	7.167	[M-H]-1	0.0037	0.0021
MET 427	348.16636	7.167	[M-H]-1	0.0151	0.0081
MET 428	192.10175	7.27	[M+H]+1	0.0179	0.0247
MET 429	384.07272	7.292	[M-H]-1	0.0122	0.0092
MET 430	246.00753	7.307	[M-H]-1	0.0115	0.0159
MET 431	227.08136	7.318	[M+H]+1	0.0055	0.0061
MET 432	343.12944	7.337	[M+Na]+1	0.0105	0.0098
MET 433	144.04417	7.356	[M-H]-1	0.0185	0.0414
MET 434	383.1233	7.356	[M+H]+1	0.0012	0.0033
MET 435	258.03828	7.359	[M-H]-1	0.0059	0.0125
MET 436	333.01655	7.367	[M+H]+1	0.0061	0.0139
MET 437	211.02752	7.381	[M-H]-1	0.1729	0.1842
MET 438	160.03921	7.405	[M-H]-1	0.0200	0.0210
MET 439	162.05486	7.407	[M+H]+1	0.0553	0.0457
MET 440	440.25414	7.435	[M+H]+1	0.0103	0.0100
MET 441	253.15443	7.486	[M+H]+1	0.0072	0.0068
MET 442	257.13929	7.492	[M-H]-1	0.0813	0.0766
MET 443	500.31165	7.492	[M+H]+1	0.0024	0.0014
MET 444	278.06572	7.499	[M+H]+1	0.0014	0.0014
MET 445	319.15372	7.529	[M+H]+1	0.0038	0.0031
MET 446	380.21761	7.53	[M+H]+1	0.0077	0.0063
MET 447	541.33816	7.535	[M+H]+1	0.0059	0.0037
MET 448	363.14515	7.54	[M-H]-1	0.0124	0.0109
MET 449	365.19685	7.544	[M-H]-1	0.0009	0.0077
MET 450	553.3706	7.562	[M+H]+1	0.0013	0.0017
MET 451	146.05995	7.564	[M+H]+1	0.0962	0.0595
MET 452	287.01095	7.567	[M+H]+1	0.0152	0.0091
MET 453	379.13992	7.567	[M-H]-1	0.0468	0.0482
MET 454	164.52249	7.567	[M+2H]+2	0.0374	0.0250
MET 455	207.06555	7.572	[M-H]-1	0.0076	0.0080
MET 456	443.28617	7.578	[M+H]+1	0.0028	0.0026
MET 457	409.13898	7.595	[M+H]+1	0.0040	0.0028
MET 458	313.11793	7.65	[M+H]+1	0.0085	0.0064
MET 459	496.244	7.696	[M+H]+1	0.0036	0.0034
MET 460	248.09152	7.796	[M+H]+1	0.0082	0.0078
MET 461	392.21757	7.796	[M+H]+1	0.0037	0.0034
MET 462	287.18639	7.836	[M-H]-1	0.0557	0.0250
MET 463	253.17956	7.836	[M+H]+1	0.0189	0.0098
MET 464	236.12794	7.87	[M+H]+1	0.0079	0.0271
MET 465	365.1275	7.883	[M-H]-1	0.0016	0.0931
MET 466	361.0964	7.919	[M-H]-1	0.1170	0.0248
MET 467	425.18197	7.972	[M-H]-1	0.0106	0.0088

MET 468	379.17621	7.973	[M-H]-1	0.0230	0.0192
MET 469	339.07605	8.01	[M+H-H2O]+1	0.0072	0.0076
MET 470	340.08382	8.012	[M+H]+1	0.0320	0.0330
MET 471	342.04074	8.013	[M-H]-1	0.0146	0.0155
MET 472	243.12345	8.013	[M-H]-1	0.3977	0.4296
MET 473	540.16629	8.016	[M-H]-1	0.0015	0.0018
MET 474	573.23434	8.021	[M-H-H2O]-1	0.0038	0.0036
MET 475	392.09886	8.025	[M-H]-1	0.0091	0.0097
MET 476	475.28989	8.058	[M+H]+1	0.0017	0.0018
MET 477	471.22352	8.07	[M+H]+1	0.0038	0.0046
MET 478	135.1168	8.084	[M+H]+1	0.0457	0.0451
MET 479	326.10455	8.085	[M+H]+1	0.0254	0.0241
MET 480	195.13783	8.086	[M+H]+1	0.0669	0.0654
MET 481	177.12725	8.087	[M+H]+1	0.0094	0.0094
MET 482	229.14406	8.087	[M-H]-1	0.2892	0.2785
MET 483	209.04485	8.117	[M-H]-1	0.0201	0.0191
MET 484	162.05485	8.17	[M-H]-1	0.0106	0.0075
MET 485	164.07042	8.17	[M+H]+1	0.0397	0.0293
MET 486	317.13935	8.186	[M-H]-1	0.0091	0.0037
MET 487	256.11765	8.227	[M+H]+1	0.0167	0.0095
MET 488	177.16366	8.3	[M+H-H2O]+1	0.0049	0.0029
MET 489	425.1026	8.31	[M-H]-1	0.0120	0.0117
MET 490	227.12838	8.321	[M-H]-1	0.1475	0.1240
MET 491	477.29531	8.331	[M+H]+1	0.0027	0.0021
MET 492	283.20125	8.336	[M+H]+1	0.0069	0.0071
MET 493	363.15959	8.341	[M-H]-1	0.0132	0.0140
MET 494	338.06815	8.343	[M+H]+1	0.0111	0.0113
MET 495	241.10777	8.349	[M-H]-1	0.2008	0.1973
MET 496	477.27048	8.42	[M+H]+1	0.0385	0.0312
MET 497	173.08079	8.425	[M-H]-1	0.0821	0.1037
MET 498	270.04199	8.426	[M+H]+1	0.0078	0.0099
MET 499	257.13922	8.431	[M-H]-1	0.0273	0.0208
MET 500	379.22234	8.463	[M+H]+1	0.0039	0.0039
MET 501	429.20153	8.466	[M+H]+1	0.0043	0.0007
MET 502	404.99494	8.472	[M-H]-1	0.0179	0.0213
MET 503	305.06996	8.473	[M-H]-1	23.305	16.332
MET 504	327.05191	8.474	[M-H]-1	0.0547	0.0375
MET 505	402.03001	8.475	[M+H]+1	0.0292	0.0217
MET 506	303.04225	8.508	[M+H]+1	0.0529	0.0054
MET 507	397.18666	8.509	[M-H]-1	0.1205	0.0930
MET 508	345.16937	8.512	[M+H-H2O]+1	0.0028	0.0022
MET 509	165.05453	8.513	[M-H]-1	21.599	0.1034
MET 510	211.06042	8.516	[M-H]-1	0.3395	0.0099
MET 511	146.05995	8.518	[M+H]+1	0.0575	0.0538
MET 512	353.10056	8.533	[M-H]-1	0.0566	0.0020
MET 513	281.10645	8.539	[M-H]-1	0.0161	0.0116
MET 514	253.118	8.539	[M+H]+1	0.0270	0.0270
MET 515	361.1407	8.579	[M-H]-1	0.0059	0.0059
MET 516	266.13838	8.614	[M+H]+1	0.0083	0.0073
MET 517	222.07592	8.646	[M+H]+1	0.0094	0.0082
MET 518	162.05481	8.713	[M+H]+1	0.0310	0.0212
MET 519	254.01273	8.722	[M-H]-1	0.0068	0.0084
MET 520	206.08155	8.74	[M-H]-1	0.0051	0.0039
MET 521	404.04571	8.766	[M+H]+1	0.0036	0.0027
MET 522	307.08573	8.771	[M-H]-1	0.2645	0.2152
MET 523	423.10888	8.776	[M-H]-1	0.0195	0.0100
MET 524	469.11273	8.778	[M+H]+1	0.0173	0.0091
MET 525	513.10398	8.778	[M-H]-1	0.0151	0.0089
MET 526	567.02395	8.779	[M-H]-1	0.0029	0.0016

MET 527	535.08604	8.78	[M-H]-1	0.0046	0.0029
MET 528	612.0208	8.781	[M-H]-1	0.0024	0.0016
MET 529	365.16056	8.834	[M-H]-1	0.0045	0.0036
MET 530	235.10749	8.879	[M+H]+1	0.0634	0.0249
MET 531	238.10715	8.879	[M+H]+1	0.0075	0.0081
MET 532	260.02337	8.896	[M-H]-1	0.0061	0.0169
MET 533	191.08133	8.936	[M+H]+1	0.0003	0.0100
MET 534	351.21777	8.969	[M-H]-1	0.0087	0.0130
MET 535	239.0592	8.993	[M-H]-1	0.0136	0.0150
MET 536	331.11726	9.027	[M+H]+1	0.0154	0.0014
MET 537	348.16637	9.055	[M-H]-1	0.0178	0.0163
MET 538	275.02317	9.056	[M-H]-1	0.0463	0.0238
MET 539	285.17071	9.064	[M-H]-1	0.1130	0.1063
MET 540	239.16386	9.086	[M+H]+1	0.0222	0.0230
MET 541	299.14997	9.096	[M-H]-1	0.2097	0.2341
MET 542	601.32329	9.131	[M-H]-1	0.0915	0.0450
MET 543	245.01219	9.145	[M-H]-1	0.0181	0.0184
MET 544	221.15335	9.146	[M+H]+1	0.0722	0.0658
MET 545	175.14797	9.146	[M+H]+1	0.0111	0.0101
MET 546	159.11665	9.146	[M+H-H2O]+1	0.0548	0.0501
MET 547	119.08564	9.147	[M+H]+1	0.0191	0.0175
MET 548	203.14285	9.147	[M+H]+1	0.1278	0.1156
MET 549	159.11664	9.147	[M+H]+1	0.0548	0.0501
MET 550	202.06918	9.148	[M+2H]+2	0.0132	0.0119
MET 551	249.14816	9.148	[M+H]+1	0.0510	0.0478
MET 552	301.1655	9.148	[M-H]-1	20.976	18.750
MET 553	398.12551	9.149	[M+H]+1	0.0377	0.0323
MET 554	625.32076	9.149	[M-H]-1	0.0170	0.0136
MET 555	656.24988	9.149	[M-H]-1	0.0104	0.0089
MET 556	958.42322	9.149	[M-H]-1	0.0026	0.0021
MET 557	603.33888	9.149	[M-H]-1	0.2493	0.1943
MET 558	205.15844	9.15	[M+H]+1	0.0066	0.0060
MET 559	364.16144	9.15	[M-H]-1	0.0499	0.0451
MET 560	400.08251	9.15	[M-H]-1	0.0720	0.0659
MET 561	323.14738	9.15	[M-H]-1	0.0271	0.0246
MET 562	369.15279	9.151	[M-H]-1	0.0295	0.0284
MET 563	322.1533	9.152	[M+2H]+2	0.0022	0.0020
MET 564	180.07424	9.153	[M+2H]+2	0.0088	0.0076
MET 565	577.23255	9.164	[M-H]-1	0.0093	0.0174
MET 566	337.0388	9.173	[M-H]-1	0.0157	0.0143
MET 567	217.10749	9.184	[M-H]-1	0.0256	0.0208
MET 568	425.10256	9.198	[M-H]-1	0.0088	0.0082
MET 569	631.27636	9.2	[M-H]-1	0.0251	0.0224
MET 570	466.19793	9.214	[M-H]-1	0.0070	0.0062
MET 571	605.17017	9.222	[M-H]-1	0.0028	0.0064
MET 572	330.22699	9.229	[M+H]+1	0.2394	0.2177
MET 573	374.2184	9.235	[M-H]-1	0.0260	0.0238
MET 574	649.25219	9.25	[M-H]-1	0.0083	0.0036
MET 575	153.01807	9.255	[M-H]-1	0.0421	0.0390
MET 576	483.12974	9.258	[M-H]-1	0.0034	0.0046
MET 577	240.08742	9.297	[M-H]-1	0.0059	0.0063
MET 578	498.15377	9.337	[M+H]+1	0.0037	0.0042
MET 579	347.15007	9.35	[M-H]-1	0.0297	0.0295
MET 580	331.15363	9.352	[M+H]+1	0.0036	0.0038
MET 581	261.12307	9.371	[M+H]+1	0.0012	0.0020
MET 582	256.06482	9.403	[M-H]-1	0.0136	0.0294
MET 583	475.23727	9.463	[M-H]-1	0.0040	0.0062
MET 584	374.24341	9.493	[M+H]+1	0.0078	0.0037
MET 585	149.04483	9.582	[M+H-H2O]+1	0.0018	0.0020

MET 586	269.17596	9.626	[M-H]-1	0.0029	0.0047
MET 587	311.05626	9.668	[M-H]-1	0.0048	0.0044
MET 588	253.0829	9.678	[M-H]-1	0.0025	0.0018
MET 589	431.21102	9.684	[M-H]-1	0.0058	0.0025
MET 590	331.11894	9.726	[M-H]-1	0.0060	0.0082
MET 591	696.43727	9.738	[M+H]+1	0.0017	0.0011
MET 592	209.0813	9.753	[M-H]-1	0.0112	0.0066
MET 593	163.04087	9.771	[M+2H]+2	0.0020	0.0027
MET 594	148.07559	9.777	[M+H]+1	0.0879	0.1191
MET 595	489.30688	9.822	[M+H]+1	0.0200	0.0191
MET 596	547.27766	9.824	[M-H]-1	0.0121	0.0118
MET 597	297.06499	9.882	[M-H]-1	0.0066	0.0065
MET 598	254.01275	9.904	[M-H]-1	0.0071	0.0099
MET 599	255.19521	9.906	[M+H]+1	0.0070	0.0060
MET 600	271.1916	9.907	[M-H]-1	0.0430	0.0329
MET 601	323.08073	9.93	[M-H]-1	0.0183	0.0199
MET 602	367.21262	10.011	[M-H]-1	0.0503	0.0441
MET 603	527.23603	10.011	[M-H]-1	0.0017	0.0015
MET 604	363.18162	10.022	[M-H]-1	0.0275	0.0239
MET 605	408.16985	10.092	[M-H]-1	0.0047	0.0042
MET 606	365.27954	10.128	[M+H]+1	0.0033	0.0021
MET 607	363.14526	10.138	[M-H]-1	0.0099	0.0098
MET 608	470.26444	10.148	[M+H]+1	0.0010	0.0016
MET 609	271.06127	10.148	[M-H]-1	0.0034	0.0025
MET 610	243.08743	10.17	[M+H]+1	0.0035	0.0047
MET 611	360.15506	10.188	[M+H]+1	0.0024	0.0031
MET 612	573.18798	10.287	[M-H]-1	0.0024	0.0022
MET 613	443.2536	10.287	[M+H]+1	0.0028	0.0029
MET 614	233.15341	10.316	[M+H-H2O]+1	0.0057	0.0069
MET 615	542.28586	10.318	[M+H]+1	0.0011	0.0023
MET 616	209.07884	10.324	[M-H]-1	0.0066	0.0096
MET 617	287.02249	10.324	[M-H]-1	0.0043	0.0065
MET 618	295.12203	10.334	[M-H]-1	0.0150	0.0142
MET 619	278.10671	10.352	[M-H]-1	0.0067	0.0077
MET 620	457.23258	10.365	[M+H]+1	0.0028	0.0025
MET 621	392.099	10.395	[M-H]-1	0.0129	0.0090
MET 622	361.09647	10.398	[M-H]-1	0.0226	0.0225
MET 623	365.0797	10.398	[M-H]-1	0.0042	0.0030
MET 624	484.28025	10.4	[M+H]+1	0.0064	0.0057
MET 625	176.07048	10.431	[M+H]+1	0.1215	0.1550
MET 626	130.06516	10.431	[M+H]+1	0.0244	0.0311
MET 627	300.21656	10.433	[M+H]+1	0.0066	0.0050
MET 628	312.04261	10.433	[M+H]+1	0.0065	0.0079
MET 629	392.13356	10.434	[M+NH4]+1	0.0070	0.0115
MET 630	374.24344	10.525	[M+H]+1	0.0101	0.0057
MET 631	332.04134	10.548	[M-H]-1	0.0099	0.0099
MET 632	271.05976	10.55	[M+H]+1	0.0564	0.0583
MET 633	343.26272	10.594	[M+H]+1	0.0017	0.0003
MET 634	242.99661	10.604	[M-H]-1	0.0436	0.0481
MET 635	427.13087	10.636	[M-H]-1	0.0154	0.0080
MET 636	347.15014	10.768	[M-H]-1	0.0079	0.0082
MET 637	336.07274	10.834	[M-H]-1	0.0056	0.0047
MET 638	273.07698	10.835	[M-H]-1	0.0097	0.0079
MET 639	453.2032	10.838	[M-H]-1	0.0040	0.0038
MET 640	593.33299	10.86	[M+H]+1	0.0366	0.0872
MET 641	267.16021	10.887	[M-H]-1	0.0301	0.0160
MET 642	591.31722	10.96	[M+H]+1	0.1416	0.0916
MET 643	289.10826	10.985	[M-H]-1	0.0047	0.0081
MET 644	297.07678	11.011	[M-H]-1	0.0367	0.0302

MET 645	360.07272	11.012	[M-H]-1	0.0162	0.0135
MET 646	333.05372	11.014	[M-H]-1	0.0018	0.0013
MET 647	473.22144	11.196	[M-H]-1	0.0092	0.0160
MET 648	415.14001	11.243	[M-H]-1	0.0068	0.0047
MET 649	261.11185	11.253	[M+H]+1	0.0060	0.0060
MET 650	263.09588	11.263	[M-H]-1	0.0061	0.0051
MET 651	232.1364	11.315	[M+H]+1	0.0016	0.0017
MET 652	289.07185	11.333	[M-H]-1	0.0039	0.0042
MET 653	315.124	11.421	[M-H]-1	0.0022	0.0172
MET 654	251.05933	11.435	[M-H]-1	0.0062	0.0064
MET 655	367.22227	11.443	[M+H]+1	0.0011	0.0011
MET 656	636.3978	11.506	[M+2H]+2	0.0053	0.0018
MET 657	253.05038	11.664	[M-H]-1	0.0269	0.0665
MET 658	291.12736	11.666	[M-H]-1	0.0064	0.0053
MET 659	255.11375	11.67	[M-H]-1	0.0041	0.0017
MET 660	333.14409	11.674	[M+H]+1	0.0063	0.0027
MET 661	319.16635	11.779	[M-H]-1	0.0068	0.0127
MET 662	349.20209	11.792	[M-H]-1	0.0103	0.0093
MET 663	318.16983	11.815	[M+H]+1	0.0022	0.0019
MET 664	285.14815	11.83	[M+H]+1	0.0026	0.0012
MET 665	301.14448	11.834	[M-H]-1	0.0134	0.0063
MET 666	364.14041	11.837	[M-H]-1	0.0068	0.0033
MET 667	313.16581	11.886	[M-H]-1	0.0168	0.0190
MET 668	237.11284	11.904	[M-H]-1	0.0208	0.0157
MET 669	381.31076	11.914	[M+H]+1	0.0064	0.0063
MET 670	257.08177	11.921	[M-H-H2O]-1	0.0092	0.0082
MET 671	232.0966	11.932	[M+H]+1	0.0120	0.0114
MET 672	262.1084	11.933	[M-H]-1	0.0102	0.0094
MET 673	193.08615	11.951	[M-H]-1	0.0615	0.0451
MET 674	223.10043	11.96	[M-H]-1	0.0077	0.0077
MET 675	363.14517	12.056	[M-H]-1	0.0041	0.0044
MET 676	722.39513	12.124	[M+H]+1	0.0053	0.0052
MET 677	263.09582	12.125	[M-H]-1	0.0306	0.0256
MET 678	313.1083	12.178	[M-H]-1	0.0193	0.0187
MET 679	377.16073	12.244	[M-H]-1	0.0104	0.0109
MET 680	375.18469	12.33	[M-H]-1	0.0064	0.0141
MET 681	299.05204	12.341	[M-H]-1	0.0066	0.0072
MET 682	194.08109	12.344	[M+H]+1	0.0063	0.0073
MET 683	190.08612	12.345	[M+H]+1	0.0131	0.0165
MET 684	248.9858	12.369	[M-H]-1	0.0068	0.0099
MET 685	395.08109	12.371	[M+H-H2O]+1	0.0017	0.0031
MET 686	382.05152	12.371	[M-H]-1	0.0043	0.0070
MET 687	287.04741	12.372	[M+H]+1	0.0076	0.0130
MET 688	332.045	12.374	[M+H]+1	0.0031	0.0053
MET 689	309.08657	12.456	[M+H]+1	0.0035	0.0030
MET 690	196.00654	12.498	[M-H]-1	0.0800	0.0775
MET 691	358.25837	12.507	[M+H]+1	0.0281	0.0404
MET 692	429.0279	12.543	[M-H]-1	0.0059	0.0035
MET 693	329.10312	12.544	[M-H]-1	0.1795	0.0969
MET 694	351.085	12.544	[M-H]-1	0.0108	0.0060
MET 695	467.08972	12.545	[M+H]+1	0.0047	0.0025
MET 696	392.09904	12.546	[M-H]-1	0.0046	0.0025
MET 697	322.16448	12.564	[M+H]+1	0.0193	0.0113
MET 698	413.20045	12.564	[M-H]-1	0.0240	0.0158
MET 699	335.22284	12.572	[M-H]-1	0.0044	0.0037
MET 700	363.14513	12.597	[M-H]-1	0.0420	0.0349
MET 701	415.2162	12.732	[M-H]-1	0.0024	0.0058
MET 702	386.12813	12.78	[M-H]-1	0.0053	0.0060
MET 703	370.133	12.789	[M-H]-1	0.0081	0.0088

MET 704	411.06347	12.892	[M+H]+1	0.0025	0.0026
MET 705	547.16131	12.893	[M-H]-1	0.0157	0.0184
MET 706	273.07689	12.894	[M-H]-1	0.2825	0.3202
MET 707	336.07263	12.895	[M-H]-1	0.0316	0.0369
MET 708	371.99435	12.896	[M-H]-1	0.0043	0.0045
MET 709	287.05614	12.898	[M-H-H2O]-1	0.0053	0.0058
MET 710	341.06418	12.899	[M-H]-1	0.0076	0.0087
MET 711	257.08181	12.991	[M-H]-1	0.0199	0.0204
MET 712	279.12596	13.074	[M+H]+1	0.0038	0.0022
MET 713	473.22155	13.113	[M-H]-1	0.0113	0.0150
MET 714	448.23603	13.276	[M+H]+1	0.0164	0.0155
MET 715	452.31149	13.341	[M+H]+1	0.0048	0.0044
MET 716	251.1287	13.426	[M-H]-1	0.0059	0.0122
MET 717	475.23722	13.489	[M-H]-1	0.0031	0.0046
MET 718	379.2951	13.498	[M+H]+1	0.0198	0.0181
MET 719	336.1488	13.709	[M-H]-1	0.0126	0.0130
MET 720	257.08054	13.757	[M+H]+1	0.0018	0.0014
MET 721	243.10141	13.759	[M+H]+1	0.0086	0.0062
MET 722	370.01506	13.763	[M-H]-1	0.0046	0.0034
MET 723	459.2424	13.857	[M-H]-1	0.0036	0.0059
MET 724	267.09078	13.909	[M-H]-1	0.0074	0.0282
MET 725	414.12297	13.935	[M-H]-1	0.0152	0.0123
MET 726	207.1014	14.091	[M+H]+1	0.0213	0.0236
MET 727	227.12838	14.138	[M-H]-1	0.0565	0.0380
MET 728	343.11865	14.149	[M-H]-1	0.0168	0.0266
MET 729	218.05356	14.15	[M+2H]+2	0.0021	0.0029
MET 730	297.11309	14.15	[M-H]-1	0.0750	0.1125
MET 731	415.0492	14.15	[M-H]-1	0.0025	0.0032
MET 732	333.09001	14.15	[M-H]-1	0.0191	0.0287
MET 733	360.10902	14.151	[M-H]-1	0.0742	0.1126
MET 734	685.36299	14.179	[M-H]-1	0.0040	0.0043
MET 735	347.15005	14.229	[M-H]-1	0.0048	0.0042
MET 736	471.18343	14.275	[M+Na]+1	0.0126	0.0023
MET 737	514.28426	14.308	[M-H]-1	0.0066	0.0091
MET 738	279.1223	14.386	[M+H]+1	0.0064	0.0071
MET 739	360.18517	14.428	[M-H]-1	0.0028	0.0047
MET 740	977.49541	14.611	[M+H]+1	0.0008	0.0007
MET 741	487.237	14.611	[M-H]-1	0.7297	0.8002
MET 742	373.27323	14.611	[M+H]+1	0.0200	0.0231
MET 743	560.25374	14.612	[M-H]-1	0.0048	0.0055
MET 744	587.16226	14.612	[M-H]-1	0.0086	0.0096
MET 745	509.21874	14.613	[M-H]-1	0.0254	0.0294
MET 746	584.19695	14.613	[M+H]+1	0.0073	0.0078
MET 747	568.22464	14.618	[M+H]+1	0.0029	0.0033
MET 748	345.13763	14.639	[M-H]-1	0.0034	0.0073
MET 749	469.22646	14.663	[M-H]-1	0.0167	0.0184
MET 750	274.27366	14.682	[M+H]+1	0.0989	0.0858
MET 751	457.22672	14.689	[M-H]-1	0.0179	0.0376
MET 752	342.26341	14.715	[M+H]+1	0.0023	0.0046
MET 753	553.26681	14.744	[M-H]-1	0.0030	0.0045
MET 754	357.06169	14.824	[M-H]-1	0.0109	0.0108
MET 755	318.29983	14.853	[M+H]+1	0.0692	0.0627
MET 756	401.08773	14.907	[M-H]-1	0.0254	0.0260
MET 757	257.08173	14.938	[M-H]-1	0.0173	0.0282
MET 758	362.32596	14.987	[M+H]+1	0.0192	0.0179
MET 759	491.11961	14.994	[M-H]-1	0.0095	0.0109
MET 760	469.11012	14.998	[M+Na]+1	0.0054	0.0055
MET 761	505.27442	15.018	[M+H]+1	0.0047	0.0035
MET 762	729.40905	15.018	[M+H]+1	0.0020	0.0014

MET 763	167.14289	15.019	[M+H]+1	0.0532	0.0449
MET 764	459.29651	15.019	[M-H]-1	0.0254	0.0175
MET 765	512.20737	15.019	[M-H]-1	0.0105	0.0079
MET 766	229.14401	15.019	[M-H]-1	22.954	18.642
MET 767	481.27838	15.019	[M-H]-1	0.0362	0.0260
MET 768	499.25713	15.019	[M+H]+1	0.0025	0.0021
MET 769	251.12611	15.02	[M-H]-1	0.0823	0.0728
MET 770	270.64548	15.02	[M+2H]+2	0.0013	0.0010
MET 771	367.13102	15.02	[M+H]+1	0.0205	0.0164
MET 772	185.08838	15.02	[M+2H]+2	0.0035	0.0029
MET 773	362.12876	15.021	[M+H]+1	0.0044	0.0037
MET 774	176.08304	15.021	[M+2H]+2	0.0101	0.0090
MET 775	326.10448	15.021	[M+H]+1	0.0353	0.0281
MET 776	745.38143	15.021	[M+H]+1	0.0013	0.0008
MET 777	196.59633	15.022	[M+2H]+2	0.0038	0.0032
MET 778	329.06951	15.022	[M-H]-1	0.0301	0.0268
MET 779	328.1044	15.022	[M+H]+1	0.0031	0.0027
MET 780	366.12324	15.024	[M+H-H2O]+1	0.0046	0.0040
MET 781	168.09422	15.024	[M+2H]+2	0.0025	0.0022
MET 782	312.06711	15.027	[M-H]-1	0.0224	0.0191
MET 783	328.06201	15.028	[M-H]-1	0.0285	0.0251
MET 784	429.19508	15.049	[M-H]-1	0.0483	0.0552
MET 785	406.35221	15.07	[M+H]+1	0.0051	0.0045
MET 786	293.14283	15.074	[M-H]-1	0.0158	0.0211
MET 787	385.16918	15.213	[M-H]-1	0.0132	0.0084
MET 788	349.20205	15.239	[M-H]-1	0.0566	0.0542
MET 789	204.04916	15.409	[M+2H]+2	0.0082	0.0092
MET 790	252.04261	15.409	[M-H]-1	0.0325	0.0334
MET 791	412.99654	15.409	[M-H]-1	0.0350	0.0349
MET 793	195.04391	15.41	[M+2H]+2	0.0205	0.0218
MET 794	203.02999	15.41	[M+2H]+2	0.0667	0.0643
MET 795	330.06184	15.41	[M-H]-1	0.0777	0.0808
MET 796	335.05371	15.41	[M-H]-1	0.0235	0.0251
MET 797	535.13979	15.41	[M-H]-1	0.6004	0.6101
MET 798	296.03992	15.411	[M+2H]+2	0.0204	0.0190
MET 799	422.09036	15.411	[M+2H]+2	0.0051	0.0052
MET 800	637.07831	15.411	[M+H]+1	0.0069	0.0065
MET 801	430.07663	15.411	[M+2H]+2	0.0113	0.0105
MET 802	618.06225	15.412	[M-H]-1	0.0301	0.0309
MET 803	564.11354	15.412	[M+2H]+2	0.0062	0.0051
MET 804	303.04285	15.413	[M-H]-1	0.0376	0.0378
MET 805	215.55706	15.413	[M+2H]+2	0.0071	0.0066
MET 806	369.00497	15.413	[M+H]+1	0.0255	0.0240
MET 807	332.08862	15.414	[M+H]+1	0.0174	0.0178
MET 808	288.05384	15.414	[M+2H]+2	0.0094	0.0096
MET 809	410.03148	15.421	[M+H]+1	0.0054	0.0048
MET 810	415.21593	15.477	[M-H]-1	0.0091	0.0383
MET 811	410.2894	15.481	[M+H]+1	0.0207	0.0138
MET 812	331.19154	15.628	[M-H]-1	0.0113	0.0129
MET 813	297.18446	15.639	[M+H-H2O]+1	0.0046	0.0053
MET 814	293.10654	15.656	[M-H]-1	0.0019	0.0193
MET 815	253.14439	15.669	[M-H]-1	0.0237	0.0193
MET 816	345.16914	15.692	[M+H]+1	0.0014	0.0044
MET 817	331.18988	15.789	[M+H]+1	0.0067	0.0089
MET 818	334.07571	15.878	[M-H]-1	0.0035	0.0031
MET 819	437.30074	15.985	[M+H]+1	0.0042	0.0038
MET 820	297.04723	16.016	[M-H-H2O]-1	0.0020	0.0024
MET 821	490.25627	16.035	[M-H]-1	0.0029	0.0022
MET 822	807.33014	16.046	[M-H]-1	0.0029	0.0027

MET 823	487.30676	16.203	[M-H]-1	0.0030	0.0115
MET 824	533.31245	16.204	[M-H]-1	0.0034	0.0104
MET 825	393.26504	16.24	[M-H]-1	0.0014	0.0030
MET 826	550.38475	16.263	[M+H]+1	0.0004	0.0011
MET 827	135.08045	16.284	[M+H]+1	0.0216	0.0184
MET 828	653.26231	16.295	[M-H]-1	0.0032	0.0040
MET 829	502.27992	16.341	[M+H]+1	0.0009	0.0008
MET 830	267.16027	16.375	[M-H]-1	0.0128	0.0144
MET 831	429.11919	16.417	[M-H]-1	0.0033	0.0033
MET 832	338.98477	16.433	[M-H]-1	0.0014	0.0015
MET 833	321.98229	16.436	[M-H]-1	0.0029	0.0029
MET 834	383.98242	16.436	[M-H]-1	0.0025	0.0029
MET 835	223.06351	16.438	[M+H]+1	0.0065	0.0072
MET 836	1273.58554	16.441	[M-H]-1	0.0017	0.0026
MET 837	519.15094	16.473	[M-H]-1	0.0031	0.0033
MET 838	362.19761	16.49	[M-H]-1	0.0044	0.0024
MET 839	428.26535	16.496	[M-H]-1	0.0015	0.0022
MET 840	321.20717	16.556	[M-H]-1	0.0020	0.0027
MET 841	385.09315	16.605	[M-H]-1	0.0077	0.0077
MET 842	480.35285	16.627	[M+H]+1	0.0109	0.0087
MET 843	429.11913	16.685	[M-H]-1	0.0116	0.0117
MET 844	497.14147	16.769	[M+Na]+1	0.0039	0.0040
MET 845	558.3844	16.769	[M+H]+1	0.0021	0.0016
MET 846	442.19064	16.797	[M-H]-1	0.0118	0.0095
MET 847	255.16004	16.862	[M-H]-1	0.1112	0.1058
MET 848	277.14201	16.862	[M-H]-1	0.0062	0.0060
MET 849	352.12033	16.865	[M+H]+1	0.0014	0.0013
MET 850	354.07773	16.868	[M-H]-1	0.0015	0.0015
MET 851	893.36676	16.949	[M-H]-1	0.0064	0.0057
MET 852	329.31587	17.021	[M+H]+1	0.0039	0.0048
MET 853	318.29988	17.104	[M+H]+1	0.0096	0.0080
MET 854	441.15902	17.115	[M-H]-1	0.0073	0.0082
MET 855	283.09613	17.144	[M+H]+1	0.0510	0.0526
MET 856	399.18471	17.151	[M-H]-1	0.0126	0.0137
MET 857	522.34229	17.166	[M+H]+1	0.0005	0.0032
MET 858	401.16399	17.189	[M-H]-1	0.0708	0.0770
MET 859	259.1914	17.207	[M-H]-1	0.0033	0.0044
MET 860	517.3171	17.212	[M-H]-1	0.0250	0.0080
MET 861	563.32278	17.214	[M-H]-1	0.0041	0.0013
MET 862	514.28425	17.275	[M-H]-1	0.0197	0.0356
MET 863	943.52567	17.288	[M+H]+1	0.0176	0.0225
MET 864	987.51745	17.288	[M-H]-1	0.0416	0.0541
MET 865	1009.49914	17.294	[M-H]-1	0.0034	0.0041
MET 866	219.18704	17.358	[M+H]+1	0.0680	0.0567
MET 867	501.32241	17.455	[M-H]-1	0.0044	0.0118
MET 868	470.18544	17.491	[M-H]-1	0.0040	0.0044
MET 869	397.16894	17.497	[M-H]-1	0.0904	0.0958
MET 870	180.03791	17.515	[M+2H]+2	0.0051	0.0022
MET 871	223.0963	17.518	[M+H]+1	0.1130	0.0528
MET 872	177.0545	17.518	[M+H]+1	0.0938	0.0432
MET 873	485.29114	17.535	[M-H]-1	0.0023	0.0078
MET 874	464.35787	17.577	[M+H]+1	0.0231	0.0198
MET 875	508.34916	17.578	[M-H]-1	0.0123	0.0104
MET 876	677.35455	17.587	[M-H]-1	0.0049	0.0038
MET 877	455.24756	17.641	[M-H]-1	0.0055	0.0076
MET 878	337.13285	17.681	[M-H]-1	0.0172	0.0181
MET 879	300.28934	17.701	[M+H]+1	0.0695	0.0635
MET 880	957.50684	17.733	[M-H]-1	0.0181	0.0232
MET 881	911.49964	17.733	[M-H]-1	0.0082	0.0106

MET 882	979.40359	17.768	[M-H]-1	0.0063	0.0045
MET 883	271.01683	17.79	[M-H]-1	0.0010	0.0010
MET 884	399.18469	17.799	[M-H]-1	0.0181	0.0204
MET 885	380.32676	17.828	[M+H]+1	0.0013	0.0035
MET 886	795.45394	17.838	[M-H]-1	0.0070	0.0126
MET 887	257.17571	17.847	[M-H]-1	0.0755	0.0841
MET 888	279.1577	17.847	[M-H]-1	0.0046	0.0051
MET 889	269.17588	17.882	[M-H]-1	0.0141	0.0117
MET 890	473.32727	18.004	[M-H]-1	0.0109	0.0255
MET 891	519.33287	18.007	[M-H]-1	0.0070	0.0154
MET 892	457.15054	18.128	[M-H]-1	0.0044	0.0045
MET 893	765.44347	18.131	[M-H]-1	0.0040	0.0047
MET 894	515.30181	18.138	[M-H]-1	0.0084	0.0018
MET 895	503.1908	18.184	[M+H]+1	0.0037	0.0040
MET 896	302.30498	18.206	[M+H]+1	0.0342	0.0332
MET 897	393.1955	18.353	[M-H]-1	0.3472	0.3682
MET 898	583.29483	18.364	[M-H]-1	0.0076	0.0204
MET 899	368.30035	18.491	[M+NH4]+1	0.0011	0.0012
MET 900	1065.44011	18.496	[M-H]-1	0.0041	0.0023
MET 901	412.32654	18.604	[M+H]+1	0.0014	0.0016
MET 902	473.32723	18.644	[M-H]-1	0.0050	0.0137
MET 903	314.30493	18.675	[M+H]+1	0.0037	0.0037
MET 904	499.30674	18.725	[M-H]-1	0.0068	0.0231
MET 905	545.31266	18.728	[M-H]-1	0.0012	0.0049
MET 906	589.37499	18.731	[M-H]-1	0.0013	0.0012
MET 907	482.34728	18.775	[M+H]+1	0.0023	0.0027
MET 908	549.1042	18.776	[M-H]-1	0.0055	0.0057
MET 909	421.31701	18.779	[M+H]+1	0.0015	0.0018
MET 910	420.25069	18.78	[M-H]-1	0.0108	0.0138
MET 911	393.28559	18.78	[M+NH4]+1	0.0682	0.0895
MET 912	407.30127	18.781	[M+H]+1	0.0018	0.0022
MET 913	544.40507	18.785	[M+H]+1	0.0014	0.0013
MET 914	588.43149	18.835	[M+H]+1	0.0011	0.0013
MET 915	1113.54922	18.841	[M-H]-1	0.0054	0.0047
MET 916	1069.5576	18.841	[M+H]+1	0.0025	0.0021
MET 917	593.13054	18.858	[M-H]-1	0.0099	0.0104
MET 918	341.35229	18.869	[M+H]+1	0.0034	0.0057
MET 919	632.4574	18.882	[M+H]+1	0.0010	0.0011
MET 920	656.19708	18.896	[M+NH4]+1	0.0016	0.0018
MET 921	683.1623	18.897	[M-H]-1	0.0045	0.0049
MET 922	451.09962	19.012	[M+Na]+1	0.0103	0.0106
MET 923	346.33116	19.027	[M+H]+1	0.0114	0.0106
MET 924	519.18801	19.028	[M+Na]+1	0.0012	0.0012
MET 925	369.38356	19.041	[M+H]+1	0.0132	0.0138
MET 926	459.38061	19.046	[M-H]-1	0.0015	0.0015
MET 927	357.34716	19.05	[M+H]+1	0.0018	0.0021
MET 928	297.1118	19.053	[M+H]+1	0.0070	0.0070
MET 929	371.18981	19.114	[M-H]-1	0.0039	0.0029
MET 930	183.08036	19.134	[M+H]+1	0.0202	0.0148
MET 931	537.32581	19.154	[M-H]-1	0.0142	0.0134
MET 932	129.12745	19.218	[M+H]+1	0.0068	0.0070
MET 933	1083.53863	19.221	[M-H]-1	0.0023	0.0018
MET 934	425.34098	19.283	[M+H]+1	0.0075	0.0065
MET 935	356.15389	19.285	[M-H]-1	0.0297	0.0208
MET 936	505.35385	19.288	[M-H]-1	0.0151	0.0130
MET 937	293.28355	19.36	[M+H]+1	0.0035	0.0031
MET 938	471.2422	19.368	[M-H]-1	0.0221	0.0227
MET 939	387.19257	19.459	[M+H]+1	0.0070	0.0050
MET 940	431.1839	19.459	[M-H]-1	0.0121	0.0089

MET 941	451.21618	19.476	[M-H]-1	0.0461	0.0436
MET 942	565.28425	19.522	[M-H]-1	0.0209	0.0595
MET 943	328.32062	19.643	[M+H]+1	0.0486	0.0515
MET 944	286.31009	19.666	[M+H]+1	0.0057	0.0078
MET 945	742.52461	19.704	[M+H]+1	0.0030	0.0029
MET 946	446.25316	19.75	[M+H]+1	0.0317	0.0283
MET 947	867.37105	19.753	[M+H]+1	0.0056	0.0048
MET 948	846.44174	19.753	[M+H]+1	0.0131	0.0111
MET 949	851.39702	19.754	[M+H]+1	0.0107	0.0091
MET 950	432.23768	19.754	[M+NH4]+1	0.3908	0.3573
MET 951	494.25845	19.784	[M-H]-1	0.0056	0.0029
MET 952	199.1691	19.816	[M+H-H2O]+1	0.0180	0.0191
MET 953	503.33641	19.857	[M+H]+1	0.0113	0.0089
MET 954	547.32802	19.858	[M-H]-1	0.0156	0.0118
MET 955	569.31004	19.86	[M-H]-1	0.0066	0.0055
MET 956	482.20644	19.904	[M+NH4]+1	0.0124	0.0105
MET 957	711.19373	19.997	[M-H]-1	0.0011	0.0012
MET 958	316.32061	20.037	[M+H]+1	0.0376	0.0427
MET 959	291.26788	20.082	[M+H]+1	0.0103	0.0094
MET 960	349.2709	20.083	[M+Na]+1	0.0045	0.0041
MET 961	330.33625	20.147	[M+H]+1	0.0132	0.0120
MET 962	415.2111	20.148	[M+H]+1	0.0156	0.0148
MET 963	515.2684	20.169	[M-H]-1	0.0034	0.0110
MET 964	399.18464	20.223	[M-H]-1	0.0963	0.0884
MET 965	377.26982	20.229	[M-H]-1	0.0018	0.0108
MET 966	581.2793	20.238	[M-H]-1	0.0052	0.0138
MET 967	577.13544	20.243	[M-H]-1	0.0028	0.0031
MET 968	621.16175	20.328	[M-H]-1	0.0049	0.0052
MET 969	424.24721	20.349	[M-H]-1	0.0016	0.0183
MET 970	563.26852	20.356	[M-H]-1	0.0141	0.0303
MET 971	553.28436	20.391	[M-H]-1	0.0133	0.0472
MET 972	253.07126	20.463	[M+H]+1	0.0050	0.0030
MET 973	374.29122	20.492	[M-H]-1	0.0033	0.0030
MET 974	342.33624	20.494	[M+H]+1	0.0153	0.0171
MET 975	443.21098	20.503	[M-H]-1	0.0151	0.0134
MET 976	499.30691	20.539	[M-H]-1	0.0206	0.0128
MET 977	545.31256	20.539	[M-H]-1	0.0048	0.0029
MET 978	455.31704	20.541	[M-H]-1	0.0053	0.0033
MET 979	429.19516	20.654	[M-H]-1	0.0832	0.0816
MET 980	255.08691	20.744	[M+H]+1	0.0056	0.0045
MET 981	498.28952	20.767	[M-H]-1	0.1339	0.2989
MET 982	358.36749	20.776	[M+H]+1	0.0035	0.0036
MET 983	376.17508	20.793	[M+NH4]+1	0.0054	0.0096
MET 984	375.18469	20.834	[M-H]-1	0.5002	0.4262
MET 985	479.13096	20.837	[M+Na]+1	0.0069	0.0071
MET 986	877.3715	20.838	[M-H]-1	0.0023	0.0016
MET 987	297.11176	20.89	[M+H]+1	0.0291	0.0309
MET 988	219.19529	21.014	[M+H]+1	0.0068	0.0081
MET 989	397.41483	21.025	[M+H]+1	0.0264	0.0280
MET 990	471.3115	21.103	[M-H]-1	0.0033	0.0103
MET 991	399.18456	21.114	[M-H]-1	0.0128	0.0117
MET 992	315.25393	21.121	[M-H]-1	0.0257	0.0336
MET 993	496.27407	21.202	[M-H]-1	0.0069	0.0050
MET 994	383.39912	21.213	[M+H]+1	0.0073	0.0079
MET 995	467.2109	21.313	[M-H]-1	0.0108	0.0087
MET 996	409.28057	21.331	[M+NH4]+1	0.0021	0.0026
MET 997	741.14685	21.417	[M-H]-1	0.0012	0.0012
MET 998	459.3482	21.458	[M-H]-1	0.0016	0.0015
MET 999	350.26846	21.462	[M+H]+1	0.0015	0.0012

MET 1000	785.17289	21.519	[M-H]-1	0.0021	0.0020
MET 1001	356.3519	21.571	[M+H]+1	0.0031	0.0034
MET 1002	476.27828	21.665	[M-H]-1	0.0044	0.0044
MET 1003	963.40828	21.728	[M-H]-1	0.0028	0.0020
MET 1004	438.26259	21.752	[M-H]-1	0.0049	0.0637
MET 1005	427.21598	21.951	[M-H]-1	0.0081	0.0087
MET 1006	344.35185	21.995	[M+H]+1	0.0215	0.0235
MET 1007	359.18991	22.087	[M-H]-1	0.0379	0.0355
MET 1008	476.27821	22.131	[M-H]-1	0.0066	0.0091
MET 1009	520.33931	22.187	[M+H]+1	0.0049	0.0045
MET 1010	394.27701	22.321	[M+H]+1	0.0067	0.0059
MET 1011	372.31192	22.364	[M-H]-1	0.0033	0.0028
MET 1012	371.25774	22.653	[M+H]+1	0.0024	0.0079
MET 1013	520.33946	22.677	[M+H]+1	0.0055	0.0135
MET 1014	564.33074	22.677	[M-H]-1	0.0070	0.0173
MET 1015	349.23844	22.754	[M-H]-1	0.0037	0.0023
MET 1016	981.50644	22.787	[M-H]-1	0.0029	0.0014
MET 1017	501.32251	22.896	[M-H]-1	0.0055	0.0061
MET 1018	455.31671	22.907	[M-H]-1	0.0048	0.0047
MET 1019	551.26862	22.996	[M-H]-1	0.0206	0.0446
MET 1020	226.21631	23.056	[M+H]+1	0.0136	0.0106
MET 1021	517.31706	23.091	[M-H]-1	0.0024	0.0109
MET 1022	471.31147	23.094	[M-H]-1	0.0315	0.1376
MET 1023	468.38325	23.103	[M+H]+1	0.0162	0.0102
MET 1024	427.35665	23.104	[M+H]+1	0.0663	0.0462
MET 1025	452.27831	23.21	[M-H]-1	0.0400	0.1124
MET 1026	520.26549	23.212	[M-H]-1	0.0022	0.0066
MET 1027	1154.52194	23.262	[M+NH4]+1	0.0085	0.0074
MET 1028	471.24206	23.449	[M-H]-1	0.1667	0.1799
MET 1029	399.18458	23.54	[M-H]-1	0.0496	0.0632
MET 1030	401.20032	23.738	[M-H]-1	0.0058	0.0071
MET 1031	359.29402	23.931	[M+H]+1	0.0030	0.0038
MET 1032	1221.51783	23.933	[M-H]-1	0.0017	0.0012
MET 1033	496.33938	23.938	[M+H]+1	0.0552	0.0637
MET 1034	540.33077	23.938	[M-H]-1	0.0742	0.0837
MET 1035	608.31841	23.939	[M-H]-1	0.0041	0.0048
MET 1036	478.29389	23.943	[M-H]-1	0.0297	0.0321
MET 1037	493.30233	23.98	[M-H]-1	0.0088	0.0061
MET 1038	503.33807	24.021	[M-H]-1	0.0034	0.0057
MET 1039	566.34654	24.141	[M-H]-1	0.0088	0.0088
MET 1040	566.34645	24.691	[M-H]-1	0.0179	0.0227
MET 1041	522.3551	24.691	[M+H]+1	0.0159	0.0193
MET 1042	418.33095	24.778	[M+H]+1	0.0039	0.0039
MET 1043	751.58859	24.778	[M-H]-1	0.0031	0.0023
MET 1044	359.294	24.778	[M+H]+1	0.0266	0.0288
MET 1045	421.29618	24.779	[M-H]-1	0.0296	0.0309
MET 1046	391.10771	24.899	[M+H]+1	0.0028	0.0027
MET 1047	702.49917	24.989	[M+H]+1	0.0042	0.0043
MET 1048	658.47303	25.043	[M+H]+1	0.0065	0.0064
MET 1049	614.44668	25.099	[M+H]+1	0.0086	0.0084
MET 1050	622.19138	25.102	[M+NH4]+1	0.0065	0.0063
MET 1051	570.42041	25.153	[M+H]+1	0.0095	0.0089
MET 1052	482.35993	25.163	[M+H]+1	0.0114	0.0186
MET 1053	526.39432	25.209	[M+H]+1	0.0080	0.0074
MET 1054	425.3408	25.211	[M+H]+1	0.0415	0.0247
MET 1055	411.36167	25.256	[M+H]+1	0.3852	0.1581
MET 1056	377.26971	25.305	[M-H]-1	0.0098	0.0073
MET 1057	303.70027	25.478	[M+2H]+2	0.0036	0.0024
MET 1058	409.34603	25.483	[M+H]+1	0.0511	0.0303

MET 1059	468.38331	25.488	[M+H]+1	0.0114	0.0063
MET 1060	228.23195	25.505	[M+H]+1	0.0707	0.0515
MET 1061	545.33336	25.695	[M-H]-1	0.0084	0.0076
MET 1062	543.36943	25.825	[M-H]-1	0.0046	0.0037
MET 1063	324.28925	25.843	[M+H]+1	0.0093	0.0099
MET 1064	444.18156	25.843	[M-H]-1	0.0025	0.0028
MET 1065	265.25228	25.844	[M+H]+1	0.0186	0.0192
MET 1066	299.25902	25.846	[M-H]-1	0.3874	0.4064
MET 1067	367.24644	25.846	[M-H]-1	0.0195	0.0222
MET 1068	399.18428	25.847	[M-H]-1	0.0147	0.0151
MET 1069	345.26457	25.847	[M-H]-1	0.0098	0.0100
MET 1070	283.26276	25.848	[M+H]+1	0.2076	0.2235
MET 1071	500.40944	26.054	[M+H]+1	0.0158	0.0022
MET 1072	459.38283	26.055	[M+H]+1	0.0519	0.0103
MET 1073	441.37218	26.056	[M+H]+1	0.1321	0.0254
MET 1074	409.34604	26.107	[M+H]+1	0.0658	0.0442
MET 1075	556.44135	26.214	[M+NH4]+1	0.0460	0.0491
MET 1076	441.30099	26.236	[M-H]-1	0.0033	0.0025
MET 1077	567.29603	26.278	[M+H]+1	0.0135	0.0091
MET 1078	480.30953	26.33	[M-H]-1	0.0556	0.0506
MET 1079	468.38911	26.35	[M+NH4]+1	0.0563	0.0553
MET 1080	254.24753	26.35	[M+NH4]+1	0.2736	0.1894
MET 1081	844.38792	26.541	[M+2H]+2	0.0074	0.0028
MET 1082	299.25766	26.613	[M+H]+1	0.0806	0.1278
MET 1083	477.34326	26.656	[M-H]-1	0.0050	0.0085
MET 1084	553.35914	26.746	[M-H]-1	0.0041	0.0040
MET 1085	441.33589	26.877	[M+H]+1	0.0159	0.0159
MET 1086	887.40643	26.953	[M+2H]+2	0.0080	0.0033
MET 1087	319.28387	27.009	[M+H]+1	0.0111	0.0104
MET 1088	275.25776	27.039	[M+H]+1	0.0062	0.0061
MET 1089	650.2227	27.059	[M+NH4]+1	0.0114	0.0111
MET 1090	409.34607	27.068	[M+H]+1	0.0898	0.0475
MET 1091	327.09334	27.079	[M-H]-1	0.0133	0.0136
MET 1092	521.33314	27.082	[M-H]-1	0.0380	0.0354
MET 1093	512.33813	27.216	[M-H]-1	0.0050	0.0035
MET 1094	559.36425	27.326	[M-H]-1	0.0071	0.0059
MET 1095	464.31447	27.341	[M-H]-1	0.0309	0.0450
MET 1096	280.26312	27.356	[M+NH4]+1	0.2744	0.1934
MET 1097	212.1643	27.358	[M+H]+1	0.0128	0.0090
MET 1098	930.42476	27.363	[M+2H]+2	0.0066	0.0030
MET 1099	300.28926	27.545	[M+H]+1	0.0109	0.0146
MET 1100	524.37073	27.598	[M+H]+1	0.0367	0.0383
MET 1101	568.36211	27.6	[M-H]-1	0.0366	0.0403
MET 1102	547.34901	27.745	[M-H]-1	0.0328	0.0354
MET 1103	405.30104	27.881	[M-H]-1	0.0447	0.0349
MET 1104	268.26311	28.04	[M+H]+1	0.0506	0.0360
MET 1105	271.22786	28.118	[M-H]-1	0.3565	0.3497
MET 1106	339.21525	28.119	[M-H]-1	0.0160	0.0160
MET 1107	344.24416	28.119	[M-H]-1	0.0271	0.0252
MET 1108	567.40417	28.287	[M+H-H2O]+1	0.0141	0.0024
MET 1109	427.35666	28.299	[M+H]+1	0.3071	0.1471
MET 1110	326.30494	28.43	[M+H]+1	0.0114	0.0120
MET 1111	398.23216	28.474	[M+H]+1	0.0430	0.0418
MET 1112	513.32229	28.688	[M-H]-1	0.0204	0.0129
MET 1113	451.32017	28.689	[M+H]+1	0.0042	0.0025

International Journal of *Molecular Sciences*

MDPI

Supplementary Table S4. Examining the effects of *Salmonella* phage on the caecal metabolome in *Salmonella*-infected broilers. Bayesian statistical analysis of the relevant metaboltes identified by partial least square-discriminant analysis (PLS-DA) in phage-treated chickens compared with the control group, computed as phage-treated vs control. Phage-treated group received 0.1 % *Salmonella* phage (108 PFU/g) via feed. The control group did not receive a phage

**Superclass**	**Class**	**Subclass**	**Metabolite**	**Formula**	**Ion**	**HPD95 phage-control**	**P0phage-control**	**D phage-control**
Benzenoids	Benzene and substituited derivates	Phenyl methylcarbamates	2-(Ethylsulfonylmethyl)phenyl methylcarbamate	C11H15NO4S	[M-H]-	[0.3,1.8]	99.47	1.03
		Phenylpropanes	1-Methoxy-1-(2,4,5-trimethoxyphenyl)-2-propanol	C13H20O5	[M-H2O-H]-	[-1.36,0.34]	88.36	-0.50
Lipids and lipid-like molecules	Fatty acyls	Eicosanoids	9-deoxy-9-methylene-PGE2	C21H34O4	[M-H]-	[-1.43,0.27]	92.19	-0.60
			15-keto-Prostaglandin E2	C20H30O5	[M-H]-	[-0.87,0.91]	53.02	0.03
		Fatty alcohols	Persenone A	C23H38O4	[M-H]-	[0.54,1.93]	99.88	1.21
	Sterol lipids	Stigmasterols and C24-ethyl derivatives	Norselic acid B	C29H44O4	[M+FA-H]-	[-0.92,0.86]	56.98	-0.08
			5alpha,8alpha-epidioxy-stigmasta-6,9(11),22E-trien-3beta-ol	C29H44O6	[M+H]+	[-1.73,-0.18]	98.92	-0.93
		Cholesterol and derivates	9,11alpha-epoxy-6alpha-acetoxy-cholest-7-en-3beta,5alpha,19-triol	C29H46O6	[M+H-2H2O]+	[0.37,1.82]	99.80	1.11
		Steroid ester	Estra-1,3,5(10)-triene-3,6beta,17beta-triol triacetate	C24H30O6	[M+H]+	[0.53,1.91]	99.93	1.22
	Steroids and steroid derivates	Sulfate steroids	Pregnanolone sulfate	C21H34O5S	[M+FA-H]-	[-1.82,-0.35]	99.70	-1.08
		Bile acids, alcohols and derivates	3-Oxo-5beta-chola-8(14),11-dien-24-oic Acid	C24H34O3	[M+H]+	[-0.33,1.37]	89.11	0.52
			Perulactone	C30H46O7	[M-H]-	[1.05,2.11]	100.00	1.57
			(25S)-3-oxo-cholest-1,4-dien-26-oic acid	C28H42O3	[M+FA-H]-	[-1.32,0.39]	86.52	-0.47

		Ergosterols and derivates	Momordenol	C29H46O2	[M+ACN+H]+	[-1.24,0.49]	82.77	-0.40
		stigmastanes and derivates	7-Oxostigmasterol	C29H46O2	[M+H]+	[0.82,2.03]	100.00	1.42
	Sphingolipids	Phosphosphingolipids	SM(d18:1/0:0)	C23H49N2O5P	[M+Cl]-	[-1.82,-0.35]	99.69	-1.08
		Ceramides	3-Sulfodeoxycholic acid	C24H40O7S	[M-H]-	[-0.37,1.34]	87.46	0.49
	Prenol lipids	Isoprenoids	(+)-3beta-Hydroxy-ursan-28-oic acid	C30H50O3	[M+ACN+H]+	[-1.71,-0.14]	98.85	-0.92
	Glycerophospholipids	Glycerophosphoethanolamines	PE(14:0/0:0)	C19H40NO7P	[M-H]-	[0.37,1.81]	99.80	1.09
		Glycerophosphocholines	LysoPC(18:2(9Z,12Z)/0:0)	C26H50NO7P	[M+H]+	[-0.33,1.36]	88.93	0.52
			PC(18:2(2E,4E)/0:0)	C26H50NO7P	[M+FA-H]-	[-1.62,-0.03]	97.83	-0.83
Organic acids and derivates	Carboxylic acids and derivates	Amino acids, peptides, and analogues	Yersiniabactin	C21H27N3O4S3	[M+ACN+H]+	[1.73,2.12]	100.00	1.91
		Monocarboxylic acid	1-(3,4-Dihydroxyphenyl)-1-decene-3,5-dione	C16H20O4	[M+ACN+H]+	[-1.11,0.6]	72.98	-0.26
		Carbocylic acid derivates	(S,E)-Lyratol propanoate	C13H20O2	[M+FA-H]-	[0.68,1.98]	99.98	1.33
Organic nitrogen compounds	Organonitrogen compounds	Organic nitroso compounds	3-[(3-Methylbutyl)nitrosoamino]-2-butanone	C9H18N2O2	[M+H]+	[0.82,2.03]	99.99	1.43
		Amines	Stearoylethanolamide	C20H41NO2	[M+FA-H]-	[-0.71,1.05]	67.89	0.20
Organic oxygen compounds	organooxygen compounds	Carbohidrates and carbohydrate conjugates	D-Glucosamine 1-phosphate	C6H14NO8P	[M-H]-	[0.59,1.94]	99.97	1.28
		Carbohydrates and carbohydrate conjugates	Glucosyl (E)-2,6-Dimethyl-2,5-heptadienoate	C16H18N3O5	[M+Na-2H]-	[-1.88,-0.46]	99.90	-1.18
Organoheterocyclic compounds	Tetrapyrroles and derivates	Bilirubins	Mesobilirubinogen	C33H44N4O6	[M+H]+	[0.18,1.71]	99.22	0.95
Phenylpropanoids and polykeides	Stilbenes	Stilbenes	Batatasin III	C16H18O3	[M+CH3COO]-	[-1.84,-0.37]	99.73	-1.09
	Flavonoids	Falavans	Kaempferol 7,4'-dimethyl ether 3-(6''-(E)-p-coumarylglucoside)	C32H30O13	[M-H]-	[1.27,2.15]	100.00	1.71
Non-Indentified metabolite 66						[0.24,1.77]	99.31	0.97
Non-Indentified metabolite 101						[0.24,1.77]	99.35	0.99
Non-Indentified metabolite 102						[0.09,1.65]	98.78	0.89
Non-Indentified metabolite 116						[0.03,1.63]	97.95	0.85
Non-Indentified metabolite 117						[0.03,1.65]	97.59	0.82
Non-Indentified metabolite 122						[-0.01,1.6]	97.28	0.80

Non-Indentified metabolite 132	[-1.89,-0.51]	99.95	-1.20
Non-Indentified metabolite 195	[0.33,1.8]	99.62	1.06
Non-Indentified metabolite 196	[0.48,1.9]	99.87	1.17
Non-Indentified metabolite 199	[0.26,1.76]	99.39	0.99
Non-Indentified metabolite 210	[-1.9,-0.53]	99.95	-1.24
Non-Indentified metabolite 214	[-1.97,-0.64]	99.94	-1.28
Non-Indentified metabolite 216	[-2,-0.74]	99.98	-1.37
Non-Indentified metabolite 217	[0.25,1.77]	99.40	1.02
Non-Indentified metabolite 266	[-2.14,-1.27]	100.00	-1.70
Non-Indentified metabolite 267	[-2.13,-1.4]	100.00	-1.78
Non-Indentified metabolite 384	[-2,-0.74]	99.99	-1.36
Non-Indentified metabolite 386	[0.48,1.87]	99.90	1.17
Non-Indentified metabolite 394	[-1.75,-0.24]	99.37	-1.00
Non-Indentified metabolite 398	[1.63,2.13]	100.00	1.88
Non-Indentified metabolite 399	[1.66,2.13]	100.00	1.89
Non-Indentified metabolite 400	[1.76,2.09]	100.00	1.93
Non-Indentified metabolite 402	[1.74,2.1]	100.00	1.92
Non-Indentified metabolite 433	[0.64,1.96]	99.99	1.31
Non-Indentified metabolite 434	[0.6,1.96]	99.94	1.26
Non-Indentified metabolite 436	[0.62,1.95]	99.96	1.28
Non-Indentified metabolite 449	[1.52,2.14]	100.00	1.83
Non-Indentified metabolite 462	[-1.76,-0.21]	99.26	-0.98
Non-Indentified metabolite 463	[-1.78,-0.29]	99.53	-1.02
Non-Indentified metabolite 465	[0.99,2.1]	100.00	1.54
Non-Indentified metabolite 466	[-2.06,-0.89]	100.00	-1.46
Non-Indentified metabolite 501	[-2.12,-1.16]	100.00	-1.64
Non-Indentified metabolite 506	[-1.98,-0.69]	99.98	-1.33
Non-Indentified metabolite 509	[-1.85,-0.41]	99.87	-1.14
Non-Indentified metabolite 510	[-1.89,-0.46]	99.83	-1.15
Non-Indentified metabolite 530	[-1.89,-0.51]	99.94	-1.22

Non-Indentified metabolite 532	[0.3,1.78]	99.55	1.05
Non-Indentified metabolite 533	[0.64,1.96]	99.97	1.30
Non-Indentified metabolite 536	[-2.13,-1.21]	100.00	-1.68
Non-Indentified metabolite 571	[0.12,1.68]	98.98	0.92
Non-Indentified metabolite 586	[0.37,1.81]	99.83	1.10
Non-Indentified metabolite 615	[0.31,1.8]	99.58	1.06
Non-Indentified metabolite 616	[0.28,1.78]	99.48	1.01
Non-Indentified metabolite 617	[0.28,1.79]	99.51	1.04
Non-Indentified metabolite 633	[-1.88,-0.46]	99.88	-1.17
Non-Indentified metabolite 660	[-1.6,-0.02]	97.86	-0.81
Non-Indentified metabolite 661	[0.02,1.62]	97.70	0.82
Non-Indentified metabolite 664	[-1.54,0.12]	95.54	-0.72
Non-Indentified metabolite 665	[-1.56,0.03]	97.16	-0.77
Non-Indentified metabolite 666	[-1.43,0.27]	92.68	-0.61
Non-Indentified metabolite 667	[-0.72,1.03]	63.55	0.15
Non-Indentified metabolite 669	[-0.94,0.8]	55.14	-0.05
Non-Indentified metabolite 680	[0.37,1.84]	99.72	1.08
Non-Indentified metabolite 692	[-1.6,0.01]	97.31	-0.79
Non-Indentified metabolite 695	[-1.61,-0.03]	98.05	-0.84
Non-Indentified metabolite 724	[0.56,1.95]	99.92	1.22
Non-Indentified metabolite 748	[0.19,1.71]	99.17	0.95
Non-Indentified metabolite 813	[0.66,1.96]	99.97	1.31
Non-Indentified metabolite 822	[0.54,1.9]	99.92	1.22
Non-Indentified metabolite 823	[0.5,1.9]	99.85	1.20
Non-Indentified metabolite 856	[1,2.09]	100.00	1.54
Non-Indentified metabolite 859	[-2,-0.79]	99.99	-1.41
Non-Indentified metabolite 860	[-1.93,-0.59]	99.96	-1.28
Non-Indentified metabolite 866	[0.54,1.91]	99.94	1.24
Non-Indentified metabolite 872	[0.44,1.85]	99.86	1.17
Non-Indentified metabolite 889	[0.24,1.77]	99.17	0.98

Non-Indentified metabolite 893	[-2.02,-0.81]	99.99	-1.43
Non-Indentified metabolite 897	[0.51,1.89]	99.93	1.23
Non-Indentified metabolite 901	[0.29,1.79]	99.59	1.04
Non-Indentified metabolite 903	[0.64,1.95]	99.97	1.31
Non-Indentified metabolite 904	[0.66,1.97]	99.98	1.32
Non-Indentified metabolite 941	[0.68,1.99]	99.97	1.31
Non-Indentified metabolite 964	[0.61,1.94]	99.98	1.26
Non-Indentified metabolite 970	[0.79,2.01]	99.99	1.41
Non-Indentified metabolite 977	[-1.87,-0.45]	99.86	-1.18
Non-Indentified metabolite 989	[0.33,1.8]	99.62	1.06
Non-Indentified metabolite 1003	[1.2,2.11]	100.00	1.67
Non-Indentified metabolite 1010	[-0.95,0.8]	54.83	-0.05
Non-Indentified metabolite 1016	[-0.83,0.92]	57.78	0.08
Non-Indentified metabolite 1019	[-1.33,0.38]	87.88	-0.50
Non-Indentified metabolite 1020	[1.04,2.08]	100.00	1.56
Non-Indentified metabolite 1025	[0.8,2.04]	99.99	1.41
Non-Indentified metabolite 1026	[-1.21,0.53]	77.04	-0.32
Non-Indentified metabolite 1027	[-0.52,1.17]	77.04	0.31
Non-Indentified metabolite 1029	[-0.41,1.28]	86.44	0.46
Non-Indentified metabolite 1037	[0.32,1.8]	99.58	1.06
Non-Indentified metabolite 1080	[-1.69,-0.16]	98.82	-0.91

HPD95phage-control = The highest posterior density region at 95% of probability. P0= Probability of the difference (Dphage-control) being greater than 0 when Dphage-control > 0 or lower than 0 when Dphage-control < 0. Dphage-control = Mean of the difference - phage-treated-control (median of the marginal posterior distribution of the difference between the control group phage-treated group). Statistical differences were assumed if ∣ Dphage-control ∣ surpass R value and its P0>0.90.

International Journal of *Molecular Sciences*

MDPI

Supplementary Table S5. Examining the effects of *Salmonella* phage on the caecal metabolome in *Salmonella*-infected broilers. Key non-identified metabolites identified by partial least square-discriminant analysis (PLS-DA) for discriminating between groups with relevant differences in mean abundance based on Bayesian statistical analysis in phage-treated broilers compared with the control group, computed as phage-treated *vs* control. Phage-treated group received a 0.1 % *Salmonella* phage (10^8 PFU/g) *via* feed. The control group did not receive a phage.

Metabolite	$HPD95_{phage-control}$	$P0_{phage-control}$	$D_{phage-control}$
Non-Identified metabolite 66	[0.24,1.77]	99.31	0.97
Non-Identified metabolite 101	[0.24,1.77]	99.35	0.99
Non-Identified metabolite 102	[0.09,1.65]	98.78	0.89
Non-Identified metabolite 116	[0.03,1.63]	97.95	0.85
Non-Identified metabolite 117	[0.03,1.65]	97.59	0.82
Non-Identified metabolite 122	[-0.01,1.6]	97.28	0.80
Non-Identified metabolite 132	[-1.89,-0.51]	99.95	-1.20
Non-Identified metabolite 195	[0.33,1.8]	99.62	1.06
Non-Identified metabolite 196	[0.48,1.9]	99.87	1.17
Non-Identified metabolite 199	[0.26,1.76]	99.39	0.99
Non-Identified metabolite 210	[-1.9,-0.53]	99.95	-1.24
Non-Identified metabolite 214	[-1.97,-0.64]	99.94	-1.28
Non-Identified metabolite 216	[-2,-0.74]	99.98	-1.37
Non-Identified metabolite 217	[0.25,1.77]	99.40	1.02
Non-Identified metabolite 266	[-2.14,-1.27]	100.00	-1.70
Non-Identified metabolite 267	[-2.13,-1.4]	100.00	-1.78
Non-Identified metabolite 384	[-2,-0.74]	99.99	-1.36
Non-Identified metabolite 386	[0.48,1.87]	99.90	1.17
Non-Identified metabolite 394	[-1.75,-0.24]	99.37	-1.00
Non-Identified metabolite 398	[1.63,2.13]	100.00	1.88
Non-Identified metabolite 399	[1.66,2.13]	100.00	1.89
Non-Identified metabolite 400	[1.76,2.09]	100.00	1.93
Non-Identified metabolite 402	[1.74,2.1]	100.00	1.92
Non-Identified metabolite 433	[0.64,1.96]	99.99	1.31
Non-Identified metabolite 434	[0.6,1.96]	99.94	1.26
Non-Identified metabolite 436	[0.62,1.95]	99.96	1.28
Non-Identified metabolite 449	[1.52,2.14]	100.00	1.83
Non-Identified metabolite 462	[-1.76,-0.21]	99.26	-0.98
Non-Identified metabolite 463	[-1.78,-0.29]	99.53	-1.02
Non-Identified metabolite 465	[0.99,2.1]	100.00	1.54
Non-Identified metabolite 466	[-2.06,-0.89]	100.00	-1.46
Non-Identified metabolite 501	[-2.12,-1.16]	100.00	-1.64
Non-Identified metabolite 506	[-1.98,-0.69]	99.98	-1.33
Non-Identified metabolite 509	[-1.85,-0.41]	99.87	-1.14
Non-Identified metabolite 510	[-1.89,-0.46]	99.83	-1.15
Non-Identified metabolite 530	[-1.89,-0.51]	99.94	-1.22
Non-Identified metabolite 532	[0.3,1.78]	99.55	1.05
Non-Identified metabolite 533	[0.64,1.96]	99.97	1.30
Non-Identified metabolite 536	[-2.13,-1.21]	100.00	-1.68
Non-Identified metabolite 571	[0.12,1.68]	98.98	0.92
Non-Identified metabolite 586	[0.37,1.81]	99.83	1.10
Non-Identified metabolite 615	[0.31,1.8]	99.58	1.06
Non-Identified metabolite 616	[0.28,1.78]	99.48	1.01
Non-Identified metabolite 617	[0.28,1.79]	99.51	1.04
Non-Identified metabolite 633	[-1.88,-0.46]	99.88	-1.17
Non-Identified metabolite 660	[-1.6,-0.02]	97.86	-0.81
Non-Identified metabolite 661	[0.02,1.62]	97.70	0.82
Non-Identified metabolite 664	[-1.54,0.12]	95.54	-0.72
Non-Identified metabolite 665	[-1.56,0.03]	97.16	-0.77
Non-Identified metabolite 666	[-1.43,0.27]	92.68	-0.61
Non-Identified metabolite 680	[0.37,1.84]	99.72	1.08
Non-Identified metabolite 692	[-1.6,0.01]	97.31	-0.79
Non-Identified metabolite 695	[-1.61,-0.03]	98.05	-0.84
Non-Identified metabolite 724	[0.56,1.95]	99.92	1.22
Non-Identified metabolite 748	[0.19,1.71]	99.17	0.95
Non-Identified metabolite 813	[0.66,1.96]	99.97	1.31
Non-Identified metabolite 822	[0.54,1.9]	99.92	1.22
Non-Identified metabolite 823	[0.5,1.9]	99.85	1.20

Non-Identified metabolite 856	[1,2.09]	100.00	1.54
Non-Identified metabolite 859	[-2,-0.79]	99.99	-1.41
Non-Identified metabolite 860	[-1.93,-0.59]	99.96	-1.28
Non-Identified metabolite 866	[0.54,1.91]	99.94	1.24
Non-Identified metabolite 872	[0.44,1.85]	99.86	1.17
Non-Identified metabolite 889	[0.24,1.77]	99.17	0.98
Non-Identified metabolite 893	[-2.02,-0.81]	99.99	-1.43
Non-Identified metabolite 897	[0.51,1.89]	99.93	1.23
Non-Identified metabolite 901	[0.29,1.79]	99.59	1.04
Non-Identified metabolite 903	[0.64,1.95]	99.97	1.31
Non-Identified metabolite 904	[0.66,1.97]	99.98	1.32
Non-Identified metabolite 941	[0.68,1.99]	99.97	1.31
Non-Identified metabolite 964	[0.61,1.94]	99.98	1.26
Non-Identified metabolite 970	[0.79,2.01]	99.99	1.41
Non-Identified metabolite 977	[-1.87,-0.45]	99.86	-1.18
Non-Identified metabolite 989	[0.33,1.8]	99.62	1.06
Non-Identified metabolite 1003	[1.2,2.11]	100.00	1.67
Non-Identified metabolite 1020	[1.04,2.08]	100.00	1.56
Non-Identified metabolite 1025	[0.8,2.04]	99.99	1.41
Non-Identified metabolite 1037	[0.32,1.8]	99.58	1.06
Non-Identified metabolite 1080	[-1.69,-0.16]	98.82	-0.91

HPD95phage-control = The highest posterior density region at 95% of probability. P0 = Probability of the difference (Dphage-control) being greater than 0 when Dphage-control > 0 or lower than 0 when Dphage-control < 0. Dphage-control = Mean of the difference - phage-treated-control (median of the marginal posterior distribution of the difference between the control group phage-treated group). Statistical differences were assumed if ∣ Dphage-control ∣ surpass R value and its P0 > 0.90.

Supplementary Table S6. Examining the effects of *Salmonella* phage on the serum metabolome in *Salmonella*-infected broilers. Relative abundance (%) of identified serum metabolites.

Met	m/z	RT [min]	Reference Ion	control	phage-treated
MET 1	253.90939	1.016	[M+H]+1	0.0564	0.0155
MET 2	372.88137	1.027	[M+H]+1	0.0562	0.0268
MET 3	170.09227	1.042	[M+H]+1	0.1021	0.0665
MET 4	189.15959	1.066	[M+H]+1	0.0511	0.0510
MET 5	175.11881	1.072	[M+H]+1	0.8242	0.4683
MET 6	1132.79629	1.076	[M-2H]-2	0.0186	0.0167
MET 7	1030.81557	1.076	[M-2H]-2	0.0188	0.0179
MET 8	1064.80969	1.079	[M-2H]-2	0.0240	0.0222
MET 9	1098.80279	1.079	[M-2H]-2	0.0157	0.0139
MET 10	928.83486	1.079	[M-2H]-2	0.0512	0.0530
MET 11	860.84744	1.08	[M-2H]-2	0.0849	0.0840
MET 12	792.85999	1.082	[M-2H]-2	0.1534	0.1417
MET 13	630.94122	1.082	[M+2H]+2	0.0074	0.0059
MET 14	724.87269	1.082	[M-H]-1	0.2742	0.2595
MET 15	792.86027	1.082	[M-H]-1	0.1530	0.1417
MET 16	962.82824	1.082	[M-2H]-2	0.0223	0.0218
MET 17	826.85382	1.084	[M-2H]-2	0.0283	0.0273
MET 18	894.84083	1.085	[M-2H]-2	0.0252	0.0246
MET 19	1076.79377	1.086	[M+2H]+2	0.0171	0.0183
MET 20	1314.74988	1.086	[M+2H]+2	0.0125	0.0126
MET 21	1212.76826	1.086	[M+2H]+2	0.0103	0.0097
MET 22	1038.80293	1.087	[M-2H]-2	0.0196	0.0174
MET 23	656.8853	1.087	[M-H]-1	0.4187	0.3917
MET 24	690.87885	1.088	[M-2H]-2	0.0392	0.0390
MET 25	1072.79563	1.088	[M-2H]-2	0.0184	0.0148
MET 26	622.89133	1.088	[M-2H]-2	0.0325	0.0302
MET 27	600.8819	1.088	[M+2H]+2	0.0366	0.0425
MET 28	872.83113	1.088	[M+2H]+2	0.0335	0.0388
MET 29	668.86915	1.089	[M+2H]+2	0.0503	0.0608
MET 30	1004.8091	1.089	[M-2H]-2	0.0199	0.0181
MET 31	758.86616	1.089	[M-2H]-2	0.0348	0.0348
MET 32	287.06985	1.089	[M+Na]+1	0.0164	0.0143
MET 33	804.84381	1.089	[M+2H]+2	0.0408	0.0478
MET 34	1246.7622	1.09	[M+2H]+2	0.0183	0.0193
MET 35	736.85649	1.09	[M+2H]+2	0.0480	0.0575
MET 36	936.82179	1.091	[M-2H]-2	0.0229	0.0205
MET 37	1254.74874	1.091	[M+2H]+2	0.0077	0.0063
MET 38	1178.77478	1.091	[M+2H]+2	0.0274	0.0308
MET 39	970.81514	1.091	[M-2H]-2	0.0212	0.0196
MET 40	1084.78038	1.091	[M+2H]+2	0.0134	0.0124
MET 41	902.82809	1.092	[M-2H]-2	0.0234	0.0216
MET 42	1110.78745	1.092	[M+2H]+2	0.0404	0.0471
MET 43	834.84076	1.093	[M-2H]-2	0.0239	0.0220
MET 44	907.82802	1.093	[M+2H]+2	0.0177	0.0175
MET 45	1220.75561	1.093	[M+2H]+2	0.0087	0.0072
MET 46	1050.78703	1.093	[M+2H]+2	0.0146	0.0153
MET 47	1186.76125	1.093	[M+2H]+2	0.0096	0.0085
MET 48	1042.79988	1.093	[M+2H]+2	0.0574	0.0674
MET 49	974.81254	1.093	[M+2H]+2	0.0776	0.0916
MET 50	1288.74212	1.094	[M+2H]+2	0.0072	0.0057
MET 51	982.79956	1.094	[M+2H]+2	0.0185	0.0188
MET 52	906.82494	1.094	[M+2H]+2	0.1041	0.1234
MET 53	1016.79338	1.094	[M+2H]+2	0.0168	0.0161

MET 54	880.81822	1.095	[M+2H]+2	0.0262	0.0271
MET 55	698.86584	1.095	[M-2H]-2	0.0278	0.0253
MET 56	948.80568	1.095	[M+2H]+2	0.0216	0.0213
MET 57	664.87246	1.095	[M-2H]-2	0.0246	0.0228
MET 58	770.85013	1.095	[M+2H]+2	0.1732	0.2052
MET 59	588.89766	1.096	[M-H]-1	0.7321	0.6562
MET 60	732.85976	1.096	[M-2H]-2	0.0269	0.0244
MET 61	812.83083	1.096	[M+2H]+2	0.0302	0.0320
MET 62	642.8625	1.096	[M+2H]+2	0.0239	0.0263
MET 63	838.83746	1.096	[M+2H]+2	0.1378	0.1619
MET 64	710.84979	1.096	[M+2H]+2	0.0325	0.0349
MET 65	846.82455	1.096	[M+2H]+2	0.0283	0.0294
MET 66	766.8532	1.096	[M-2H]-2	0.0269	0.0239
MET 67	702.86279	1.096	[M+2H]+2	0.2197	0.2602
MET 68	778.83714	1.097	[M+2H]+2	0.0324	0.0345
MET 69	608.86894	1.097	[M+2H]+2	0.0163	0.0164
MET 70	744.84347	1.097	[M+2H]+2	0.0340	0.0366
MET 71	808.83409	1.097	[M-2H]-2	0.0636	0.0524
MET 72	800.84709	1.097	[M-2H]-2	0.0245	0.0220
MET 73	876.82147	1.097	[M-2H]-2	0.0405	0.0326
MET 74	676.85618	1.098	[M+2H]+2	0.0300	0.0323
MET 75	634.87561	1.099	[M+H]+1	0.2466	0.2909
MET 76	520.90994	1.1	[M-H]-1	0.8359	0.7138
MET 77	1024.78067	1.1	[M+2H]+2	0.0085	0.0072
MET 78	820.81795	1.1	[M+2H]+2	0.0131	0.0119
MET 79	956.79308	1.1	[M+2H]+2	0.0097	0.0085
MET 80	808.83415	1.1	[M-H]-1	0.0636	0.0524
MET 81	1194.74903	1.1	[M+2H]+2	0.0121	0.0103
MET 82	1092.76823	1.101	[M+2H]+2	0.0070	0.0056
MET 83	888.80543	1.102	[M+2H]+2	0.0114	0.0105
MET 84	740.84664	1.102	[M-H]-1	0.1023	0.0858
MET 85	452.92269	1.102	[M-H]-1	10507	0.8778
MET 86	684.84321	1.103	[M+2H]+2	0.0119	0.0108
MET 87	634.87551	1.103	[M+2H]+2	0.2469	0.2909
MET 88	384.93526	1.103	[M-H]-1	12283	0.9372
MET 89	702.86286	1.103	[M+H]+1	0.2197	0.2602
MET 90	752.83044	1.103	[M+2H]+2	0.0137	0.0126
MET 91	158.96392	1.103	[M+H]+1	0.3680	0.4598
MET 92	672.85936	1.105	[M-H]-1	0.1394	0.1180
MET 93	1126.76149	1.105	[M+2H]+2	0.0153	0.0147
MET 94	226.95122	1.105	[M+H]+1	14927	16879
MET 95	294.93851	1.106	[M+H]+1	0.4200	0.4803
MET 96	990.78656	1.106	[M+2H]+2	0.0252	0.0241
MET 97	566.88827	1.106	[M+H]+1	0.2800	0.3285
MET 98	1058.77422	1.107	[M+2H]+2	0.0195	0.0187
MET 99	362.92584	1.107	[M+H]+1	0.4939	0.5708
MET 100	316.94762	1.107	[M-H]-1	16208	11352
MET 101	922.79921	1.108	[M+2H]+2	0.0301	0.0301
MET 102	430.91336	1.109	[M+H]+1	0.4101	0.4800
MET 103	854.81153	1.109	[M+2H]+2	0.0356	0.0366
MET 104	786.82419	1.11	[M+2H]+2	0.0433	0.0454
MET 105	498.90083	1.111	[M+H]+1	0.2967	0.3471
MET 106	604.87177	1.112	[M-H]-1	0.1988	0.1571
MET 107	248.96008	1.113	[M-H]-1	14717	0.8846
MET 108	718.83686	1.113	[M+H]+1	0.0503	0.0515
MET 109	536.88404	1.114	[M-H]-1	0.2092	0.1647
MET 110	650.84959	1.115	[M+H]+1	0.0512	0.0525
MET 111	378.89983	1.117	[M+H]+1	0.1005	0.1067
MET 112	310.91242	1.117	[M+H]+1	0.0932	0.0997

MET 113	468.89642	1.118	[M-H]-1	0.2164	0.1593
MET 114	514.87491	1.119	[M+H]+1	0.0604	0.0640
MET 115	582.8623	1.119	[M+H]+1	0.0528	0.0566
MET 116	400.90908	1.12	[M-H]-1	0.1899	0.1318
MET 117	332.92178	1.123	[M-H]-1	0.1809	0.1142
MET 118	264.93426	1.123	[M-H]-1	0.1032	0.0549
MET 119	180.97197	1.125	[M-H]-1	0.5484	0.2548
MET 120	489.95083	1.162	[M+H]+1	0.0080	0.0076
MET 121	285.98837	1.165	[M+H]+1	0.0148	0.0151
MET 122	259.02759	1.175	[M+H]+1	0.0173	0.0175
MET 123	300.00407	1.177	[M+H]+1	0.0106	0.0088
MET 124	348.07928	1.182	[M+H]+1	0.0252	0.0285
MET 125	148.03442	1.193	[M+Na]+1	0.0047	0.0031
MET 126	156.0421	1.194	[M+H]+1	0.3114	0.1957
MET 127	164.02933	1.194	[M+H]+1	0.0255	0.0253
MET 128	266.07461	1.195	[M+Na]+1	0.0080	0.0050
MET 129	280.09189	1.197	[M+H]+1	0.1809	0.2036
MET 130	271.03989	1.198	[M+H]+1	0.0924	0.1011
MET 131	140.06821	1.205	[M+H]+1	16855	11501
MET 132	225.06104	1.205	[M-H]-1	11668	0.4536
MET 133	179.05508	1.21	[M-H]-1	0.2785	0.1318
MET 134	216.92269	1.212	[M+H]+1	0.0255	0.0380
MET 135	219.02654	1.215	[M+H]+1	0.0434	0.0506
MET 136	132.07686	1.215	[M+H]+1	0.2843	0.2102
MET 137	169.98579	1.216	[M+Na]+1	0.0325	0.0526
MET 138	161.04433	1.218	[M-H]-1	0.0967	0.0362
MET 139	205.06821	1.22	[M+H]+1	0.0271	0.0190
MET 140	148.93524	1.225	[M+H]+1	0.0048	0.0044
MET 141	221.18592	1.226	[M+H]+1	0.1310	0.1601
MET 142	257.14708	1.228	[M+H]+1	0.5545	0.4334
MET 143	328.9985	1.23	[M+H]+1	0.0082	0.0104
MET 144	203.0526	1.23	[M+H]+1	0.5594	0.5581
MET 145	235.16512	1.233	[M+H]+1	0.1340	0.2282
MET 146	264.85228	1.234	[M+H]+1	0.0030	0.0030
MET 147	244.07914	1.235	[M+H]+1	0.0218	0.0174
MET 148	383.11584	1.244	[M+H]+1	0.0213	0.0281
MET 149	278.82959	1.244	[M-H]-1	0.0163	0.0188
MET 150	206.89382	1.244	[M+H]+1	0.0121	0.0154
MET 151	382.76653	1.246	[M+H]+1	0.0032	0.0024
MET 152	116.07095	1.246	[M+H]+1	0.4270	0.2445
MET 153	217.04718	1.253	[M-H]-1	0.0135	0.0108
MET 154	343.86148	1.258	[M+H]+1	0.0034	0.0029
MET 155	215.03211	1.258	[M-H]-1	16449	20258
MET 156	218.03242	1.259	[M-H]-1	0.0364	0.0447
MET 157	165.03924	1.26	[M-H]-1	0.0930	0.0538
MET 158	256.05912	1.263	[M-H]-1	0.0093	0.0100
MET 159	160.91417	1.267	[M-H]-1	0.0110	0.0090
MET 160	164.83485	1.268	[M-H]-1	0.0463	0.0204
MET 161	560.59107	1.268	[M-H]-1	0.0123	0.0151
MET 162	336.78843	1.269	[M-H]-1	0.0149	0.0158
MET 163	499.03319	1.269	[M+H]+1	0.0067	0.0073
MET 164	247.17631	1.27	[M+H]+1	0.0219	0.0224
MET 165	392.90216	1.271	[M+H]+1	0.0056	0.0053
MET 166	566.77885	1.271	[M-H]-1	0.0090	0.0092
MET 167	510.81669	1.271	[M+H]+1	0.0056	0.0051
MET 168	502.63258	1.271	[M-H]-1	0.0156	0.0185
MET 169	436.8839	1.271	[M+H]+1	0.0053	0.0050
MET 170	357.83881	1.272	[M-H]-1	0.0095	0.0111
MET 171	376.92826	1.272	[M+H]+1	0.0207	0.0282

MET 172	501.79692	1.272	[M-H]-1	0.0063	0.0066
MET 173	622.74028	1.272	[M-H]-1	0.0110	0.0127
MET 174	318.96961	1.272	[M+H]+1	0.0397	0.0546
MET 175	261.01099	1.273	[M+H]+1	0.0085	0.0084
MET 176	506.62634	1.273	[M-H]-1	0.0078	0.0065
MET 177	494.84271	1.273	[M+H]+1	0.0130	0.0171
MET 178	442.6765	1.274	[M-H]-1	0.0135	0.0168
MET 179	576.56516	1.274	[M-H]-1	0.0066	0.0070
MET 180	564.78142	1.274	[M-H]-1	0.0094	0.0097
MET 181	160.8407	1.274	[M-H]-1	0.1513	0.0651
MET 182	552.80121	1.274	[M+H]+1	0.0049	0.0048
MET 183	193.15465	1.275	[M+H]+1	0.0315	0.0425
MET 184	500.63545	1.275	[M-H]-1	0.0153	0.0183
MET 185	272.79446	1.275	[M+H]+1	0.0080	0.0081
MET 186	272.99137	1.276	[M-H]-1	0.0102	0.0117
MET 187	430.6963	1.277	[M+H]+1	0.0054	0.0046
MET 188	256.82051	1.277	[M+H]+1	0.0188	0.0235
MET 189	152.04706	1.277	[M-H]-1	0.0102	0.0105
MET 190	144.06554	1.278	[M+H]+1	0.0190	0.0095
MET 191	372.73772	1.278	[M+H]+1	0.0085	0.0093
MET 192	196.86504	1.279	[M+H]+1	0.0163	0.0187
MET 193	384.71813	1.28	[M-H]-1	0.0212	0.0265
MET 194	444.67363	1.281	[M-H]-1	0.0134	0.0168
MET 195	326.75945	1.281	[M-H]-1	0.0472	0.0607
MET 196	330.753	1.282	[M+H]+1	0.0051	0.0035
MET 197	138.90646	1.282	[M+H]+1	0.0112	0.0115
MET 198	239.92408	1.283	[M-H]-1	0.0111	0.0143
MET 199	314.779	1.284	[M+H]+1	0.0088	0.0088
MET 200	268.80083	1.284	[M-H]-1	0.1063	0.1402
MET 201	284.77481	1.285	[M-H]-1	0.0228	0.0264
MET 202	260.97925	1.285	[M-H]-1	0.0181	0.0149
MET 203	330.95	1.286	[M-H]-1	0.0251	0.0313
MET 204	342.7334	1.286	[M-H]-1	0.0122	0.0130
MET 205	210.84158	1.289	[M-H]-1	0.0544	0.0681
MET 206	226.81579	1.292	[M-H]-1	0.0106	0.0109
MET 207	154.87945	1.292	[M-H]-1	0.0188	0.0202
MET 208	150.88532	1.294	[M-H]-1	0.0507	0.0584
MET 209	192.98766	1.295	[M-H]-1	0.0167	0.0147
MET 210	172.84135	1.301	[M-H]-1	0.0202	0.0128
MET 211	197.02135	1.317	[M-H]-1	0.0466	0.0650
MET 212	197.80711	1.318	[M-H]-1	32579	10416
MET 213	154.91742	1.322	[M-H]-1	0.0165	0.0136
MET 214	167.01989	1.378	[M-H]-1	0.1000	0.0849
MET 215	400.85263	1.387	[M-H]-1	0.0071	0.0056
MET 216	340.89675	1.391	[M-H]-1	0.0092	0.0084
MET 217	458.81127	1.392	[M-H]-1	0.0047	0.0026
MET 218	224.97909	1.397	[M-H]-1	0.0333	0.0398
MET 219	282.93822	1.398	[M-H]-1	0.0194	0.0157
MET 220	190.97199	1.41	[M-H]-1	0.0111	0.0039
MET 221	179.01692	1.424	[M+H]+1	0.7278	0.9066
MET 222	157.03512	1.442	[M+H]+1	360779	348982
MET 223	150.05835	1.472	[M+NH4]+1	0.1553	0.1135
MET 224	204.94087	1.503	[M-H]-1	0.0082	0.0031
MET 225	146.98172	1.513	[M-H]-1	0.0111	0.0024
MET 226	348.9158	1.603	[M-H]-1	0.0036	0.0005
MET 227	357.97479	1.623	[M-H]-1	0.0028	0.0008
MET 228	379.9567	1.624	[M-H]-1	0.0033	0.0007
MET 229	279.03894	1.632	[M-H]-1	0.0225	0.0159
MET 230	243.06353	1.633	[M-H]-1	0.0079	0.0061

MET 231	340.90811	1.636	[M-H]-1	0.0035	0.0009
MET 232	185.99304	1.637	[M-H]-1	0.0068	0.0026
MET 233	366.92223	1.638	[M-H]-1	0.0035	0.0008
MET 234	341.02965	1.638	[M-H]-1	0.0062	0.0033
MET 235	182.08108	1.638	[M+NH4]+1	0.8433	0.7991
MET 236	260.09485	1.639	[M+H]+1	0.0193	0.0225
MET 237	378.09646	1.64	[M-H]-1	0.0035	0.0008
MET 238	450.89503	1.64	[M-H]-1	0.0034	0.0008
MET 239	137.04577	1.641	[M+H]+1	0.3871	0.2875
MET 240	136.07568	1.642	[M+NH4]+1	0.0936	0.0793
MET 241	327.92532	1.643	[M-H]-1	0.0038	0.0010
MET 242	428.91303	1.645	[M-H]-1	0.0031	0.0007
MET 243	305.94347	1.645	[M-H]-1	0.0047	0.0020
MET 244	389.91623	1.646	[M-H]-1	0.0029	0.0006
MET 245	333.09408	1.647	[M-H]-1	0.0035	0.0011
MET 246	484.20516	1.647	[M-H]-1	0.0040	0.0010
MET 247	247.98443	1.65	[M-H]-1	0.0065	0.0043
MET 248	288.87696	1.65	[M-H]-1	0.0046	0.0011
MET 249	389.04997	1.651	[M-H]-1	0.0025	0.0006
MET 250	367.93462	1.654	[M-H]-1	0.0034	0.0011
MET 251	337.01874	1.665	[M-H]-1	0.0026	0.0006
MET 252	279.05957	1.666	[M-H]-1	0.0029	0.0011
MET 253	160.07526	1.671	[M+H]+1	0.0192	0.0258
MET 254	318.06102	1.673	[M-H]-1	0.0044	0.0027
MET 255	380.05163	1.676	[M-H]-1	0.0034	0.0012
MET 256	282.08436	1.679	[M-H]-1	0.0039	0.0015
MET 257	210.11572	1.688	[M+H]+1	0.0677	0.0790
MET 258	132.10196	1.699	[M+H]+1	15262	14683
MET 259	396.11612	1.702	[M-H]-1	0.0025	0.0008
MET 260	303.05031	1.709	[M-H-H2O]-1	0.0152	0.0143
MET 261	303.05021	1.709	[M-H]-1	0.0152	0.0143
MET 262	382.99099	1.71	[M-H]-1	0.0027	0.0008
MET 263	444.98154	1.711	[M-H]-1	0.0027	0.0006
MET 264	325.0322	1.712	[M-H]-1	0.0036	0.0019
MET 265	365.04087	1.717	[M-H]-1	0.0056	0.0042
MET 266	387.02309	1.718	[M-H]-1	0.0038	0.0019
MET 267	391.93872	1.718	[M-H]-1	0.0029	0.0006
MET 268	269.08776	1.718	[M+H]+1	0.0579	0.0557
MET 269	272.13511	2.277	[M+H]+1	0.0151	0.0095
MET 270	120.08096	2.387	[M+H]+1	0.3698	0.4864
MET 271	166.08621	2.387	[M+NH4]+1	20896	25678
MET 272	419.04713	2.396	[M-H]-1	0.0017	0.0002
MET 273	429.07557	2.397	[M-H]-1	0.0020	0.0006
MET 274	211.10762	2.477	[M+H]+1	0.0627	0.0729
MET 275	279.13378	2.614	[M+H]+1	0.0075	0.0086
MET 276	311.12355	2.682	[M+H]+1	0.0253	0.0279
MET 277	220.11791	3.093	[M+H]+1	0.0080	0.0083
MET 278	187.08651	3.403	[M+H]+1	0.1025	0.1304
MET 279	357.08306	3.615	[M-H]-1	0.0039	0.0010
MET 280	203.08187	3.95	[M-H]-1	0.0092	0.0053
MET 281	132.08088	3.965	[M+H]+1	0.0064	0.0048
MET 282	146.06003	3.965	[M+H]+1	0.0348	0.0234
MET 283	237.08683	3.965	[M+H]+1	0.0201	0.0213
MET 284	205.09711	3.967	[M+NH4]+1	0.6891	0.8438
MET 285	159.09161	3.968	[M+NH4]+1	0.0102	0.0098
MET 286	144.08077	3.969	[M+H]+1	0.0089	0.0081
MET 287	507.09716	3.97	[M-H]-1	0.0028	0.0009
MET 288	394.01048	3.971	[M-H]-1	0.0051	0.0021
MET 289	407.17254	3.971	[M-H]-1	0.0025	0.0006

MET 290	351.01306	3.972	[M-H]-1	0.0048	0.0015
MET 291	448.93818	3.972	[M-H]-1	0.0039	0.0010
MET 292	429.15438	3.973	[M-H]-1	0.0027	0.0007
MET 293	338.98419	3.973	[M-H]-1	0.0040	0.0014
MET 294	390.01636	3.974	[M-H]-1	0.0040	0.0012
MET 295	465.94039	3.974	[M-H]-1	0.0039	0.0009
MET 296	417.00052	3.974	[M-H]-1	0.0046	0.0015
MET 297	349.0129	3.974	[M-H]-1	0.0150	0.0117
MET 298	354.96928	3.975	[M-H]-1	0.0039	0.0010
MET 299	333.04074	3.975	[M-H]-1	0.0043	0.0011
MET 300	271.07013	3.976	[M-H]-1	0.0071	0.0035
MET 301	494.94338	3.977	[M-H]-1	0.0039	0.0011
MET 302	355.0671	4.003	[M-H]-1	0.0046	0.0014
MET 303	270.12097	4.208	[M-H]-1	0.0046	0.0011
MET 304	217.09716	4.476	[M+H]+1	0.0049	0.0030
MET 305	209.12844	4.759	[M+H]+1	0.0049	0.0028
MET 306	181.04971	4.793	[M-H]-1	0.0058	0.0021
MET 307	355.06729	4.866	[M-H]-1	0.0039	0.0011
MET 308	259.12992	4.908	[M-H]-1	0.0092	0.0067
MET 309	281.11192	4.911	[M-H]-1	0.0033	0.0008
MET 310	304.10524	5.204	[M-H]-1	0.0039	0.0013
MET 311	197.1285	5.273	[M+H]+1	0.0076	0.0079
MET 312	339.02866	5.273	[M-H]-1	0.0030	0.0007
MET 313	239.1034	5.273	[M-H]-1	0.0050	0.0025
MET 314	242.01257	5.484	[M-H]-1	0.0047	0.0019
MET 315	295.12877	5.63	[M+H]+1	0.0286	0.0370
MET 316	392.03147	5.631	[M-H]-1	0.0033	0.0012
MET 317	315.0964	5.632	[M-H]-1	0.0035	0.0014
MET 318	411.12716	5.691	[M-H]-1	0.0028	0.0007
MET 319	217.09716	5.824	[M+H]+1	0.0049	0.0037
MET 320	369.08277	5.827	[M-H]-1	0.0032	0.0010
MET 321	290.03411	5.955	[M-H]-1	0.0040	0.0027
MET 322	416.63758	5.956	[M+2H]+2	0.0040	0.0015
MET 323	777.34293	5.957	[M-H]-1	0.0043	0.0014
MET 324	502.21455	5.983	[M+2H]+2	0.0046	0.0044
MET 325	246.99155	6.014	[M-H]-1	0.0036	0.0013
MET 326	616.74951	6.286	[M+2H]+2	0.0081	0.0342
MET 327	197.0423	6.442	[M-H]-1	0.0043	0.0014
MET 328	274.98612	6.449	[M-H]-1	0.0050	0.0030
MET 329	235.10767	6.458	[M+H]+1	0.0047	0.0026
MET 330	249.08787	6.459	[M-H]-1	0.0039	0.0014
MET 331	265.06522	6.464	[M-H]-1	0.0076	0.0045
MET 332	364.9901	6.465	[M-H]-1	0.0030	0.0008
MET 333	188.98542	6.597	[M-H]-1	0.0198	0.0127
MET 334	336.12028	6.69	[M-H]-1	0.0028	0.0010
MET 335	334.13962	6.695	[M+H]+1	0.0049	0.0042
MET 336	363.05047	6.769	[M-H]-1	0.0026	0.0005
MET 337	301.04986	6.773	[M-H]-1	0.0054	0.0023
MET 338	297.05495	6.775	[M-H]-1	0.0072	0.0033
MET 339	329.04489	6.776	[M-H]-1	0.0051	0.0021
MET 340	413.20416	6.823	[M-H]-1	0.0026	0.0006
MET 341	230.11532	7.057	[M+NH4]+1	0.0032	0.0011
MET 342	197.04231	7.127	[M-H]-1	0.0055	0.0031
MET 343	363.11996	7.132	[M-H]-1	0.0044	0.0027
MET 344	242.1498	7.352	[M+H]+1	0.0034	0.0019
MET 345	280.07605	7.384	[M-H]-1	0.0030	0.0011
MET 346	172.99031	7.386	[M-H]-1	0.0105	0.0059
MET 347	295.12978	7.432	[M-H]-1	0.0029	0.0012
MET 348	757.2358	7.734	[M-H]-1	0.0050	0.0115

MET 349	377.1354	7.852	[M-H]-1	0.0035	0.0017
MET 350	347.12362	7.853	[M+H]+1	0.0029	0.0014
MET 351	275.02315	7.89	[M-H]-1	0.0031	0.0024
MET 352	203.00115	7.93	[M-H]-1	0.0034	0.0015
MET 353	249.0878	7.936	[M-H]-1	0.0032	0.0011
MET 354	429.08264	8.092	[M-H]-1	0.0037	0.0027
MET 355	211.05355	8.103	[M+H]+1	0.0030	0.0016
MET 356	273.00751	8.202	[M-H]-1	0.0074	0.0072
MET 357	361.13922	8.447	[M+H]+1	0.0066	0.0059
MET 358	459.05001	8.448	[M-H]-1	0.0022	0.0006
MET 359	955.40094	8.508	[M+2H]+2	0.0040	0.0042
MET 360	946.88747	8.511	[M+2H]+2	0.0111	0.0164
MET 361	973.84664	8.517	[M+2H]+2	0.0050	0.0066
MET 362	146.06005	8.533	[M+H]+1	0.0220	0.0154
MET 363	496.15371	8.681	[M+H]+1	0.0034	0.0023
MET 364	206.08152	8.761	[M-H]-1	0.0042	0.0031
MET 365	946.88696	9.155	[M+2H]+2	0.0108	0.0130
MET 366	995.37931	9.156	[M+2H]+2	0.0099	0.0115
MET 367	1013.8254	9.162	[M+2H]+2	0.0090	0.0102
MET 368	1014.32627	9.164	[M+2H]+2	0.0083	0.0088
MET 369	986.86594	9.166	[M+2H]+2	0.0197	0.0241
MET 370	218.2114	9.172	[M+H]+1	0.0053	0.0050
MET 371	245.09283	9.358	[M-H]-1	0.0024	0.0009
MET 372	265.11932	9.484	[M-H]-1	0.0028	0.0012
MET 373	262.23758	9.61	[M+H]+1	0.0033	0.0021
MET 374	404.08097	9.662	[M-H]-1	0.0025	0.0009
MET 375	249.06915	9.901	[M+H-H2O]+1	0.0074	0.0063
MET 376	235.10765	9.904	[M+H]+1	0.0236	0.0242
MET 377	251.08476	9.906	[M+H]+1	0.0553	0.0615
MET 378	219.11277	9.909	[M+H]+1	0.0382	0.0340
MET 379	317.0576	9.91	[M-H]-1	0.0044	0.0031
MET 380	348.99538	9.911	[M-H]-1	0.0051	0.0041
MET 381	396.14407	10.107	[M+H]+1	0.0042	0.0030
MET 382	361.1392	10.429	[M+H]+1	0.0088	0.0093
MET 383	346.12832	10.5	[M+H]+1	0.0028	0.0018
MET 384	329.06669	10.574	[M-H]-1	0.0040	0.0021
MET 385	301.07164	10.621	[M-H]-1	0.0034	0.0018
MET 386	315.08749	10.741	[M-H]-1	0.0035	0.0018
MET 387	398.12452	11.024	[M-H]-1	0.0028	0.0010
MET 388	163.5147	11.321	[M+2H]+2	0.0036	0.0025
MET 389	261.03793	11.323	[M-H]-1	0.0054	0.0052
MET 390	331.03735	11.323	[M+H]+1	0.0036	0.0025
MET 391	193.04973	11.323	[M-H]-1	0.0382	0.0440
MET 392	315.08746	11.523	[M-H]-1	0.0167	0.0162
MET 393	196.02232	11.676	[M+2H]+2	0.0045	0.0039
MET 394	255.065	11.679	[M+H]+1	0.1660	0.1776
MET 395	300.12283	11.683	[M+H]+1	0.0032	0.0016
MET 396	386.15963	11.821	[M+H]+1	0.0110	0.0115
MET 397	315.13374	12.221	[M+H]+1	0.0044	0.0033
MET 398	246.24259	12.224	[M+H]+1	0.0036	0.0026
MET 399	341.14937	12.386	[M+H]+1	0.0059	0.0143
MET 400	408.10869	12.897	[M-H]-1	0.0031	0.0015
MET 401	514.28399	13.528	[M-H]-1	0.0090	0.0099
MET 402	215.12821	13.834	[M-H]-1	0.0763	0.0141
MET 403	191.1641	14.038	[M+H]+1	0.0240	0.0138
MET 404	321.04368	14.308	[M-H]-1	0.0099	0.0097
MET 405	299.0923	14.38	[M-H]-1	0.0071	0.0044
MET 406	514.28409	14.401	[M-H]-1	0.0064	0.0089
MET 407	274.2738	14.65	[M+H]+1	0.1717	0.2117

MET 408	230.24771	14.727	[M+H]+1	0.0105	0.0126
MET 409	318.3	14.823	[M+H]+1	0.0932	0.1258
MET 410	362.32618	14.961	[M+H]+1	0.0200	0.0277
MET 411	406.35245	15.046	[M+H]+1	0.0062	0.0081
MET 412	203.03011	15.407	[M+2H]+2	0.0794	0.0955
MET 413	296.04	15.413	[M+2H]+2	0.0271	0.0359
MET 414	288.05399	15.415	[M+2H]+2	0.0155	0.0187
MET 415	195.04401	15.417	[M+2H]+2	0.0324	0.0360
MET 416	430.07676	15.419	[M+2H]+2	0.0178	0.0265
MET 417	422.0905	15.419	[M+2H]+2	0.0099	0.0123
MET 418	564.11364	15.42	[M+2H]+2	0.0126	0.0206
MET 419	269.08051	15.42	[M+H]+1	86815	97364
MET 420	556.12734	15.421	[M+2H]+2	0.0079	0.0110
MET 421	548.13879	15.422	[M+2H]+2	0.0031	0.0035
MET 422	637.07856	15.423	[M+H]+1	0.0117	0.0159
MET 423	332.08878	15.425	[M+H]+1	0.0315	0.0282
MET 424	535.13974	15.426	[M-H]-1	0.4374	0.6867
MET 425	299.0922	15.546	[M-H]-1	0.0518	0.0493
MET 426	163.03883	15.561	[M+H]+1	0.0255	0.0333
MET 427	209.08069	15.561	[M+H]+1	0.0156	0.0190
MET 428	315.08745	15.818	[M-H]-1	0.0058	0.0044
MET 429	271.06104	15.907	[M-H]-1	0.0080	0.0078
MET 430	660.34264	16.886	[M-H]-1	0.0062	0.0062
MET 431	346.33138	16.919	[M+H]+1	0.0024	0.0015
MET 432	498.28951	16.959	[M-H]-1	0.0129	0.0144
MET 433	257.08176	16.976	[M-H]-1	0.0396	0.0500
MET 434	283.09631	17.157	[M+H]+1	0.0772	0.1153
MET 435	514.28409	17.375	[M-H]-1	0.0280	0.0261
MET 436	181.05706	17.529	[M+2H]+2	0.0069	0.0063
MET 437	368.01872	17.53	[M+H]+1	0.0044	0.0029
MET 438	172.05186	17.53	[M+2H]+2	0.0064	0.0056
MET 439	250.05596	17.531	[M+2H]+2	0.0038	0.0031
MET 440	192.5652	17.533	[M+2H]+2	0.0047	0.0038
MET 441	223.0964	17.533	[M+H]+1	0.3782	0.3943
MET 442	177.05459	17.533	[M+H]+1	0.3324	0.3551
MET 443	340.02379	17.533	[M+H]+1	0.0054	0.0041
MET 444	180.03796	17.535	[M+2H]+2	0.0268	0.0292
MET 445	285.04042	17.705	[M-H]-1	0.0043	0.0024
MET 446	273.0311	17.806	[M+H]+1	0.0025	0.0014
MET 447	494.25802	17.914	[M-H]-1	0.0094	0.0100
MET 448	280.24809	18.093	[M+NH4]+1	0.0023	0.0009
MET 449	368.30051	18.502	[M+NH4]+1	0.0047	0.0037
MET 450	412.32677	18.616	[M+NH4]+1	0.0060	0.0051
MET 451	456.35301	18.674	[M+NH4]+1	0.0066	0.0055
MET 452	500.37933	18.74	[M+NH4]+1	0.0066	0.0056
MET 453	544.4056	18.798	[M+NH4]+1	0.0063	0.0050
MET 454	588.43183	18.849	[M+NH4]+1	0.0062	0.0045
MET 455	632.45798	18.896	[M+NH4]+1	0.0055	0.0040
MET 456	676.48411	18.942	[M+NH4]+1	0.0050	0.0033
MET 457	551.13487	18.971	[M-H]-1	0.0044	0.0031
MET 458	720.51028	18.983	[M+H]+1	0.0044	0.0029
MET 459	764.53658	19.02	[M+H]+1	0.0035	0.0019
MET 460	808.56167	19.052	[M+H]+1	0.0022	0.0007
MET 461	413.29827	19.055	[M+2H]+2	0.0056	0.0040
MET 462	297.11193	19.07	[M+H]+1	0.0052	0.0088
MET 463	435.31131	19.085	[M+2H]+2	0.0065	0.0050
MET 464	426.79803	19.091	[M+2H]+2	0.0022	0.0008
MET 465	457.3245	19.116	[M+2H]+2	0.0060	0.0046
MET 466	479.33766	19.143	[M+2H]+2	0.0058	0.0044

MET 467	501.35074	19.169	[M+2H]+2	0.0053	0.0038
MET 468	523.36391	19.191	[M+2H]+2	0.0049	0.0034
MET 469	545.377	19.214	[M+2H]+2	0.0043	0.0028
MET 470	567.39008	19.235	[M+2H]+2	0.0037	0.0024
MET 471	589.40319	19.256	[M+2H]+2	0.0033	0.0020
MET 472	611.41628	19.275	[M+2H]+2	0.0029	0.0016
MET 473	633.42947	19.293	[M+2H]+2	0.0027	0.0012
MET 474	535.13984	19.455	[M-H]-1	0.0037	0.0014
MET 475	366.33645	19.617	[M+H]+1	0.0026	0.0015
MET 476	346.2949	19.967	[M+H]+1	0.0240	0.0203
MET 477	191.17934	19.971	[M+H-H2O]+1	0.0852	0.0685
MET 478	457.32473	20.283	[M+2H]+2	0.0027	0.0010
MET 479	496.27388	20.314	[M-H]-1	0.0098	0.0079
MET 480	357.27857	20.386	[M+H-H2O]+1	0.0037	0.0031
MET 481	437.29081	20.388	[M-H]-1	0.0055	0.0044
MET 482	263.20034	20.824	[M+H]+1	0.0246	0.0206
MET 483	468.30838	20.831	[M+H]+1	0.0056	0.0068
MET 484	297.1119	20.909	[M+H]+1	0.0066	0.0093
MET 485	464.28283	20.914	[M+H]+1	0.0268	0.0233
MET 486	498.28938	20.917	[M-H]-1	0.5088	0.4898
MET 487	326.37783	20.952	[M+H]+1	0.0165	0.0315
MET 488	650.71138	21.048	[M+H]+1	0.0068	0.0059
MET 489	648.71349	21.049	[M+H]+1	0.0094	0.0092
MET 490	644.7014	21.05	[M-H]-1	0.0085	0.0064
MET 491	542.32389	21.153	[M+H]+1	0.0069	0.0101
MET 492	586.31513	21.154	[M-H]-1	0.0080	0.0102
MET 493	508.35847	21.577	[M+2H]+2	0.0027	0.0012
MET 494	464.3323	21.591	[M+2H]+2	0.0030	0.0013
MET 495	420.30601	21.607	[M+2H]+2	0.0029	0.0014
MET 496	558.42087	21.611	[M+H]+1	0.0034	0.0016
MET 497	602.44723	21.617	[M+H]+1	0.0030	0.0014
MET 498	646.47341	21.62	[M+H]+1	0.0028	0.0012
MET 499	494.32397	21.66	[M+H]+1	0.0376	0.0529
MET 500	538.31506	21.663	[M-H]-1	0.0429	0.0573
MET 501	500.27828	21.762	[M-H]-1	0.0138	0.0118
MET 502	526.29248	22.093	[M+H]+1	0.0068	0.0073
MET 503	544.26571	22.143	[M-H]-1	0.0055	0.0058
MET 504	476.27808	22.144	[M-H]-1	0.0760	0.0978
MET 505	500.27823	22.162	[M-H]-1	0.0671	0.0913
MET 506	568.26568	22.162	[M-H]-1	0.0061	0.0055
MET 507	412.20933	22.193	[M+H]+1	0.0040	0.0035
MET 508	520.33958	22.195	[M+H]+1	0.0893	0.1270
MET 509	452.24046	22.196	[M+H]+1	0.0043	0.0043
MET 510	287.63281	22.197	[M+2H]+2	0.0030	0.0022
MET 511	564.33057	22.198	[M-H]-1	0.1018	0.1373
MET 512	588.33071	22.208	[M-H]-1	0.0379	0.0470
MET 513	482.32408	22.343	[M+H]+1	0.0053	0.0069
MET 514	526.31469	22.344	[M-H]-1	0.0064	0.0076
MET 515	612.33058	22.53	[M-H]-1	0.0220	0.0251
MET 516	568.3395	22.536	[M+H]+1	0.0216	0.0271
MET 517	291.64655	22.611	[M+2H]+2	0.0095	0.0102
MET 518	1131.66244	22.612	[M-H]-1	0.0059	0.0055
MET 519	436.20904	22.612	[M+H]+1	0.0088	0.0097
MET 520	396.17778	22.612	[M+H]+1	0.0074	0.0076
MET 521	299.63274	22.613	[M+2H]+2	0.0064	0.0070
MET 522	476.24045	22.613	[M+H]+1	0.0106	0.0125
MET 523	588.33064	22.615	[M-H]-1	0.3773	0.4607
MET 524	544.33927	22.627	[M+H]+1	0.4282	0.6098
MET 525	1107.66254	22.65	[M-H]-1	0.0154	0.0236

MET 526	1062.65711	22.657	[M+H]+1	0.0130	0.0247
MET 527	454.29229	22.669	[M+H]+1	0.0064	0.0081
MET 528	520.33936	22.682	[M+H]+1	12337	19213
MET 529	1039.67157	22.682	[M+H]+1	0.0183	0.0450
MET 530	287.63269	22.684	[M+2H]+2	0.0147	0.0213
MET 531	279.6466	22.687	[M+2H]+2	0.0222	0.0293
MET 532	564.33048	22.687	[M-H]-1	11217	14834
MET 533	539.31227	22.687	[M+2H]+2	0.0051	0.0069
MET 534	1083.66243	22.688	[M-H]-1	0.0251	0.0478
MET 535	412.20902	22.691	[M+H]+1	0.0268	0.0357
MET 536	452.2403	22.691	[M+H]+1	0.0331	0.0449
MET 537	636.26107	22.695	[M-H]-1	0.0248	0.0312
MET 538	431.31519	22.696	[M+H]+1	0.0063	0.0064
MET 539	632.31803	22.698	[M-H]-1	0.0543	0.0644
MET 540	520.26545	23.226	[M-H]-1	0.0078	0.0077
MET 541	452.27819	23.227	[M-H]-1	0.0950	0.1242
MET 542	570.35511	23.336	[M+H]+1	0.0162	0.0230
MET 543	614.34623	23.337	[M-H]-1	0.0173	0.0218
MET 544	540.3306	23.351	[M-H]-1	0.1313	0.1631
MET 545	608.31828	23.353	[M-H]-1	0.0091	0.0097
MET 546	620.43632	23.859	[M+H]+1	0.0044	0.0025
MET 547	765.98715	23.935	[M+2H]+2	0.0029	0.0026
MET 548	770.96412	23.939	[M+2H]+2	0.0029	0.0034
MET 549	762.9781	23.94	[M+2H]+2	0.0028	0.0026
MET 550	296.14602	23.941	[M+2H]+2	0.0103	0.0140
MET 551	612.26091	23.943	[M-H]-1	0.0305	0.0395
MET 552	523.29901	23.944	[M+2H]+2	0.0068	0.0094
MET 553	991.67142	23.945	[M+H]+1	0.0288	0.0673
MET 554	540.33055	23.946	[M-H]-1	13925	18235
MET 555	496.33924	23.946	[M+H]+1	15030	22287
MET 556	1002.65897	23.946	[M+2H]+2	0.0029	0.0043
MET 557	515.31257	23.946	[M+2H]+2	0.0065	0.0085
MET 558	1035.66295	23.946	[M-H]-1	0.0390	0.0727
MET 559	306.15153	23.947	[M+2H]+2	0.0073	0.0089
MET 560	608.31821	23.95	[M-H]-1	0.0677	0.0818
MET 561	1103.65024	23.952	[M-H]-1	0.0075	0.0072
MET 562	1041.68692	23.955	[M+H]+1	0.0038	0.0052
MET 563	478.29375	23.974	[M-H]-1	0.0371	0.0460
MET 564	570.35508	24.072	[M+H]+1	0.0187	0.0261
MET 565	244.05782	24.12	[M+H]+1	0.0061	0.0063
MET 566	436.28311	24.121	[M-H]-1	0.0128	0.0162
MET 567	522.35513	24.146	[M+H]+1	0.0512	0.0728
MET 568	566.34621	24.151	[M-H]-1	0.0650	0.0747
MET 569	528.30942	24.312	[M-H]-1	0.0067	0.0066
MET 570	522.37426	24.454	[M+2H]+2	0.0035	0.0018
MET 571	638.27678	24.701	[M-H]-1	0.0175	0.0224
MET 572	566.34623	24.702	[M-H]-1	0.6711	0.8940
MET 573	634.33386	24.702	[M-H]-1	0.0365	0.0471
MET 574	1087.69384	24.704	[M-H]-1	0.0109	0.0193
MET 575	556.31766	24.704	[M-H]-1	0.0137	0.0160
MET 576	541.32844	24.704	[M+2H]+2	0.0030	0.0025
MET 577	522.35518	24.705	[M+H]+1	0.6251	0.9887
MET 578	549.31434	24.705	[M+2H]+2	0.0036	0.0036
MET 579	412.20915	24.706	[M+H]+1	0.0206	0.0307
MET 580	1043.70307	24.707	[M+H]+1	0.0046	0.0098
MET 581	456.20048	24.709	[M-H]-1	0.0121	0.0129
MET 582	572.37075	24.975	[M+H]+1	0.0239	0.0369
MET 583	616.36206	24.975	[M-H]-1	0.0260	0.0360
MET 584	524.33566	24.997	[M-H]-1	0.0073	0.0063

MET 585	482.3603	25.163	[M+H]+1	0.0536	0.0801
MET 586	526.35136	25.165	[M-H]-1	0.0605	0.0812
MET 587	228.23197	25.535	[M+H]+1	0.0490	0.0253
MET 588	548.37079	25.626	[M+H]+1	0.0348	0.0465
MET 589	592.36223	25.628	[M-H]-1	0.0359	0.0438
MET 590	510.3553	25.678	[M+H]+1	0.0107	0.0141
MET 591	480.30947	25.741	[M-H]-1	0.0133	0.0181
MET 592	552.36725	25.888	[M-H]-1	0.0162	0.0201
MET 593	480.3094	26.354	[M-H]-1	0.1538	0.2027
MET 594	580.23484	26.354	[M-H]-1	0.0073	0.0063
MET 595	548.29708	26.356	[M-H]-1	0.0116	0.0120
MET 596	254.24754	26.378	[M+H]+1	0.0411	0.0713
MET 597	560.50327	26.412	[M+H]+1	0.0065	0.0049
MET 598	536.50337	26.614	[M+H]+1	0.0100	0.0116
MET 599	524.37086	26.86	[M+H]+1	0.1082	0.1438
MET 600	568.36206	26.864	[M-H]-1	0.1063	0.1361
MET 601	524.37057	27.59	[M+H]+1	14255	20891
MET 602	1047.73402	27.593	[M+H]+1	0.0243	0.0489
MET 603	568.36187	27.594	[M-H]-1	11483	15032
MET 604	1091.72527	27.594	[M-H]-1	0.0272	0.0449
MET 605	558.33309	27.595	[M-H]-1	0.0222	0.0256
MET 606	640.29247	27.597	[M-H]-1	0.0239	0.0308
MET 607	512.50336	27.629	[M+H]+1	0.0124	0.0118
MET 608	1008.65295	27.673	[M+H]+1	0.0105	0.0157
MET 609	485.28934	27.717	[M+H]+1	0.0676	0.1087
MET 610	986.59848	27.718	[M+H]+1	0.0040	0.0039
MET 611	268.26314	28.063	[M+H]+1	0.0114	0.0138
MET 612	550.38643	28.209	[M+H]+1	0.0212	0.0233
MET 613	594.37794	28.213	[M-H]-1	0.0196	0.0187

International Journal of *Molecular Sciences*

MDPI

Supplementary Table S7. Examining the effects of *Salmonella* phage on the serum metabolome in *Salmonella*-infected broilers. Bayesian statistical analysis of the relevant genera identified by partial least square-discriminant analysis (PLS-DA) in phage-treated chickens compared with the control group, computed as phage-treated vs control. Phage-treated group received 0.1 % *Salmonella* phage (108 PFU/g) via feed. The control group did not receive a phage.

Super Class	Class	Sub Class	Name	Formula	Ion	$HPD95_{phage-control}$	$P0_{phage-control}$	$D_{phage-control}$
Organoheterocyclic compounds	Indoles and derivates	Tryptamines and derivates	5-Methoxytryptophan	C12H14N2O3	[M+H]+1	[-1.8,-0.3]	99.63	-1.05
	Pteridines and derivates	Pterins and derivates	6-Lactoyltetrahydropterin	C9H18C9H13N5O3O8	[M+CH3OH+H]+	[-1,63,-0,03]	97.69	-0.82
	Heteroarene	Polycyclic heteroarene	Indolylmethylthiohydroximate	C10H10N2OS	[M+CH3COO]-	[-1,88,-0,48]	99.92	-1.20
Organic Oxygen compounds	Organooxygen compounds	Carbohydrates and carbohydrate conjugates	D-Mannitol	C6H14O6	[M+H]+1	[-1.7,-0.14]	98.80	-0.92
Organic acids and derivates	Organic sulfuric acids and derivates	Arylsulfates	Dihydroferulic acid 4-sulfate	C10H12O7S	[M-H]-	[-0.58,1.17]	74.45	0.28
	Carboxylic acids and derivates	Amino acids.Peptides. and analogues	Prolyl-Tyrosine	C14H18N2O4	[M+H]+1	[-0.61,1.13]	69.97	0.22
			Alpha-N-Phenylacetyl-L-glutamine-like	C13H16N2O4	[M-H2O-H]-	[-1,17,0,56]	74.40	-0.28
			L-Ornithuric acid	C19 H20 N2 O4	[M+H]+1	[0,35,1,8]	99.69	1.08
Lipids and lipid-like molecules	Steroids and steroid derivates	Bile acids. alcohols and derivates	Murocholic acid	C24H40O4	[M+FA-H]-	[-1.41,0.27]	91.60	-0.58
		Steroid ester	11alpha,17beta-Dihydroxyandrost-4-en-3-one diacetate	C23H32O5	[M+ACN+Na]+	[-1,02,0,74]	62.60	-0.13
	Glycerophospholipids	Glycerophosphoethanolamines	PS(20:3(8Z,11Z,14Z)/0:0)	C26H46NO9P	[M+Na-2H]-	[-1,07,0,67]	66.72	-0.18
			PS(19:0/0:0)	C25H50NO9P	[M-H]-1	[-1,06,0,69]	66.29	-0.19
			PS(18:1(9Z)/0:0)	C24H46NO9P	[M+Na-2H]-	[-0,58,1,15]	73.63	0.27
			PE(6:0/6:0)	C17H34NO8P	[M+H]+1	[-0,4,1,3]	83.83	0.42
			PE(22:6(4Z,7Z,10Z,13Z,16Z,19Z)/0:0)	C27H44NO7P	[M+H]+1	[-0,79,0,97]	54.99	0.06
			PE(20:4(5Z,8Z,11Z,14Z)/0:0)	C25H44NO7P	[M-H]-1	[-0,94,0,82]	55.63	-0.06
			PE(20:4(5Z,8Z,11Z,14Z)/0:0)	C25H44NO7P	[M-H]-1	[-1,19,0,56]	76.35	-0.30
			PE(18:2(9Z,12Z)/0:0)	C23H44NO7P	[M-H]-1	[-0,54,1,2]	77.41	0.32
			PE(17:0/0:0)	C22H46NO7P	[M+H]+1	[-0,26,1,42]	92.76	0.61
			LysoPC(20:5(5Z,8Z,11Z,14Z,17Z))	C28H48NO7P	[M+H]+1	[-0,42,1,28]	84.59	0.43
			LysoPC(18:2)	C26H50NO7P	[M+H]+1	[-0,83,0,91]	53.17	0.03
Phenylpropanoids and Polykeides	Isoflavonoids	O-methylated isoflavonoids	Homoferreirin	C17H16O6	[M-H]-1	[-1.43,0.27]	91.27	-0.57
Non-identified metabolite 50						[-1,7,-0,16]	98.85	-0.93
Non-identified metabolite 154						[-0,76,0,98]	58.28	0.09
Non-identified metabolite 170						[-0,55,1,2]	79.09	0.34
Non-identified metabolite 189						[-1,02,0,72]	63.62	-0.14

Non-identified metabolite 198	[-0,33,1,4]	87.55	0.48
Non-identified metabolite 214	[-1,16,0,59]	76.81	-0.31
Non-identified metabolite 215	[-1,06,0,68]	67.97	-0.20
Non-identified metabolite 216	[-1,04,0,72]	63.43	-0.14
Non-identified metabolite 217	[-1,19,0,54]	78.15	-0.33
Non-identified metabolite 218	[-0,8,0,96]	56.33	0.06
Non-identified metabolite 219	[-1,07,0,67]	67.77	-0.20
Non-identified metabolite 322	[-1,81,-0,32]	99.63	-1.06
Non-identified metabolite 323	[-1,87,-0,42]	99.81	-1.12
Non-identified metabolite 346	[-1,78,-0,29]	99.62	-1.06
Non-identified metabolite 364	[-1,02,0,74]	63.02	-0.14
Non-identified metabolite 374	[-1,12,0,63]	71.18	-0.24
Non-identified metabolite 395	[-1,81,-0,34]	99.65	-1.05
Non-identified metabolite 424	[-1,24,0,49]	82.16	-0.39
Non-identified metabolite 458	[-1,81,-0,32]	99.59	-1.05
Non-identified metabolite 461	[0,27,1,76]	99.51	1.02
Non-identified metabolite 490	[-0,3,1,39]	90.14	0.54
Non-identified metabolite 609	[-0,79,0,96]	57.56	0.08

HPD95phage-control = The highest posterior density region at 95% of probability. P0= Probability of the difference (Dphage-control) being greater than 0 when Dphage-control > 0 or lower than 0 when Dphage-control < 0. Dphage-control = Mean of the difference - phage-treated-control (median of the marginal posterior distribution of the difference between the control group phage-treated group). Statistical differences were assumed if ∣ Dphage-control ∣ surpass R value and its P0>0.90.
